# Supplementary material for: Exploring the role of early-life circumstances, abilities and achievements on well-being at age 50 years: evidence from the 1958 British birth cohort study
Source: BMJ Open. 2020 Feb 20;10(2):e031416. doi: 10.1136/bmjopen-2019-031416 (PMC7044849; doi:10.1136/bmjopen-2019-031416)
Supplement: Supplementary data [file bmjopen-2019-031416supp001.pdf]

Appendix 1

Check for attrition bias between full NCDS sample at birth and Analysis sample

Comparison of distributions of the same birth social class variable:

Father's social class at birth	Full birth survey N=17,415	Valid %	Analysis sample N=8024	Valid %
Class I (Professional)	746	4.5	386	5.2
Class II (Intermediate)	2133	13.0	1127	15.1
Class III (Skilled Manual/Non-Manual)	9981	60.6	4498	60.1
Class IV (Semi-Skilled Manual)	1995	12.1	871	11.6
Class V (Unskilled Manual)	1616	9.8	603	8.1
Total (valid Sclass)	16471	100.0	7485	100.0
Insufficient data on parental Social Class	944		539	
Total	17415		8024	

(chi-square=1.29, 5d.f., non-significant)

Appendix 2: Description of manifest variables, age CM at data collection; latent variable labelling

Variable	Survey Sweep	Range of recorded codes	Mean	St'd Dev'	% missing	Latent Variable
Early years						
Social class of father (GRO 1951)	Birth	1-6 (1=low)	2.98	0.92	6.7	
Birthweight	Birth	1 <2.5 kg<br>2 >=2.5kg	1.94	0.23	2.8	
Did mother smoke after 4th month of pregnancy	Birth	1-4(No/Variable Med'm/Heavy)	1.7	1.10	3.6	
Sex	Birth	1,2	52.5% female		0	
Mother breastfed child	Age 7	1 No; 2 <1 mth; 3 >1 month	2.15	0.86	10.4	
Problem arithmetic score	Age 7	0-10	5.38	2.43	9.1	Age7cog
Copying designs test age 7	Age 7	0-12	7.19	1.93	9.1	
Draw-A-Man test score	Age 7	0-51	24.40	6.95	10.6	
Southgate reading test score	Age 7	0-30	24.43	6.36	8.9	
Fam difficulties by age 7: Housing (health visitor assess't)	Age 7	0 No; 1 D/K 2Yes	0.17	0.51	9.9	Family Material Difficult's Age 7
Fam difficulties by age 7: Finance	Age 7	0,1,2	0.21	0.53	10.0	
Fam difficulties by age 7: Unempl	Age 7	0,1,2	0.11	0.38	10.0	
NFER general ability test	Age 11	0-80	46.14	15.2	12.0	Age11cog
Reading Comprehension score	Age 11	0-40	16.97	5.95	12.0	
Maths score	Age 11	0-40	18.40	10.1	12.0	
Copying designs test age 11	Age 11	0-12	8.45	1.40	12.2	
Rutter behavioural score (age 7) (mother assess't at each age)	Age 7	0-28	6.13	3.50	9.7	
Rutter behavioural scor (age 11)	Age 11	0-28	6.13	3.41	12.8	
Rutter behavioural score (age 16	Age 16	0-36	3.91	3.42	24.4	
Adolescence						
Reading Comprehension age 16	Age 16	0-40	26.58	6.23	23.1	Age16cog
Maths test age 16	Age 16	0-40	13.83	6.96	23.4	
Teacher assess't English ability	Age 16	1-5	3.44	1.12	22.9	
Teacher assess't Maths ability	Age 16	1-5	3.11	1.19	23.5	
Early Mid-Life						
Social class at age 42	Age 42	1-6 (1=low)	3.99	1.24	13.9	
Highest qual'n (acad or vocat) achieved by age 42	Age 42	0-5 (0=no quals; 1-5=NVQ)	2.68	1.36	0	
Later Mid-Life						
Latter cancellation speed	Age 50	0-800	334.60	87.8	3.5	Age50cog
Word recall	Age 50	0-10	6.60	1.46	2.1	
Delayed word recall	Age 50	0-10	5.49	1.81	2.7	
Animal naming test	Age 50	0-65	22.53	6.22	2.1	
SF-36 phys. health (4 scores: see Appx.3. Summative stats shown)	Age 50	0-100 (each of 4 scores)	315.4	83.8	0.2	PhysWbeing Age 50
SF-36 mental health (4 scores)	Age 50	0-100 (4 scores)	276.6	63.3	0.3	Emot'l Wbeing Age 50
CASP-12 (3 control scores, 3 autonomy, 3 pleasure, 3 self-realisation – see Appx.3)	Age 50	0,1,2,3 (each of 12 scores)	23.94	5.42	0.7	Quality of Life Age 50

Appendix 3. SF-36 scale item number, response options and wording by Model Domain, and Quality of Life based on CASP-12 (v2) items

Physical Health Domain

Sub-domain Gen_hlth (2 items, self-assessed general health)

1. In general, would you say your health is: (1) Excellent; (2) Very good; (3) Good; (4) Fair; (5) Poor
2. Compared to one year ago, how would you rate your health in general now? (1) Much better now than one year ago; (2) Somewhat better now than 1 year ago; (3) About the same; (4) Somewhat worse now than 1 year ago; (5) Much worse now than one year ago

Sub-domain Lim_phys (4 items, health now limiting physical activities)

The following items are about activities you might do during a typical day. Does your health now limit you in these activities? If so, how much? (1) Yes, limited a lot; (2) Yes, limited a little; (3) No, not limited at all

3. Vigorous activities, such as running, lifting heavy objects, participating in strenuous sports
4. Moderate activities, such as moving a table, pushing a vacuum cleaner, bowling, or playing golf? 5. Lifting or carrying groceries?
5. Climbing several flights of stairs?
6. Climbing one flight of stairs?
7. Bending, kneeling, or stooping?
8. Walking more than a mile?
9. Walking several blocks?
10. Walking one block?
11. Bathing or dressing yourself?

21. How much bodily pain have you had during the past 4 weeks? (1) None; (2) Very mild; (3) Mild; (4) Moderate; (5) Severe; (6) Very severe

Emotional Well-Being Domain

These questions are about how you feel and how things have been with you during For each question, please give the one answer that comes closest to the way you have been feeling. How much of the time during the past 4 weeks... (1) All of the time; (2) Most of the time; (3) A good bit of the time; (4) Some of the time; (5) A little of the time; (6) None of the time

23. Did you feel full of pep?
24. Have you been a very nervous person?
25. Have you felt so down in the dumps that nothing could cheer you up?
26. Have you felt calm and peaceful?
27. Did you have a lot of energy?
28. Have you felt downhearted and blue?
29. Did you feel worn out?
30. Have you been a happy person?
31. Did you feel tired?

**SF-36 Item**	**Wording**
1 Self-assessed General Health<br>*(Domain in our model: Phys Health)*	In general, would you say your health is:<br>(1) Excellent; (2) Very good; (3) Good;<br>(4) Fair; (5) Poor
2 Self-assessed General Health compared to one year ago<br>*(Domain: Phys Health)*	2. Compared to one year ago, how would you rate your health in general now?<br>(1) Much better now than one year ago;<br>(2) Somewhat better now than 1 year ago;<br>(3) About the same;<br>(4) Somewhat worse now than 1 year ago;<br>(5) Much worse now than one year ago
Health now limiting specific physical activities:<br><br>3. Vigorous activities?<br>*(Domain: Phys Health)*<br><br>4. Moderate activities?<br>*(Domain: Phys Health)*<br><br>5. Lifting or carrying groceries?<br>*(Domain: Phys Health)*<br>6. Climbing several flights of stairs?<br>*(Domain: Phys Health)*<br>7. Climbing one flight of stairs?<br>*(Domain: Phys Health)*<br>8. Bending, kneeling, or stooping?<br>*(Domain: Phys Health)*<br><br>9. Walking more than a mile?<br>*(Domain: Phys Health)*<br><br>10. Walking several blocks?<br>*(Domain: Phys Health)*<br><br>11. Walking one block?<br>*(Domain: Phys Health)*<br><br>12. Bathing or dressing yourself?<br>*(Domain: Phys Health)*	The following items are about activities you might do during a typical day. Does your health now limit you in these activities? If so, how much?<br>3 Vigorous activities, such as running, lifting heavy objects, participating in strenuous sports<br>(1) Yes, limited a lot;<br>(2) Yes, limited a little;<br>(3) No, not limited at all<br>4. Moderate activities, such as moving a table, pushing a vacuum cleaner, bowling, or playing golf?<br>Answer (1)(2)(3)<br>5. Lifting or carrying groceries?<br>Answer (1)(2)(3)<br>6. Climbing several flights of stairs?<br>Answer (1)(2)(3)<br>7. Climbing one flight of stairs?<br>Answer (1)(2)(3)<br>8. Bending, kneeling, or stooping?<br>Answer (1)(2)(3)<br>9. Walking more than a mile?<br>Answer (1)(2)(3)<br>10. Walking several blocks?<br>Answer (1)(2)(3)<br>11. Walking one block?<br>Answer (1)(2)(3)<br>12. Bathing or dressing yourself?<br>Answer (1)(2)(3)
During the past 4 weeks, have you had any of the following problems with your work or other regular daily activities as a result of your physical health?<br>13. Cut down the amount of time you spent on work or other activities?<br>*(Domain: Phys Health)*<br>14. Accomplished less than you would like	 <br><br><br>13. Cut down the amount of time you spent on work or other activities?<br>(1) Yes (2) No<br>14. Accomplished less than you would like?

(Domain: Phys Health)<br>15. Were limited in the kind of work or other activities<br>*(Domain: Phys Health)*<br>16. Had difficulty performing the work or other activities<br>*(Domain: Phys Health)*	(1) Yes (2) No<br>15. Were limited in the kind of work or other activities<br>(1) Yes (2) No<br><br>16. Had difficulty performing the work or other activities (for example, it took extra effort)<br>(1) Yes (2) No
During the past 4 weeks, have you had any of the following problems with your work or other regular daily activities as a result of any emotional problems (such as feeling depressed or anxious)?<br><br>17. Cut down the amount of time you spent on work or other activities<br>*(Domain: Emotional WBeing)*<br>18. Accomplished less than you would like<br>*(Domain: Emotional WBeing)*<br>19. Didn't do work or other activities as carefully as usual<br>*(Domain: Emotional WBeing)*	 <br><br><br><br><br>17. Cut down the amount of time you spent on work or other activities?<br>(1) Yes (2) No<br>18. Accomplished less than you would like?<br>(1) Yes (2) No<br>19. Didn't do work or other activities as carefully as usual?<br>(1) Yes (2) No
20. During the past 4 weeks, to what extent has your physical health or emotional problems interfered with your normal social activities with family, friends, neighbours, or groups?<br>*(Domain: Emotional WBeing)*	20. During the past 4 weeks, to what extent has your physical health or emotional problems interfered with your normal social activities with family, friends, neighbours, or groups?<br>(1) Not at all;<br>(2) Slightly;<br>(3) Moderately;<br>(4) Quite a bit;<br>(5) Extremely
21. How much bodily pain have you had during the past 4 weeks?<br>*(Domain: Phys Health)*	21. How much bodily pain have you had during the past 4 weeks?<br>(1) None;<br>(2) Very mild;<br>(3) Mild;<br>(4) Moderate;<br>(5) Severe;<br>(6) Very severe
22. During the past 4 weeks, how much did pain interfere with your normal work (including both work outside the home and housework)?<br>*(Domain: Phys Health)*	22. During the past 4 weeks, how much did pain interfere with your normal work (including both work outside the home and housework)?<br>(1) Not at all;<br>(2) A little bit;<br>(3) Moderately;<br>(4) Quite a bit;<br>(5) Extremely
These questions are about how you feel and how things have been with you during the past 4 weeks. For each question, please give the one answer that comes closest to the way you have been feeling.	

How much of the time during the past 4 weeks...<br>23. Did you feel full of pep?<br>*(Domain: Emotional WBeing)*<br><br><br><br><br><br><br>24. Have you been a very nervous person?<br>*(Domain: Emotional WBeing)*<br>25. Have you felt so down in the dumps that nothing could cheer you up?<br>*(Domain: Emotional WBeing)*<br>26. Have you felt calm and peaceful?<br>*(Domain: Emotional WBeing)*<br>27. Did you have a lot of energy?<br>*(Domain: Emotional WBeing)*<br>28. Have you felt downhearted and blue?<br>*(Domain: Emotional WBeing)*<br>29. Did you feel worn out?<br>*(Domain: Emotional WBeing)*<br>30. Have you been a happy person?<br>*(Domain: Emotional WBeing)*<br>31. Did you feel tired?<br>*(Domain: Emotional WBeing)*	How much of the time during the past 4 weeks...<br>23. Did you feel full of pep?<br>(1) All of the time;<br>(2) Most of the time;<br>(3) A good bit of the time;<br>(4) Some of the time;<br>(5) A little of the time;<br>(6) None of the time<br>24. Have you been a very nervous person?<br>Answer (1)-(6)<br>25. Have you felt so down in the dumps that nothing could cheer you up?<br>Answer (1)-(6)<br>26. Have you felt calm and peaceful?<br>Answer (1)-(6)<br>27. Did you have a lot of energy?<br>Answer (1)-(6)<br>28. Have you felt downhearted and blue?<br>Answer (1)-(6)<br>29. Did you feel worn out?<br>Answer (1)-(6)<br>30. Have you been a happy person?<br>Answer (1)-(6)<br>31. Did you feel tired?<br>Answer (1)-(6)
32. During the past 4 weeks, how much of the time has your physical health or emotional problems interfered with your social activities (like visiting with friends, relatives, etc.)?<br>*(Domain: Emotional WBeing)*	32. During the past 4 weeks, how much of the time has your physical health or emotional problems interfered with your social activities (like visiting with friends, relatives, etc.)?<br>(1) All of the time;<br>(2) Most of the time;<br>(3) Some of the time;<br>(4) A little of the time;<br>(5) None of the time
How TRUE or FALSE is each of the following statements for you.<br>33. I seem to get sick a little easier than other people<br>*(Domain: Phys Health)*<br><br><br><br><br>34. I am as healthy as anybody I know<br>*(Domain: Phys Health)*<br>35. I expect my health to get worse<br>*(Domain: Phys Health)*<br>36. My health is excellent<br>*(Domain: Phys Health)*	How TRUE or FALSE is each of the following statements for you.<br>33. I seem to get sick a little easier than other people<br>(1) Definitely true;<br>(2) Mostly true;<br>(3) Don't know;<br>(4) Mostly false;<br>(5) Definitely false<br>34. I am as healthy as anybody I know<br>Answer (1)-(5)<br>35. I expect my health to get worse<br>Answer (1)-(5)<br>36. My health is excellent<br>Answer (1)-(5)

Note that the answers to the 36 questions were recoded on a scale from 0 to 100 with 100 indicating the highest levels of health (ie negatively-phrased questions being coded in reverse polarity).

These were then divided into eight sub-domains (four physical health, four emotional well-being, with the recoded variables being summed then divided by the number of questions asked:

Domain	Physical Well-Being		Emotional Well-Being	
Sub-domain	Physical functioning	Pain	Role limitations due to emotional problems	Fatigue
	Role limitations due to physical health	General Health	Emotional Well-Being	Social Functioning

Cronbach's Alpha for the four Physical Well-Being measures is 0.838.

For the four Emotional Well-Being measures, Alpha=0.684.

CASP-12 (v2) Item	Wording
1-3 Control	1 My age prevents me from doing the things I would like to.<br>(1) Often; (2) Sometimes; (3) Not often;<br>(4) Never<br>2 I feel what happens to me is out of my control.<br>Answer (1)-(4)<br>3 I feel left out of things<br>Answer (1)-(4)
4-6 Autonomy	4 I can do the things I want to do.<br>Answer (1)-(4)<br>5 Family responsibil prevent me from doing what want to do.<br>Answer (1)-(4)<br>6 I feel that I can please myself what I do.<br>Answer (1)-(4)<br>6? Shortage of money stops CM doing things wants to do.<br>Answer (1)-(4)
7-9 Self-Realisation	7 I look forward to each day.<br>Answer (1)-(4)<br>8 I feel that my life has meaning.<br>Answer (1)-(4)<br>9 I enjoy the things that I do.<br>Answer (1)-(4)<br>9? On balance I look back on life with sense of happiness.<br>Answer (1)-(4)
10-12 Pleasure	10 I feel full of energy these days.<br>Answer (1)-(4)<br>11 I feel that life is full of opportunities.<br>Answer (1)-(4)<br>12 I feel that the future looks good for me.<br>Answer (1)-(4)

Cronbach's Alpha for the twelve CASP items is 0.861.

Appendix 4 Rutter Behaviour scale, NCDS aged 16 (18-point version asked to mother)

Below are a series of descriptions of behaviour often shown by young people. Please ask the mother about each one and ring the appropriate number to show the degree to which this description is true of the study child:

0 Does not apply
1 Applies somewhat
2 Certainly applies

1	Restless, difficulty staying seated
2	Squirmy,fidgety child
3	Often destroys others property
4	Frequently fights, quarrelsome
5	Not much liked by other children
6	Often worries about things
7	Does things on own, rather solitary
8	Irritable, flies off the handle
9	Appears miserable,unhappy & tearful
10	Twitches,mannerisms,tics-face or body
11	Frequently sucks thumb or fingers
12	Frequently bites nails or fingers
13	Is often disobedient
14	Cannot settle more than a few moments
15	Fearful of new situations or things
16	Fussy, over particular
17	Often tells lies
18	Bullies other children

Max score=36

Cronbach's Alpha for age 16 Rutter Scale = 0.687.
Age 11 Rutter scale has 14 items; Alpha=0.682.
Age 7 Rutter scale has 14 items; Alpha=0.669.

Appendix 5:

Measurement models for the eight latent variables in the SEM analysis

(a) FAMDIFFS: Family material difficulties at Age 7 years (CFI=1.00, TLI=1.00, RMSEA=0.00)

	Indicator loading	Standard Error
Housing	0.46	0.02
Finance	0.79	0.02
Unemployment	0.69	0.02

(b) Age7cog: Cognition at Age 7 years (0.96, 0.88, 0.105)

Arithmetic	0.67	0.01
Copying designs	0.44	0.01
Draw-A-Man	0.48	0.01
Southgate reading	0.69	0.01

(c) Age11Cog: Cognition at age 11 years (1.0, 0.99, 0.018)

NFER General ability	0.78	0.01
Reading comprehens'n	0.65	0.01
Maths	0.79	0.01
Copying designs	0.12	0.01

(d) Age16cog: Cognition at Age 16 years (0.97, 0.91, 0.172)

Reading comprehens'n	0.82	0.01
Mathematics	0.83	0.00
Teacher ass't Eng Abil	0.85	0.00
Teacher ass't Math Abil	0.86	0.01

(e) Age50Cog: Cognition at age 50 years (0.98, 0.94, 0.068)

Letter Cancel'n Speed	0.12	0.01
Word Recall	0.80	0.01
Delayed word recall	0.80	0.01
Animal naming	0.85	0.01

(f) PWB: Physical Well-Being at age 50 years (0.99, 0.97, 0.07)

Physfunc	0.73	0.01
Lim_Phys	0.77	0.01
Pain	0.82	0.01
Gen_hlth	0.76	0.01

(g) EWB: Emotional Well-Being at age 50 years (0.99, 0.95, 0.077)

Lim_Emot	0.62	0.01
Fatig	0.86	0.01
Em_WB	0.84	0.01
Soc_func	0.12	0.02

(h) CASP12: Quality of Life at age 50 years (0.88, 0.85, 0.088)

Control1	0.39	0.01
Control2	0.55	0.01
Control3	0.55	0.01
Autonmy1	0.49	0.01
Autonmy2	0.38	0.01
Autonmy3	0.37	0.01
Pleasur1	0.75	0.01
Pleasur2	0.69	0.01
Pleasur3	0.72	0.01
SlfReal1	0.74	0.01
SlfReal2	0.72	0.01
SlfReal3	0.80	0.01

Appendix 6: Calculation of Indirect Effect and Total Effects in the Structural Equation Model

A Indirect effects from early-life circumstances to Age 50 outcomes (where total effect size >.01 and statistically significant)

Effects from	To	Total effect	Total indirect	Sig. direct effect? (p<.001)	Total effect significant?
SC Birth	Age 50 Cog	0.117	0.117	No	Yes
Birthwt	Age 50 Cog	0.047	0.030	No	Yes
Rutter7	Age 50 Cog	-0.065	-0.071	No	Yes
Rutter11	Age 50 Cog	-0.038	-0.025	No	Yes
Rutter16	Age 50 Cog	-0.018	-0.023	No	Yes
Masmoke	Age 50 Cog	-0.027	-0.027	No	Yes
Breastf	Age 50 Cog	0.039	0.039	No	Yes
SC Birth	PWB	0.081	0.048	No	Yes
Birthwt	PWB	0.011	0.011	No	Yes
Breastf	PWB	0.014	0.014	No	Yes
Rutter7	PWB	-0.043	-0.059	No	Yes
Rutter11	PWB	-0.081	-0.039	No	Yes
Rutter16	PWB	-0.074	-0.002	Yes	Yes
Masmoke	PWB	-0.010	-0.010	No	Yes
Rutter7	EWB	-0.046	-0.046	No	Yes
Rutter11	EWB	-0.080	-0.061	No	Yes
Rutter16	EWB	-0.133	-0.003	Yes	Yes
SC Birth	QoL	0.055	0.033	No	Yes
Rutter7	QoL	-0.048	-0.034	No	Yes
Rutter11	QoL	-0.049	-0.049	No	Yes
Rutter16	QoL	-0.103	-0.004	Yes	Yes

B Indirect effects from early-life circumstances to early mid-life outcomes (total effect size >.01 and statistically significant)

Effects from	To	Total effect	Total indirect	Sig. direct effect? (p<.001)	Total effect significant?
SC Birth	SC42yrs	0.167	0.113	Yes	Yes
SC Birth	Quals	0.184	0.135	Yes	Yes
Birthwt	SC42yrs	0.030	0.030	No	Yes
Birthwt	Quals	0.035	0.035	No	Yes
Masmoke	SC42yrs	-0.027	-0.027	No	Yes
Masmoke	Quals	-0.032	-0.032	No	Yes
Breastf	SC42yrs	0.039	0.039	No	Yes
Breastf	Quals	0.047	0.047	No	Yes

C Indirect effects from early life circumstances to Age 50 outcomes (total effect size <.01 or not statistically significant)

Effects from	to	Total effect	Total indirect	Sig. direct effect? (p<.001)	Total effect significant?
SC Birth	EWB	0.031	0.012	No	No
Birthwt	EWB	0.007	0.004	No	No
Masmoke	EWB	-0.003	-0.003	No	Yes
Breastf	EWB	0.005	0.005	No	Yes
Birthwt	QoL	0.003	0.006	No	No
Masmoke	QoL	-0.005	-0.005	No	Yes
Breastf	QoL	0.008	0.008	No	Yes

Commentary: Three direct influences stand out in Table A, each involving the Rutter scores at age 16 years (see Appx 4).

These are modest direct influences on PWB and QoL, but a relatively stronger negative influence on EWB at age 50 years.

Two direct influences stand out in Table B: from social class at birth to social class at 42 years and Qualifications obtained. The direct influences are shown in our estimated pathways diagram (Fig.2), but we also see in Table C there is an additional indirect effect (via childhood cognition at ages 7/11/16), over twice as large as the direct effect in both cases.

Appendix 7: Separate analyses by sex

Running the analyses separately by sex, we see that certain pathways are significant for one sex but not the other (p<.001) and certain path coefficients are notably different:

Men only (N=3815)

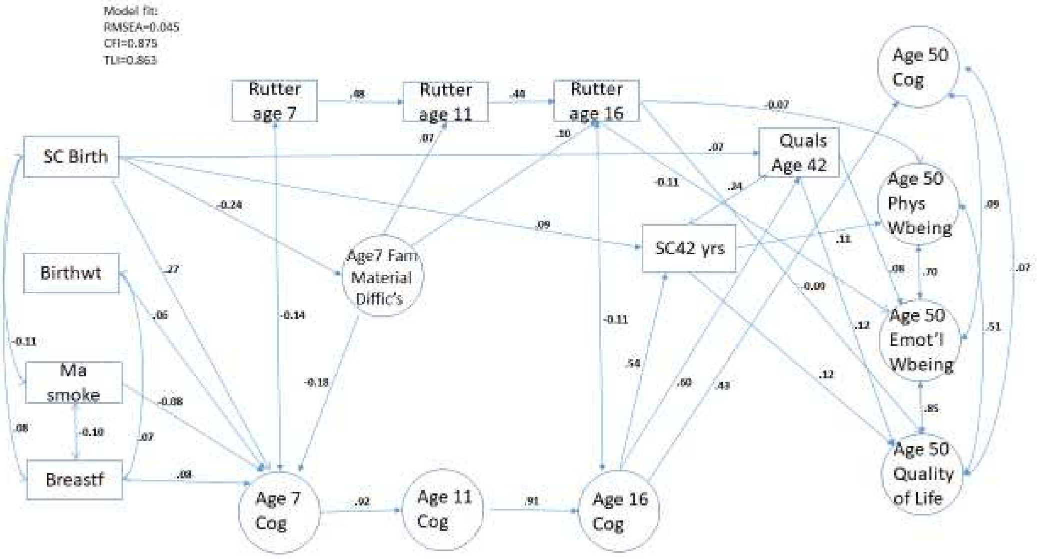

Women only (N=4209)

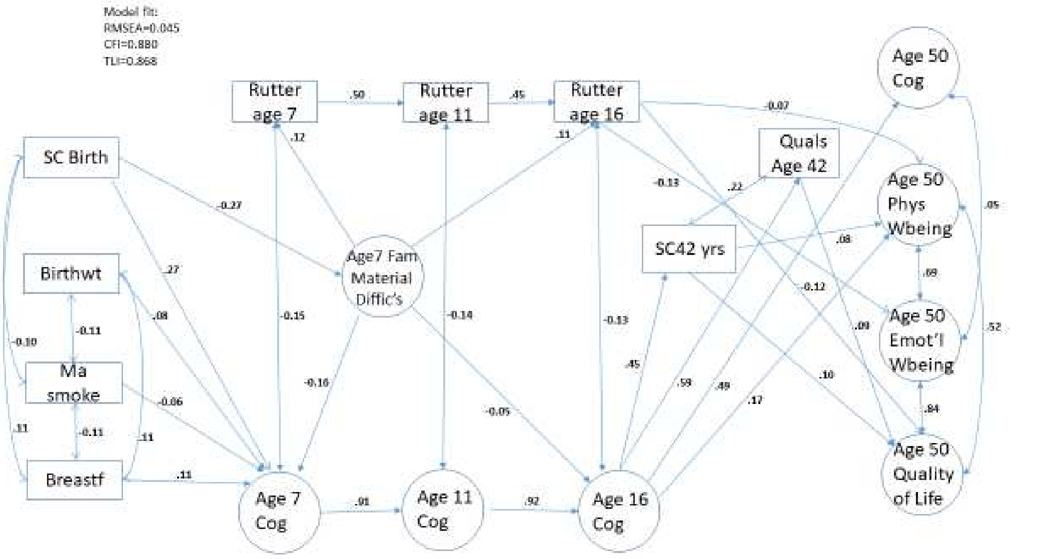

Appendix 8: Multigroup analysis by sex

An MPlus multigroup run by sex was performed as a sensitivity analysis, in which each of the 50 possible paths in Fig.1 was labelled m1-m50, and (in the female model) f1-f50, then a set of 50 'difference' variable was defined diff(i)=m(i)-f(i).

Testing the significance of these 50 'diff' variables, 6 were found to be significant, and the other 43 non-significant.

The MPlus multigroup model was then re-run, constraining all the 44 non-significant paths to be equal for males and females, but allowing all the other six paths to vary, thus increasing the parsimony of the model.

The six paths found to be significantly different between men and women are shown in this table:

	Men: Estimate	P-val	Women: Estimate	P-val
Age 50 Cog on Age 16 Cog	0.43	0.000	0.49	0.000
SC 42yrs on SC Birth	0.09	0.000	0.01	0.461
Phys WBeing on Age 16 Cog	0.07	0.013	0.17	0.000
Age 50 Cog on SC 42yrs	0.06	0.002	-0.02	-0.146
Quals Age 42 on Age 16 Cog	0.60	0.000	0.59	0.000
Age 16 Cog on Age 11 Cog	0.91	0.000	0.92	0.000

Model fit:

RMSEA 0.047
CFI 0.861
TLI 0.855

The first three paths are notable. The fourth is only significant at the .002 level, and in the case of the last two, the difference in the point estimates, though significant, is so small as to be unremarkable.

We see that women's Age 50 cognitive ability has a stronger link with Age 16 cognition (.49) than men's (.43), and that for men there is a direct link from SC Birth to SC 42 yrs (.09), a result not significant for women.

Finally, the link from Age 16 Cog to Phys WBeing is significant for women (.17), a result not significant for men.
