# Supplementary material for: Exploring the role of early-life circumstances, abilities and achievements on well-being at age 50 years: evidence from the 1958 British birth cohort study
Source: BMJ Open. 2020 Feb 20;10(2):e031416. doi: 10.1136/bmjopen-2019-031416 (PMC7044849; doi:10.1136/bmjopen-2019-031416)
Supplement: Author's manuscript [file bmjopen-2019-031416.draft_revisions.pdf]

BMJ Open is committed to open peer review. As part of this commitment we make the peer review history of every article we publish publicly available.

When an article is published we post the peer reviewers' comments and the authors' responses online. We also post the versions of the paper that were used during peer review. These are the versions that the peer review comments apply to.

The versions of the paper that follow are the versions that were submitted during the peer review process. They are not the versions of record or the final published versions. They should not be cited or distributed as the published version of this manuscript.

BMJ Open is an open access journal and the full, final, typeset and author-corrected version of record of the manuscript is available on our site with no access controls, subscription charges or pay-per-view fees (http://bmjopen.bmj.com).

If you have any questions on BMJ Open's open peer review process please email info.bmjopen@bmj.com

BMJ Open

Pathways from early childhood to physical & emotional well-being, quality of life and cognitive function in mid-life

Journal:	*BMJ Open*
Manuscript ID	bmjopen-2019-031416
Article Type:	Research
Date Submitted by the Author:	02-May-2019
Complete List of Authors:	Dodgeon, Brian; UCL Institute of Education Centre for Longitudinal Studies, Social Science<br>Patalay, Praveetha; University College London, Centre for Longitudinal Studies<br>Ploubidis, George; University College London Institute of Education, Centre for Longitudinal Studies<br>Wiggins, Richard; University College London, Centre for Longitudinal Studies
Keywords:	MENTAL HEALTH, PUBLIC HEALTH, STATISTICS & RESEARCH METHODS

SCHOLARONE™
Manuscripts

Pathways from early childhood to physical & emotional well-being, quality of life and cognitive function in mid-life

Corresponding author: Brian Dodgeon, Centre for Longitudinal Studies, UCL Institute of Education, 20 Bedford Way, London WC1H 0AL. b.dodgeon@ucl.ac.uk 020-7612-6877.
ORCID: 0000-0002-7130-8761

Co-authors:
Praveetha Patalay, Centre for Longitudinal Studies, UCL Institute of Education, 20 Bedford Way, London WC1H 0AL United Kingdom.
ORCID: 0000-0002-5341-3461

George B. Ploubidis, Centre for Longitudinal Studies, UCL Institute of Education, 20 Bedford Way, London WC1H 0AL United Kingdom.
ORCID: 0000-0002-8198-5790

Richard D. Wiggins, Centre for Longitudinal Studies, UCL Institute of Education, 20 Bedford Way, London WC1H 0AL United Kingdom.
ORCID: 0000-0007-2648-597X

Contributorship Statement

Brian Dodgeon performed the data management and structural equation modelling using Mplus, and took responsibility for completing all aspects of the paper.

Praveetha Patalay contributed to the conceptual planning of the analysis jointly with Richard Wiggins, performed preliminary regression modelling and advised on aspects of the SEM.

George Ploubidis advised on many aspects of the statistical analysis, and provided detailed comments on the drafting of the paper.

Richard Wiggins undertook the conceptual planning of the analysis jointly with Praveetha Patalay, and initiated the SEM approach, continuing to provide advice on the models as they became more complex, and collaborating with Brian Dodgeon on the write-up of the findings.

Word count: 2,987

What is already known on this subject?

Previous studies have shown that advantaged family socio-economic circumstances during a child's early years, and educational attainment result in favourable later-life outcomes. Separate analyses have shown benefits in physical health; social mobility; cognitive ability; emotional health and quality of life.

What does this study add?

Our multivariate path analysis teases out the nuances of complex pathways through early childhood and adolescence to these distinct mid-life outcomes, assessing the contributions of each and their many interactions.

For peer review only

ABSTRACT

1 Objectives

We aim to examine the relative contributions of pathways from early childhood/adolescence to mid-life well-being, health and cognition, in the context of family socio-economic status (SES), educational achievement and early-adulthood SES. We hypothesise that effects of childhood circumstances on mid-life physical & emotional well-being are strongly mediated by cognitive performance during school years, with stronger mediated links for men than women from childhood-to-adult SES.

2 Setting/Participants

Data were the NCDS Cohort, a representative GB population of 18,558 born in one week in 1958, the setting being GB overall.

3 Primary/secondary outcome measures

Our primary mid-life outcomes are: cognitive performance, physical and emotional well-being and quality of life (QoL), measured using the SF-36 scale and CASP-12v2 QoL scale. Our intermediate outcomes are early-adulthood SES and qualifications.

4 Results

Using structural equation modelling, we examine fifty pathways through childhood and early adulthood, significantly linked to our outcomes. Bonferroni adjustment for 50 pathways means we employ 99% significance levels not 95%, examining the mediating effects of:

- cognitive ability at 7, 11 and 16;
- childhood psychological issues (Rutter);
- family difficulties: housing, unemployment, finance;
- qualifications;
- age 42 SES.

We find birth SES has a strong indirect effect on the age 50 outcomes via its influence on cognitive ability, educational attainment and mid-life SES; also a small direct effect on qualifications and age 42 SES. Teenage cognition has a stronger positive effect on later physical health for women; qualifications have a stronger positive effect on emotional well-being for men.

4 Conclusion

Our findings provide an understanding of the legacy of early life on multiple aspects of mid-life health, well-being, cognition and quality of life, showing stronger mediated links for men from childhood SES to adult SES. The observed effect of qualifications supports those arguing education has a causal effect on subsequent cognitive functioning.

Article Summary

Strengths and limitations of this study:

For peer review only

- The richness of its longitudinal dimension, conveying the importance of early life circumstances upon early adult and mid-life measures of achievement, health and well-being;
- The holistic nature of the analysis, incorporating four outcomes, three of which are strongly correlated after the inclusion our antecedents;
- It makes optimal use of all the available longitudinal data by employing Full Information Maximum Likelihood estimation to compensate for the impact of missing data;
- It shows the enduring importance of parental social class for cognitive performance and later life outcomes, controlling for family difficulties in childhood;
- A limitation is that the subjects of our longitudinal study are not yet old enough for us to examine whether any decline in cognitive performance at age 50 years will become more influential on emotional well-being in future years.

For peer review only

INTRODUCTION

It is well-known that people with advantaged socioeconomic circumstances or status (SES) tend to have better health, across a range of health measures and across different societies[1,2,3]. Most scholars take the view that the mechanism by which SES differences affect health consists of factors such as living and working conditions, access to health services, social relationships and diversified life styles, being unequally distributed across the social hierarchy[1,4]. SES differentials in health may also result from health selection, whereby people with poor health find themselves occupying lower SES through decreased labour force participation or through withdrawal from paid work, thereby decreasing their wage income and inhibiting wealth accumulation [5,6]. Typically, authors examine the impact of childhood circumstances in adult SES, educational attainment and health by the use of multiple linear regression with a single outcome[7,8,9]. What makes our paper unique is that we model mid-life health, quality of life and cognitive function as a set of four inter-related multivariate outcomes.

Authors since the 1990s have advanced the importance of a life course approach to explore the relationship between early life circumstances and later life health outcomes[5,10]. This approach implies a reciprocal relationship between SES and health, and allows for the possibility that favourable circumstances in later life can compensate for the effects of earlier disadvantage [11]. At the same time, the theory of cumulative advantage/disadvantage suggests that individuals within a cohort increasingly diverge over the life course in terms of income, material resources and health, as large initial endowments lead to large additional gains and initial disadvantage causes further decline, regardless of the source of early advantage/disadvantage[12].

In attempting to unravel how accumulations and interactions of these various conditions affect health, some studies have shown that health variations in adults are attributed to both early and later life circumstances [2, 13], suggesting that childhood SES has lasting effects on health beyond its impact on adult SES. Others do not find a significant association between childhood SES and later health once adult SES is taken into account[14], suggesting that childhood SES typically determines the living and working conditions in adult life and it is these circumstances that give rise to social inequalities in health. From a life course perspective, SES mobility may influence health outcomes, with upward mobility decreasing the risk of ill-health and partially compensating for earlier

disadvantages, and downward mobility increasing its risk [11]. Others have emphasised the need for a fuller assessment of the interplay between material circumstances and psychosocial factors over the life course[15,16].

Researchers in the field of 'cognitive reserve'[17,18] have argued that education can itself lead to higher cognition in mid-life and early old age, through increased efficiency of neural network utilisation[19]. Thus it may be that parents with advantaged backgrounds are able to facilitate and promote social, cultural and educational advantage for their offspring which lead to better cognition, which is linked in turn with better health behaviours. We attempt in this paper to explore the pathways linking mid-life cognition with mid-life physical health.

More recently, interest has also focused on mental and emotional well-being in mid-life and early old age, indicating those with high levels of mental & emotional well-being have increased longevity[20], and that cognitive and socioemotional function can fuse to form skills supporting self-regulation, competence, and quality of life that persist into old age through linked reciprocal processes of genetic influence, nurturing, schooling, work, and lifestyle[21].

Our analysis builds upon the framework adopted by Wood et al[22] on the prediction of mental well-being using the Warwick-Edinburgh Mental Well-Being Scale[23] across four UK birth cohort studies, which showed that childhood SES is directly and indirectly (through adult socioeconomic pathways) linked to adult emotional well-being. We expand their approach by having a fuller set of outcome measures (albeit for a single cohort) which include physical and emotional well-being, together with quality of life. In addition, we include a cognitive performance measure at age 50 as our fourth outcome. Taken together, we examine the pathways from childhood to our multivariate mid-life outcome measures for men and women.

The 1958 British Birth Cohort Study (NCDS) provides a rich source of key aspects of the life course beginning with birth circumstances: birthweight, breastfeeding, maternal smoking during pregnancy and parental SES. In addition we include the effects of family difficulties such as unemployment, finance and housing in conjunction with childhood scores of socio-emotional adjustment and cognitive performance, each measured on three occasions spanning the ages 7, 11 and 16 years. The inclusion of SES at age 42 and lifetime highest qualification in the model enables us to trace which childhood effects persist and which are built upon or attenuated by education and social mobility.

Our conceptual framework (Fig.1) describes a 'pathways to risk model,' where the accumulation of advantage/disadvantage from childhood to mid-life represents a model of the life course where antecedent influences are postulated to have consequences for later life outcomes, notably the four life domains covering cognitive functioning, physical and emotional well-being and quality of life. These outcomes, along with those capturing cognitive performance at early ages and family difficulties, are defined as latent variables, each defined as a measurement model consisting of selected manifest variables represented by individual item responses (indicators) taken from NCDS survey data. The inter-relationships between the latent variables, and in particular the interplay between our outcomes, define a structural equation model (SEM) which underpins our statistical analysis[24].

For peer review only

Fig. 1 Conceptual model of Pathways from early childhood to mid-life PWB, EWB, QoL & Cognition

[insert Fig. 1 here]

DATA AND METHODS

Our data source, NCDS[25] provides operational measures regarding birth circumstances, the early and teen years covering ages 7, 11 and 16, and adulthood at ages 42 and 50 respectively. The analysis is based upon 3815 male and 4209 female cohort members present at each occasion who all have at least one genuine response for each of the four separate age 50 life domains, and who also participated in at least one of the three cognitive and socio-emotional assessments in order to permit a reliable strategy for handling missing items.

Our four age-50 life domains are represented by well-established measures: scales of physical and emotional well-being (hereafter PWB, EWB) are taken from the Medical Outcomes Survey (MOS) Short Form (SF-36) developed by RAND[26-29]. Quality of Life (hereafter QoL) is measured by the 12-point version of the 'Control, Autonomy, Self-Realisation and Pleasure' (CASPv2) QoL Scale[30]. The fourth domain outcome, cognitive ability (hereafter Age50 Cog) consists of four cognitive tests examining memory (word recall & delayed recall), executive function (animal naming), attention and mental speed (letter cancellation)[31].

Appendix 1 lists all of the manifest and latent variables employed in the analysis. Indicators for cognitive function at age 50 years are listed here, while those for physical, emotional and quality of life outcomes are expanded upon more fully in Appendix 2. Childhood behavioural indicators are listed in Appendix 3 following Rutter[32].

In fitting our structural equation models using MPlus[33] we obtain direct and indirect (mediated) effects of our background variables in terms of childhood social origins and social adjustment, on outcomes in early adulthood and mid-life at age 50 years.

The sets of predictors determining our paths differ slightly for each of the four outcomes so as to avoid the problem of 'seemingly unrelated regression'[34]

To address the issue of attrition bias caused by differential loss to follow-up in later sweeps, we employ full information maximum likelihood estimation (FIML)[35]. The SEM procedure that follows begins with an analysis of the sample as a whole, followed by separate analyses for men and women, supported by a multigroup analysis to test for model equivalence between men and women [36]. Model 'goodness of fit' is assessed via conventional criteria: 'root mean square error' (RMSEA), 'comparative fit index', (CFI) and 'Tucker Lewis fit index', (TLI) where RMSEA<0.05, CFI>0.90 and TFI> 0.90 would provide evidence of 'good fit'[37].

Patient and Public involvement statement

Participant consent was obtained from parents of cohort members at the initiation of the original birth cohort study in 1958. On attaining adulthood, permission was obtained from cohort members themselves at each subsequent longitudinal sweep.

For peer review only

RESULTS

The results begin with consideration of the inter-correlations between our mid-life outcome measures. Further univariate descriptives are contained in Appendix 1. Figure 2 contains a full SEM diagram for the sample as a whole. Subsequent reference will be made to Appendices 4 & 5 for the details of mediation, total and indirect effects and Appendix 6 contains further information on our multigroup analysis based on gender.

(a) Descriptives & correlations

Table 1 contains a correlation matrix of the four mid-life outcomes expressed as summative indices for each specific life domain.

Table 1. Associations between the four sets of mid-life outcomes as summative indices (Men/Women)

Age 50 Domain	Age 50 Cog	PWB	EWB	QoL
Cognitive Ability (Age 50 Cog)	1.00/1.00			
Physical Well-Being (PWB)	0.12/0.15	1.00/1.00		
Emotional Well-Being (EWB)	0.12/0.09	0.57/0.58	1.00/1.00	
Quality of Life (QoL)	0.15/0.10	0.48/0.48	0.63/0.64	1.00/1.00

Interestingly, QoL, PWB and EWB demonstrate moderate associations of inter-correlation. While cognitive ability is positively related to the physical and emotional well-being measures, the strength of the association is modest, with PWB having a higher coefficient for women and EWB & QoL a higher coefficient for men.

Fig. 2 contains estimates of association amongst these outcomes as defined by measurement models under a full SEM analysis.

(b) Birth SES effects on early mid-life outcomes

The path diagram shown in Fig 2 provides the model results for the sample as a whole showing only statistically significant paths at a p-level of .001, employing a Bonferroni correction for the fifty path coefficients being estimated (0.05/50=0.001) [38].

Fig 2. Estimated pathways of the Structural Equation Model, N=8024 (showing paths significant at p < .001)

[insert Fig.2 here]

Whilst there are various pathways that connect early life circumstances with cognitive performance, family difficulties and social adjustment there remains a small direct effect of social class at birth on each of our two early adult outcomes: Age 42 SES and Qualifications (both path coefficients equal

For peer review only

0.05). These two ‘interim’ or ‘pivotal’ destinations, in turn, have notable effects on our four mid-life outcomes at age 50, which we describe under (d) below.

(c) Indirect effects via pathways through childhood mediators

Inspecting the paths in Fig.2 from birth circumstances through ages 7, 11 and 16, we see firstly, that all four of our birth characteristics have direct effects on cognitive performance at age 7 years, when children are in primary school. Social class at birth has the most notable association with age 7 cognition (.27). This cognitive variable in turn has a strong direct effect on age 11 cognition (.92) and similarly for age 11 on cog performance at age 16 (.92).

There is a clear path via cognitive ability throughout childhood and adolescence, to adult achievement as indicated by Age 42 social class and educational qualifications (Age 16 Cog to SC 42yrs .50, Age 16 Cog to Quals .59).

This legacy of childhood cognitive performance is unsurprising, but interestingly Age 16 cognition also has a direct association with age 50 physical well-being (.12) besides more naturally, Age 50 cognition (.47).

Observed family difficulties in childhood, themselves associated with birth social class (-0.26), also play their part in understanding a child’s trajectory in cognitive performance. Characterised by issues with housing, finance and employment when a child is 7 years old, these family difficulties are seen to have negative consequences for cognitive performance at age 7 years (-.15) and a lagged negative effect on later performance age 16 years (-0.04).

Additional analyses also show that family difficulties impact on a child’s emotional and behavioural scores (based on Rutter) at ages 7, 11 and 16 (.09, .06 and .10 respectively). In turn these scores had small significant negative associations with cognitive performance at each corresponding age (-.13, -.04 and -.05 respectively). But most notably, Rutter scores at these three ages are significantly correlated (.49 age 7 to 11 and .44 age 11 to 16), and there are direct effects from age 16 Rutter scores to three of our four age 50 outcomes: EWB (-0.13), QoL (-0.10) and PWB (-0.07).

(d) Indirect effects via adulthood mediators

Adult social class and lifetime qualifications are, together, influential predictors of each of our four mid-life outcomes: adult social class has significant predictive effects (.08 and .11) on PWB and QoL, and our Bonferroni-corrected criterion of $p < .001$ narrowly excludes its link to EWB (.05 at $p < .004$).

In general, the socially-advantaged enjoy better health and report higher levels of quality of life than their less advantaged counterparts. Interestingly, educational qualifications predict EWB (.06) and QoL (.10) but not PWB (.04 at $p < .01$, so not quite significant). As one would expect, educational qualifications are significantly linked to age 50 cognitive ability (.05), EWB (.06) and QoL (.10).

(e) Gender differences

Our multigroup analysis reveals that certain paths have significantly different effects for men and women. In Appendix 5 we display two separate path diagrams for men and women. For men, the direct effect of social class at birth on social class and qualifications in early adulthood is notably stronger (.09 and .07 respectively) than for women (.05, .05).

For peer review only

Both sexes see the birth social class effect remain equally strong for cognitive performance at age 7 years and beyond, but women experience a slightly worse negative effect of social disadvantage at birth on the latent variable 'family difficulties' (-0.27 compared to -0.24). In turn, family difficulties have slightly more influence on Age 7 cognition (.17) for men than for women (.14), and a small lagged effect on Age 16 cognition only evident for women. After age 7 the Rutter social adjustment scores do not appear to impact upon the cognitive performance scores for men, but they do for women at 11 and 16, though the effect is small (.05).

Early adult social class has a slightly stronger influence for men than women on our age 50 outcomes PWB and QoL, and Qualifications have a significant effect on EWB for men but not for women. In contrast, women's age 16 cognition has a significant effect on their later PWB (.17), an effect not significant for men; and the path from age 16 cognition to age 50 Cog has a stronger coefficient for women (.47) than men (.43).

Pursuing the evidence for gender differences via a sensitivity analysis under MPlus which formally tests for statistical difference between paths for each sex. Appendix 6 identifies six paths where the effects for men and women are significantly different, but only three have notable differences in terms of their effect sizes in the multigroup analysis. These are:

- stronger effect for women from age 16 cognition to age 50 cognition as noted above (.47 to .43);
- direct effect of social class at birth on early social class (much stronger for men); and
- small effect of age 42 social class on Age 50 Cognition (.07 at p<.002 for men, no sig effect women)

DISCUSSION

Structural equation modelling has teased out the nuances of the influence of pathways from childhood and early adulthood to our four related outcomes of health, well-being and cognition in mid-life. The work demonstrates that a holistic approach to defining the aspects of life that matter in mid-life can be revealing. Physical and emotional health matter for a person's subjective account of their quality of life, and PWB correlates at .69 with EWB, which in turn correlates .83 with QoL. While cognitive performance at age 50 is relatively weakly related, the other three are closely intertwined. These associations are similar in magnitude to those reported in table 1; however they are now estimated by taking the legacy of the life course into account.

We also show that some of the paths to health and well-being in mid-life are different for men and women[39]. However, important communalities remain.

Social origins, in particular social class at birth, have a major indirect influence on mid-life outcomes, the mediation process beginning with a child's cognitive performance throughout formal schooling and subsequently via cognitive performance at age 16 years. Collectively, these influences are weakened or reinforced by the existence of family difficulties in early childhood (themselves influenced by childhood social class). Whilst cognitive performance is a strong predictor of early adult social class and educational status for both men and women, social class at birth also has a lasting association with these mid-life indicators: more so for men than for women.

For peer review only - http://bmjopen.bmj.com/site/about/guidelines.xhtml

This could well suggest that women have a looser connection with the legacy of social class at birth, perhaps partly because their path to advantaged SES at age 42 may be impeded by caring responsibilities and gender inequalities in the labour market[40].

Early adult social class influences all four of our mid-life outcomes, but we see the direct effect of social class at birth on social class and qualifications in early adulthood is notably stronger for men (.09 and .07 respectively) than for women (.05, .05).

A relatively small but consistent direct effect of qualifications on Age 50 Cognition supports those who argue that education has a causal effect on subsequent cognitive functioning[41], (but note alternative views on this[42]). Equally, the relationship between QoL and early adult social class supports the work of Blane et al[43].

The strength in the evidence presented here lies in the richness of its longitudinal dimension which conveys the importance of early life circumstances upon early adult and mid-life measures of achievement, health and well-being. It may be premature to identify the impact of any early cognitive decline; however, cognitive performance in the teen years does hold a legacy for cognitive performance in mid-life.

Social class at birth has a strong influence on early cognitive performance and beyond, which in turn has consequences for adult achievement and social class. Clearly, the presence of family difficulties and behavioural issues will impede (mediate) this trajectory. Interestingly, childhood behaviour, in particular at age 16, has a lasting effect on mid-life EWB and Quality of Life.

Health, emotion, quality of life and to a lesser extent cognitive function do not stand alone as markers of well-being in mid-life. They represent the constituents of an inter-related whole which is shaped by early life circumstances, family difficulties, social adjustment and cognitive performance in childhood.

Acknowledgements

Assistance from ESRC grants ES/N003683/1 and ES/M008584/1.

Data Sharing statement

The analysis is based on secondary data available for download from the UK Data Archive. Syntax used to derive variables for this analysis can be obtained by e-mailing the corresponding author, Brian Dodgeon.

Ethics, funding, data sharing

Ethical approval was covered by the codes of practice of the funding agency

(ESRC grants ES/N003683/1 and ES/M008584/1 quoted above).

References

1 Feinstein, J. S. (1993). The relationship between socioeconomic status and health: A review of literature. Milbank Quarterly 71(2), 279–322.

For peer review only

2 Gilman, S. E., Kawachi, I., Fitzmaurice, G. M. et al (2002). Socioeconomic status in childhood and the lifetime risk of major depression. International Journal of Epidemiology 31(2), 359–367.

3 Wilkinson, R and Marmot, M, eds. (2003). Social Determinants of Health: The Solid Facts (2nd ed). World Health Organization Europe. ISBN 978-92-890-1371-0.

4 Marmot, M.G and Siegrist, J (2006) Social Inequalities In Health: New Evidence And Policy Implications, Oxford University Press.

5 Williams, D.R. (1990). Socioeconomic differentials in health: A review and redirection. Social Psychology Quarterly 53, 81–99.

6 Blane, D, Smith, G.D and Bartley, M (1993) Social selection: what does it contribute to social class differences in health? Sociology of Health & Illness, 15(1), 1-15

7 Lundberg, O. (1991). Childhood living conditions, health status, and social mobility: A contribution to the health selection debate. European Sociological Review 7(2), 149–162

8 Blackwell, D. L., Hayward, M. D., & Crimmins, E. M. (2001). Does childhood health affect chronic morbidity in later life? Social Science & Medicine 52(8), 1269–1284

9 Power, C, Stansfeld, S. A, Matthews et al (2002) Childhood and adulthood risk factors for socio-economic differentials in psychological distress: evidence from the 1958 British birth cohort. ***Social Science and Medicine*** 55(11), 1989.

10 Wadsworth, M. E. J. (1997). Health inequalities in the life course perspective. Social Science & Medicine 44, 859–869.

11 Graham, H. (2002). Building an inter-disciplinary science of health inequalities: The example of lifecourse research. Social Science & Medicine 55(11), 2005–2016.

12 Dannefer, D (2003) Cumulative advantage/disadvantage and the life course: Cross-fertilizing age and social science theory. The Journals of Gerontology, Series B, 58(6), S327-S337.

13 Ploubidis, G.B, Benova, L., Grundy, E. et al (2014): Lifelong Socio Economic Position and biomarkers of later life health: Testing the contribution of competing hypotheses. Social Science and Medicine 119, 258-265.

14 Marmot, M, Shipley, M, Brunner, E et al (2001) Relative contribution of early life and adult socioeconomic factors to adult morbidity in the Whitehall II study. Journal of Epidemiology & Community Health 55(5), 301-307.

15 Lynch, J.W, Davey Smith, G, Kaplan et al (2000) Income inequality and mortality: importance to health of individual income, psychosocial environment, or material conditions. British Medical Journal 320(7243), 1200.

16 Turrell, G, Hewitt, B, Patterson et al (2002) Socioeconomic differences in food purchasing behaviour and suggested implications for diet-related health promotion. Journal of Human Nutrition and Dietetics 15(5), 355-364.

For peer review only

17 Richards, M and Sacker, A (2003) Lifetime Antecedents of Cognitive Reserve. Journal of Clinical and Experimental Neuropsychology 25 (5), 614-624.

18 Staff, R.T, Murray, A.D, Deary, I.J et al (2004) What provides cerebral reserve? Brain 127 (5), 1191-1199.

19 Richards, M and Deary, I.J (2005) A life course approach to cognitive reserve: a model for cognitive aging and development? Annals of Neurology 58(4), 617-622.

20 Steptoe, A, Deaton, A and Stone, A.A (2015) Subjective wellbeing, health and ageing. Lancet 385 (9968): 640-648.

21 Richards, M and Hatch, S.L (2011) A Life Course Approach to the Development of Mental Skills. The Journals of Gerontology Series B 66b (Suppl.1), i26-i35.

22 Wood, N, Bann, D, Hardy et al (2017) Childhood socioeconomic position and adult mental wellbeing: evidence from British birth cohort studies. PLoS One, 10, e0185798.

23 Tennant, R, Hiller, L, Fishwick, R et al (2007) The Warwick-Edinburgh Mental Well-Bing Scale (WEMWBS): development and UK validation. Health and Quality of Life Outcomes 5(1), 63.

24 Kline, R.B. (1998). *Principles and Practice of structural equation modelling*. New York. Guildford Press.

25 Power, C and Elliott, J. (2006) Cohort Profile: 1958 British birth cohort (National Child Development Study). International Journal of Epidemiology 35(1), 34-41.

26 Ware, J.E and Donald Sherbourne, C (1992). The MOS 36-Item Short-Form Health Survey (SF-36): I. Conceptual Framework and Item Selection. Medical Care 30(6), 473-483.

27 Brazier, J.E, Harper, R, Jones et al (1992). Validating the SF-36 health survey questionnaire: new outcome measure for primary care. British Medical Journal 305(6846): 160-164.

28 Jenkinson, C., Wright, L. and Coulter, A. (1994). Criterion validity and reliability of the SF-36 in a population sample. Quality of Life Research 3(1), 7-12.

29 Burnholt, V & Nash, P. (2011). Short Form 36 (SF-36) Health Survey Questionnaire: normative data for Wales. Journal of Public Health, 33(4), 587-683.

30 Wiggins, R.D, Netuveli, G., Hyde et al (2008). The evaluation of a self-enumerated scale of quality of life (CASP-19) in the context of research on ageing: a combination of exploratory and confirmatory approaches. Social Indicators Research 89(1), 61-77.

31 Brown, M and Dodgeon, B. (2010) NCDS Cognitive Assessments at Age 50: Initial Results. CLS Working Paper 2010/1. London: Centre for Longitudinal Studies. UCL.

32 Rutter, M, Tizard, J and Whitmore, K (1970) Education, Health & Behaviour, Longman.

33 Muthen, B.O & Muthen L.K (2018) Statistical Analysis with Latent Variables: MPlus v8.1 User Manual. Los Angeles CA: Muthen & Muthen.

For peer review only

34 Zellner, A. 1962. An efficient method of estimating seemingly unrelated regression equations and tests for aggregation bias. Journal of the American Statistical Association 57: 348–368.

35 Arbuckle, J.L. (1996). Full Information estimation in the presence of incomplete data. In Marcoulides, G.A & & Schumaker, R.E (eds) *Advanced Structural Equation Modeling: Issues and Techniques.* Mahwah, N.J.: Erlbaum Associates.

36 Byrne, B.M. (2012). *Structural Equation Modeling with Mplus*. Routledge Taylor & Francis Group. New York & London

37 Bollen, K.A. & Scott Long, J. (eds) (1993). *Testing Structural Equation Models*. SAGE Focus edition. Newbury Park, London & New Delhi.

38 Weisstein, Eric W (2004) "Bonferroni Correction." From MathWorld--A Wolfram Web Resource. http://mathworld.wolfram.com/BonferroniCorrection.html

39 Arber, S. & Cooper, H. (1999). Gender differences in health in later life: the new paradox? Social Science and Medicine, 48(1), 61-76

40 Kahn, J.R, Garcia-Manglano, J and Bianchi, S.M (2014) The Motherhood Penalty at Midlife: Long-Term Effects of Children on Women's Careers. Journal of Marriage and the Family 76(1), 56-72.

41 Richards, M and Sacker, A (2010) Is education causal? Yes. International Journal of Epidemiology 40(2), 516–518.

42 Deary, I.J and Johnson, W (2010) Intelligence and education: causal perceptions drive analytic processes and therefore conclusions. International Journal of Epidemiology 39(5), 1362-1369.

43 Blane, D, Netuveli, G and Bartley, M (2007). Does quality of life at older ages vary with socio-economic position? Sociology **41**(4), 717-726.

For peer review only

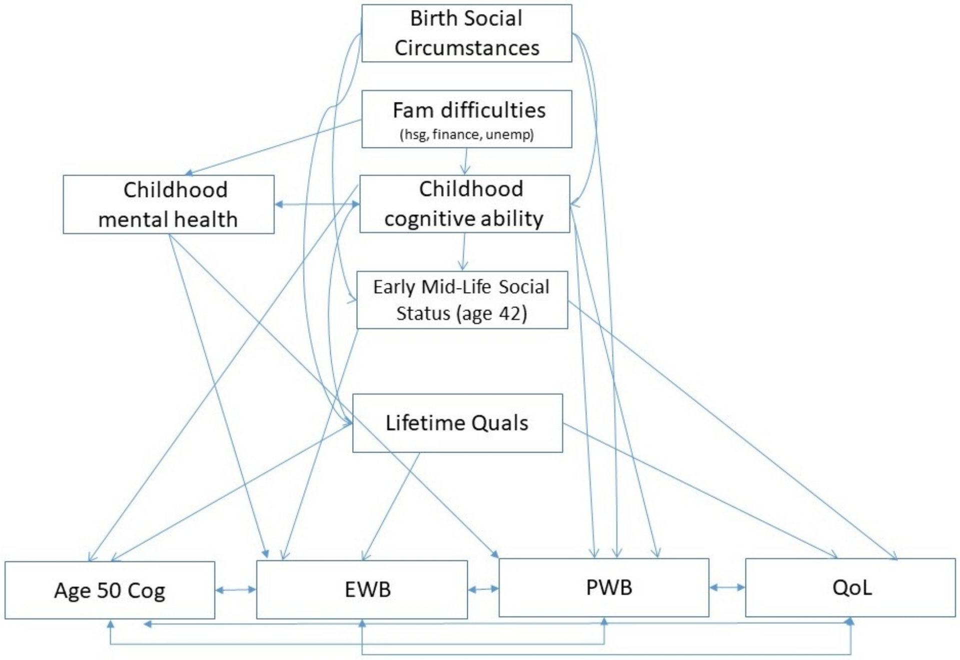

Fig. 1 Conceptual model of Pathways from early childhood to mid-life PWB, EWB, QoL & Cognition

258x174mm (96 x 96 DPI)

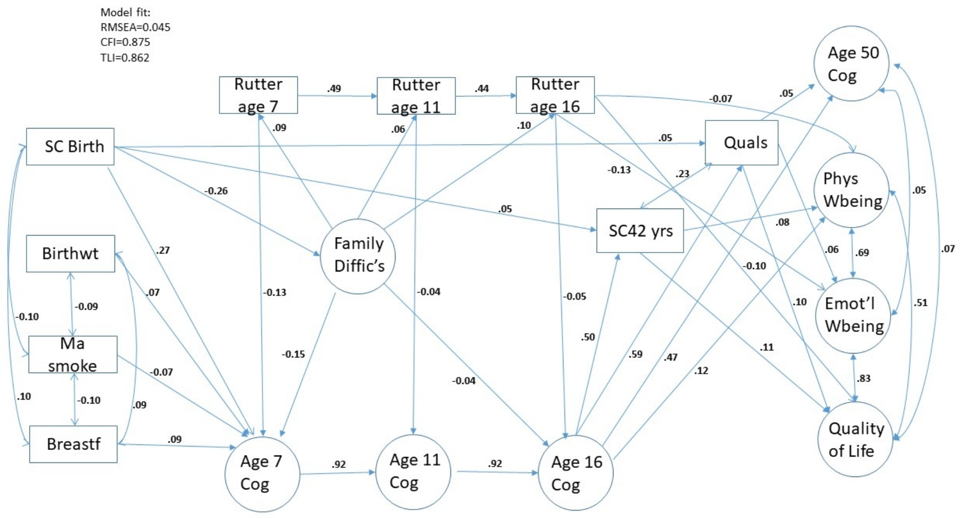

Fig 2. Estimated pathways of the Structural Equation Model, N=8024 (showing paths significant at p < .001)

338x190mm (96 x 96 DPI)

Appendix 1: Description of manifest variables, age CM at data collection; latent variable labelling

Variable	NCDS Sweep	Coding	Mean	St.Dev	% missing	Latent Variable
Early years						
Social class of father (GRO 1951)	Birth	1-5 (1=low)	2.98	0.92	6.7	
Birthweight	Birth	1 <2.5 kg<br>2 >=2.5kg	1.94	0.23	2.8	
Did mother smoke after 4th month of pregnancy	Birth	1-4(No/Variable Med'm/Heavy)	1.7	1.10	3.6	
Sex	Birth	1,2	52.5% female		0	
Mother breastfed child	Age 7	1 No; 2 <1 mth;<br>3 >1 month	2.15	0.86	10.4	
Problem arithmetic score	Age 7	0-10	5.38	2.43	9.1	
Copying designs test age 7	Age 7	0-12	7.19	1.93	9.1	Age7cog
Draw-A-Man test score	Age 7	0-51	24.40	6.95	10.6	
Southgate reading test score	Age 7	0-30	24.43	6.36	8.9	
Fam difficulties by age 7:<br>Housing (health visitor assess't)	Age 7	0 No; 1 D/K<br>2Yes	0.17	0.51	9.9	
Fam difficulties by age 7: Finance	Age 7	0,1,2	0.21	0.53	10.0	Famdifs
Fam difficulties by age 7: Unempl	Age 7	0,1,2	0.11	0.38	10.0	
NFER general ability test	Age 11	0-80	46.14	15.2	12.0	
Reading Comprehension score	Age 11	0-40	16.97	5.95	12.0	Age11cog
Maths score	Age 11	0-40	18.40	10.1	12.0	
Copying designs test age 11	Age 11	0-12	8.45	1.40	12.2	
Rutter behavioural score (age 7)<br>(mother assess't at each age)	Age 7	0-28	6.13	3.50	9.7	
Rutter behavioural scor (age 11)	Age 11	0-28	6.13	3.41	12.8	

For peer review only

Rutter behavioural score (age 16	Age 16	0-36	3.91	3.42	24.4	
Adolescence						
Reading Comprehension age 16	Age 16	0-40	26.58	6.23	23.1	Age16cog
Maths test age 16	Age 16	0-40	13.83	6.96	23.4	
Teacher assess't English ability	Age 16	1-5	3.44	1.12	22.9	
Teacher assess't Maths ability	Age 16	1-5	3.11	1.19	23.5	
Early Mid-Life						
Social class at age 42	Age 42	1-6 (1=low)	3.99	1.24	13.9	
Later Mid-Life						
Highest qual'n (acad or vocat) achieved by age 50	Age 50	0-5 (0=no quals; 1-5=NVQ)	2.68	1.36	0	
Latter cancellation speed	Age 50	0-800	334.60	87.8	3.5	Age50cog
Word recall	Age 50	0-10	6.60	1.46	2.1	
Delayed word recall	Age 50	0-10	5.49	1.81	2.7	
Animal naming test	Age 50	0-65	22.53	6.22	2.1	
SF-36 phys. health (4 scores: see Appx.1. Summative stats shown)	Age 50	0-100 (each of 4 scores)	315.4	83.8	0.2	Physhlth
SF-36 mental health (4 scores)	Age 50	0-100 (4 scores)	276.6	63.3	0.3	Emot'l Wbeing
CASP-12 (3 control scores, 3 autonomy, 3 pleasure, 3 self-realisation – see Appx.1	Age 50	0,1,2,3 (each of 12 scores)	23.94	5.42	0.7	CASP12

For peer review only

Appendix 2. Wording of SF-36 scale items and CASP-12 (v2) items

SF-36 Item	Wording
1 Self-assessed General Health<br><br>*(Domain in our model: Phys Health)*	In general, would you say your health is:<br><br>(1) Excellent; (2) Very good; (3) Good;<br>(4) Fair; (5) Poor
2 Self-assessed General Health compared to one year ago<br><br>*(Domain: Phys Health)*	2. Compared to one year ago, how would you rate your health in general now?<br><br>(1) Much better now than one year ago;<br><br>(2) Somewhat better now than 1 year ago;<br><br>(3) About the same;<br><br>(4) Somewhat worse now than 1 year ago;<br><br>(5) Much worse now than one year ago
Health now limiting specific physical activities:<br><br>3. Vigorous activities?<br><br>*(Domain: Phys Health)*<br><br>4. Moderate activities?<br><br>*(Domain: Phys Health)*<br><br>5. Lifting or carrying groceries?<br><br>*(Domain: Phys Health)*<br><br>6. Climbing several flights of stairs?<br><br>*(Domain: Phys Health)*<br><br>7. Climbing one flight of stairs?	The following items are about activities you might do during a typical day. Does your health now limit you in these activities? If so, how much?<br><br>3 Vigorous activities, such as running, lifting heavy objects, participating in strenuous sports<br><br>(1) Yes, limited a lot;<br><br>(2) Yes, limited a little;<br><br>(3) No, not limited at all<br><br>4. Moderate activities, such as moving a table, pushing a vacuum cleaner, bowling, or playing golf?<br><br>Answer (1)(2)(3)<br><br>5. Lifting or carrying groceries?<br><br>Answer (1)(2)(3)<br><br>6. Climbing several flights of stairs?<br><br>Answer (1)(2)(3)<br><br>7. Climbing one flight of stairs?<br><br>Answer (1)(2)(3)

For peer review only

(Domain: Phys Health)<br><br>8. Bending, kneeling, or stooping?<br><br>*(Domain: Phys Health)*<br><br>9. Walking more than a mile?<br><br>*(Domain: Phys Health)*<br><br>10. Walking several blocks?<br><br>*(Domain: Phys Health)*<br><br>11. Walking one block?<br><br>*(Domain: Phys Health)*<br><br>12. Bathing or dressing yourself?<br><br>*(Domain: Phys Health)*	8. Bending, kneeling, or stooping?<br><br>Answer (1)(2)(3)<br><br>9. Walking more than a mile?<br><br>Answer (1)(2)(3)<br><br>10. Walking several blocks?<br><br>Answer (1)(2)(3)<br><br>11. Walking one block?<br><br>Answer (1)(2)(3)<br><br>12. Bathing or dressing yourself?<br><br>Answer (1)(2)(3)
During the past 4 weeks, have you had any of the following problems with your work or other regular daily activities as a result of your physical health?<br><br>13. Cut down the amount of time you spent on work or other activities?<br><br>*(Domain: Phys Health)*<br><br>14. Accomplished less than you would like<br><br>*(Domain: Phys Health)*<br><br>15. Were limited in the kind of work or other activities<br><br>*(Domain: Phys Health)*<br><br>16. Had difficulty performing the work or	13. Cut down the amount of time you spent on work or other activities?<br><br>(1) Yes (2) No<br><br>14. Accomplished less than you would like?<br><br>(1) Yes (2) No<br><br>15. Were limited in the kind of work or other activities<br><br>(1) Yes (2) No

For peer review only

other activities<br><br>*(Domain: Phys Health)*	16. Had difficulty performing the work or other activities (for example, it took extra effort)<br><br>(1) Yes (2) No
During the past 4 weeks, have you had any of the following problems with your work or other regular daily activities as a result of any emotional problems (such as feeling depressed or anxious)?<br><br>17. Cut down the amount of time you spent on work or other activities<br><br>*(Domain: Emotional WBeing)*<br><br>18. Accomplished less than you would like<br><br>*(Domain: Emotional WBeing)*<br><br>19. Didn't do work or other activities as carefully as usual<br><br>*(Domain: Emotional WBeing)*	17. Cut down the amount of time you spent on work or other activities?<br><br>(1) Yes (2) No<br><br>18. Accomplished less than you would like?<br><br>(1) Yes (2) No<br><br>19. Didn't do work or other activities as carefully as usual?<br><br>(1) Yes (2) No
20. During the past 4 weeks, to what extent has your physical health or emotional problems interfered with your normal social activities with family, friends, neighbours, or groups?<br><br>*(Domain: Emotional WBeing)*	20. During the past 4 weeks, to what extent has your physical health or emotional problems interfered with your normal social activities with family, friends, neighbours, or groups?<br><br>(1) Not at all;<br><br>(2) Slightly;<br><br>(3) Moderately;<br><br>(4) Quite a bit;<br><br>(5) Extremely
21. How much bodily pain have you had during the past 4 weeks?<br><br>*(Domain: Phys Health)*	21. How much bodily pain have you had during the past 4 weeks?<br><br>(1) None;

For peer review only

	(2) Very mild;<br>(3) Mild;<br>(4) Moderate;<br>(5) Severe;<br>(6) Very severe
22. During the past 4 weeks, how much did pain interfere with your normal work (including both work outside the home and housework)?<br>*(Domain: Phys Health)*	22. During the past 4 weeks, how much did pain interfere with your normal work (including both work outside the home and housework)?<br>(1) Not at all;<br>(2) A little bit;<br>(3) Moderately;<br>(4) Quite a bit;<br>(5) Extremely
These questions are about how you feel and how things have been with you during the past 4 weeks. For each question, please give the one answer that comes closest to the way you have been feeling.<br>How much of the time during the past 4 weeks...<br>23. Did you feel full of pep?<br>*(Domain: Emotional WBeing)*<br>24. Have you been a very nervous person?<br>*(Domain: Emotional WBeing)*	How much of the time during the past 4 weeks...<br>23. Did you feel full of pep?<br>(1) All of the time;<br>(2) Most of the time;<br>(3) A good bit of the time;<br>(4) Some of the time;<br>(5) A little of the time;<br>(6) None of the time

For peer review only

25. Have you felt so down in the dumps that nothing could cheer you up?<br><br>*(Domain: Emotional WBeing)*<br><br>26. Have you felt calm and peaceful?<br><br>*(Domain: Emotional WBeing)*<br><br>27. Did you have a lot of energy?<br><br>*(Domain: Emotional WBeing)*<br><br>28. Have you felt downhearted and blue?<br><br>*(Domain: Emotional WBeing)*<br><br>29. Did you feel worn out?<br><br>*(Domain: Emotional WBeing)*<br><br>30. Have you been a happy person?<br><br>*(Domain: Emotional WBeing)*<br><br>31. Did you feel tired?<br><br>*(Domain: Emotional WBeing)*	24. Have you been a very nervous person?<br><br>Answer (1)-(6)<br><br>25. Have you felt so down in the dumps that nothing could cheer you up?<br><br>Answer (1)-(6)<br><br>26. Have you felt calm and peaceful?<br><br>Answer (1)-(6)<br><br>27. Did you have a lot of energy?<br><br>Answer (1)-(6)<br><br>28. Have you felt downhearted and blue?<br><br>Answer (1)-(6)<br><br>29. Did you feel worn out?<br><br>Answer (1)-(6)<br><br>30. Have you been a happy person?<br><br>Answer (1)-(6)<br><br>31. Did you feel tired?<br><br>Answer (1)-(6)
32. During the past 4 weeks, how much of the time has your physical health or emotional problems interfered with your social activities (like visiting with friends, relatives, etc.)?<br><br>*(Domain: Emotional WBeing)*	32. During the past 4 weeks, how much of the time has your physical health or emotional problems interfered with your social activities (like visiting with friends, relatives, etc.)?<br><br>(1) All of the time;<br><br>(2) Most of the time;<br><br>(3) Some of the time;<br><br>(4) A little of the time;<br><br>(5) None of the time
How TRUE or FALSE is each of the following statements for you.<br><br>33. I seem to get sick a little easier than	How TRUE or FALSE is each of the following statements for you.<br><br>33. I seem to get sick a little easier than other people

For peer review only

other people<br><br>*(Domain: Phys Health)*	(1) Definitely true;<br><br>(2) Mostly true;<br><br>(3) Don't know;<br><br>(4) Mostly false;<br><br>(5) Definitely false
34. I am as healthy as anybody I know<br><br>*(Domain: Phys Health)*	34. I am as healthy as anybody I know<br><br>Answer (1)-(5)
35. I expect my health to get worse<br><br>*(Domain: Phys Health)*	35. I expect my health to get worse<br><br>Answer (1)-(5)
36. My health is excellent<br><br>*(Domain: Phys Health)*	36. My health is excellent<br><br>Answer (1)-(5)

Note that the answers to the 36 questions were recoded on a scale from 0 to 100 with 100 indicating the highest levels of health (i.e. negatively-phrased questions being coded in reverse polarity).

These were then divided into eight sub-domains (four physical health, four emotional well-being, with the recoded variables being summed then divided by the number of questions asked:

Domain	Physical Health		Emotional Well-Being	
Sub-domain	Physical functioning	Pain	Role limitations due to emotional problems	Fatigue
	Role limitations due to physical health	General Health	Emotional Well-Being	Social Functioning

For peer review only

CASP-12 (v2) Item	Wording
1-3 Control	1 My age prevents me from doing the things I would like to.<br>(1) Often; (2) Sometimes; (3) Not often; (4) Never<br>2 I feel what happens to me is out of my control.<br>Answer (1)-(4)<br>3 I feel left out of things<br>Answer (1)-(4)
4-6 Autonomy	4 I can do the things I want to do.<br>Answer (1)-(4)<br>5 Family responsibilities prevent me from doing what want to do.<br>Answer (1)-(4)<br>6 I feel that I can please myself what I do.<br>Answer (1)-(4)<br>6? Shortage of money stops CM doing things wants to do.<br>Answer (1)-(4)
7-9 Self-Realisation	7 I look forward to each day.<br>Answer (1)-(4)<br>8 I feel that my life has meaning.<br>Answer (1)-(4)<br>9 I enjoy the things that I do.<br>Answer (1)-(4)<br>9? On balance I look back on life with sense of happiness.<br>Answer (1)-(4)
10-12 Pleasure	10 I feel full of energy these days.

For peer review only

	Answer (1)-(4)<br><br>11 I feel that life is full of opportunities.<br><br>Answer (1)-(4)<br><br>12 I feel that the future looks good for me.<br><br>Answer (1)-(4)

For peer review only

Appendix 3 Rutter Behaviour scale, NCDS aged 16 (18-point version asked to mother)

Below are a series of descriptions of behaviour often shown by young people. Please ask the mother about each one and ring the appropriate number to show the degree to which this description is true of the study child:

0 Does not apply
1 Applies somewhat
2 Certainly applies

1	Restless, difficulty staying seated
2	Squirmy, fidgety child
3	Often destroys others property
4	Frequently fights, quarrelsome
5	Not much liked by other children
6	Often worries about things
7	Does things on own, rather solitary
8	Irritable, flies off the handle
9	Appears miserable, unhappy & tearful
10	Twitches,mannerisms,tics-face or body
11	Frequently sucks thumb or fingers
12	Frequently bites nails or fingers
13	Is often disobedient
14	Cannot settle more than a few moments
15	Fearful of new situations or things
16	Fussy, over particular
17	Often tells lies
18	Bullies other children

For peer review only

Appendix 4: Calculation of Indirect Effect and Total Effects in the Structural Equation Model

A Indirect effects from early-life circumstances to Age 50 outcomes (where total effect size >.01 and statistically significant)

Effects from	To	Total effect	Total indirect	Sig. direct effect? (p<.001)	Total effect significant?
SC Birth	Age 50 Cog	0.117	0.117	No	Yes
Birthwt	Age 50 Cog	0.047	0.030	No	Yes
Rutter7	Age 50 Cog	-0.065	-0.071	No	Yes
Rutter11	Age 50 Cog	-0.038	-0.025	No	Yes
Rutter16	Age 50 Cog	-0.018	-0.023	No	Yes
Masmoke	Age 50 Cog	-0.027	-0.027	No	Yes
Breastf	Age 50 Cog	0.039	0.039	No	Yes
SC Birth	PWB	0.081	0.048	No	Yes
Birthwt	PWB	0.011	0.011	No	Yes
Breastf	PWB	0.014	0.014	No	Yes
Rutter7	PWB	-0.043	-0.059	No	Yes
Rutter11	PWB	-0.081	-0.039	No	Yes
Rutter16	PWB	-0.074	-0.002	Yes	Yes
Masmoke	PWB	-0.010	-0.010	No	Yes
Rutter7	EWB	-0.046	-0.046	No	Yes
Rutter11	EWB	-0.080	-0.061	No	Yes
Rutter16	EWB	-0.133	-0.003	Yes	Yes
SC Birth	QoL	0.055	0.033	No	Yes
Rutter7	QoL	-0.048	-0.034	No	Yes
Rutter11	QoL	-0.049	-0.049	No	Yes
Rutter16	QoL	-0.103	-0.004	Yes	Yes

For peer review only

B Indirect effects from early-life circumstances to early adult outcomes (total effect size >.01 and statistically significant)

Effects from	To	Total effect	Total indirect	Sig. direct effect? (p<.001)	Total effect significant?
SC Birth	SC42yrs	0.167	0.113	Yes	Yes
SC Birth	Quals	0.184	0.135	Yes	Yes
Birthwt	SC42yrs	0.030	0.030	No	Yes
Birthwt	Quals	0.035	0.035	No	Yes
Masmoke	SC42yrs	-0.027	-0.027	No	Yes
Masmoke	Quals	-0.032	-0.032	No	Yes
Breastf	SC42yrs	0.039	0.039	No	Yes
Breastf	Quals	0.047	0.047	No	Yes

For peer review only

C Indirect effects from early life circumstances to Age 50 outcomes (total effect size <.01 or not statistically significant)

Effects from	to	Total effect	Total indirect	Sig. direct effect? (p<.001)	Total effect significant?
SC Birth	EWB	0.031	0.012	No	No
Birthwt	EWB	0.007	0.004	No	No
Masmoke	EWB	-0.003	-0.003	No	Yes
Breastf	EWB	0.005	0.005	No	Yes
Birthwt	QoL	0.003	0.006	No	No
Masmoke	QoL	-0.005	-0.005	No	Yes
Breastf	QoL	0.008	0.008	No	Yes

Commentary: Three direct influences stand out in Table A, each involving the Rutter scores at age 16 years (see Appx 3).

These are modest direct influences on PWB and QoL, but a relatively stronger negative influence on EWB at age 50 years.

Two direct influences stand out in Table B: from social class at birth to social class at 42 years and Qualifications obtained. The direct influences are shown in our estimated pathways diagram (Fig.2), but we also see in Table C there is an additional indirect effect (via childhood cognition at ages 7/11/16), over twice as large as the direct effect in both cases.

For peer review only

Appendix 5: Separate analyses by sex

Running the analyses separately by sex, we see that certain pathways are significant for one sex but not the other (p< .001), and certain path coefficients are notably different:

Men only (N=3815)

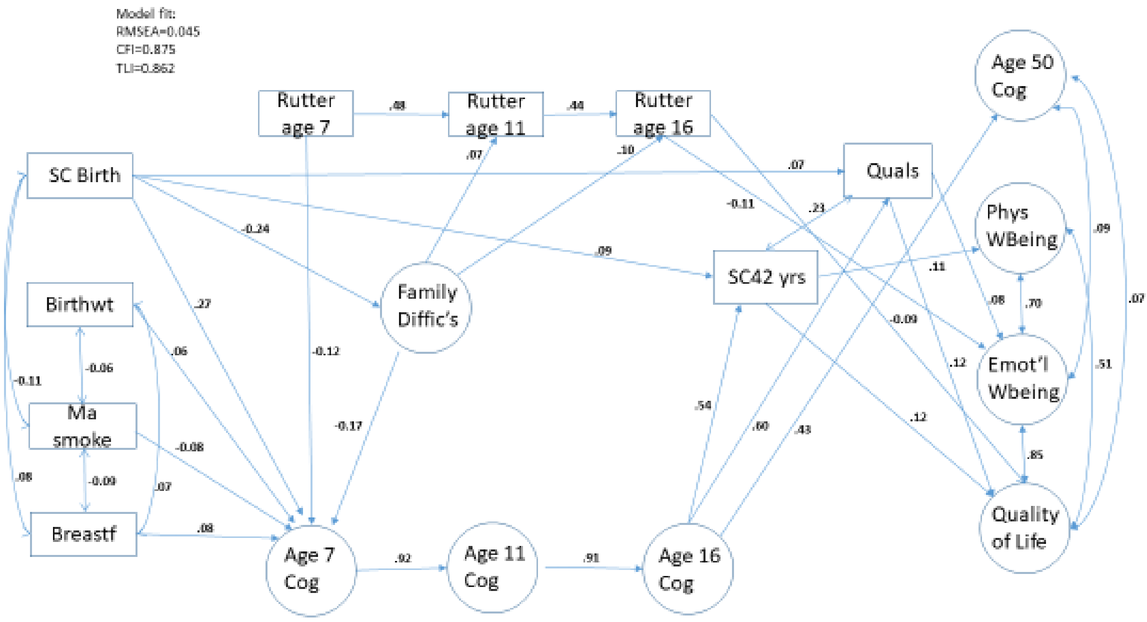

Women only (N=4209)

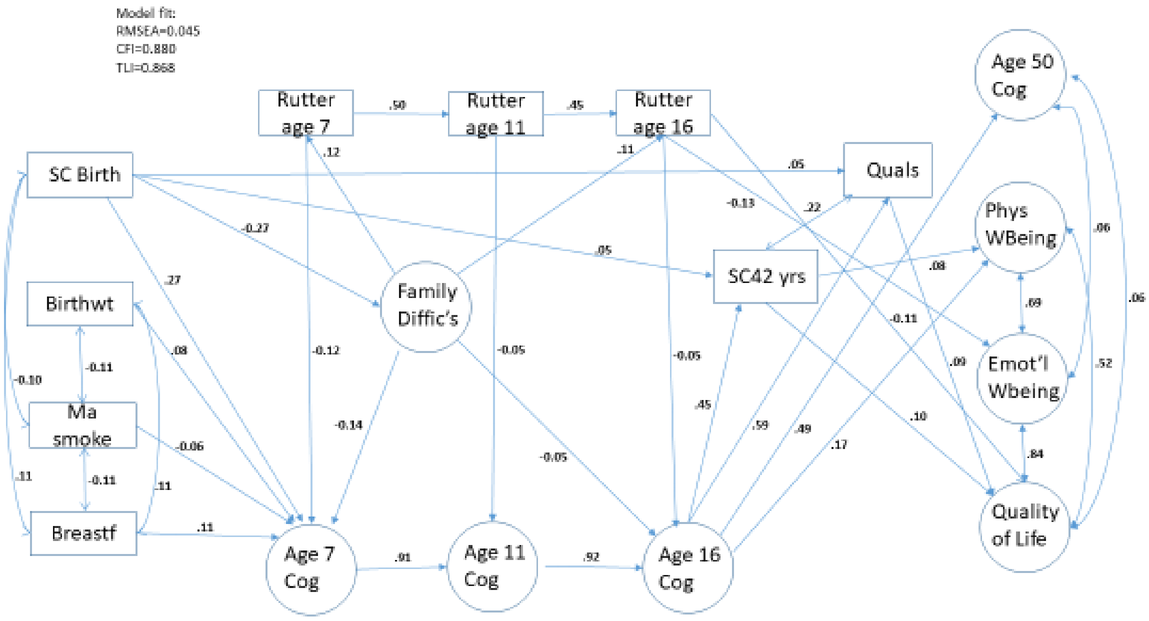

Appendix 6: Sensitivity analysis (multigroup by sex)

An MPlus multigroup run by sex was performed as a sensitivity analysis, in which each of the 50 possible paths in Fig.2 was labelled m1-m50, and (in the female model) f1-f49, then a set of 50 ‘difference’ variables was defined diff(i)=m(i)-f(i).

Testing the significance of these 50 ‘diff’ variables, 6 were found to be significant, and the other 43 non-significant.

The MPlus multigroup model was then re-run, constraining all the 44 non-significant paths to be equal for males and females, but allowing the other six paths to vary, thus increasing the parsimony of the model.

The six paths found to be significantly different between men and women are shown in the table below:

	Men: Estimate	P-val	Women: Estimate	P-val
Age 50 cog on SC 42 yrs	0.07	0.002	-0.03	0.146
Age 50 cog on Age 16 Cog	0.42	0.000	0.47	0.000
SC42yrs on SC Birth	0.10	0.000	0.01	0.461
Phys Health on Age 16 Cog	0.12	0.000	0.13	0.000
Quals on Age 16 Cog	0.61	0.000	0.59	0.000
Age 16 Cog on Age 11 Cog	0.91	0.000	0.92	0.000

Model fit:
RMSEA 0.047
CFI 0.861
TLI 0.855

The first three paths are notable, but in the case of the last three the difference in the point estimates, though significant, is so small as to be unremarkable.

We see that men’s Age 50 cognitive ability at age 50 has a stronger link to age 42 SES than for women, where the path is not significant (strictly speaking, male p-value falls just sort of satisfying Bonferroni-corrected significance level .001, but female p-value is nowhere near).

In contrast, men’s Age 50 cognitive ability has a weaker link with Age 16 cognition (.42) than for women (.47).

Finally, for men there is a significant direct link from SC birth to SC 42 years, a result not significant for women.

For peer review only

BMJ Open

A Longitudinal Analysis of the 1958 British Birth Cohort Study examining the extent to which cognitive ability, behavioural problems and education in middle childhood and adolescence mediate the relationship between circumstances at birth and well-being outcomes at age 50 years

Journal:	*BMJ Open*
Manuscript ID	bmjopen-2019-031416.R1
Article Type:	Original research
Date Submitted by the Author:	07-Aug-2019
Complete List of Authors:	Dodgeon, Brian; UCL Institute of Education Centre for Longitudinal Studies, Social Science<br>Patalay, Praveetha; University College London, Centre for Longitudinal Studies<br>Ploubidis, George; University College London Institute of Education, Centre for Longitudinal Studies<br>Wiggins, Richard; University College London, Centre for Longitudinal Studies
**Primary Subject Heading**:	Public health
Secondary Subject Heading:	Mental health, Research methods, Sociology
Keywords:	MENTAL HEALTH, PUBLIC HEALTH, STATISTICS & RESEARCH METHODS, mid-life physical and emotional well-being

SCHOLARONE™
Manuscripts

I, the Submitting Author has the right to grant and does grant on behalf of all authors of the Work (as defined in the below author licence), an exclusive licence and/or a non-exclusive licence for contributions from authors who are: i) UK Crown employees; ii) where BMJ has agreed a CC-BY licence shall apply, and/or iii) in accordance with the terms applicable for US Federal Government officers or employees acting as part of their official duties; on a worldwide, perpetual, irrevocable, royalty-free basis to BMJ Publishing Group Ltd ("BMJ") its licensees and where the relevant Journal is co-owned by BMJ to the co-owners of the Journal, to publish the Work in this journal and any other BMJ products and to exploit all rights, as set out in our licence.

The Submitting Author accepts and understands that any supply made under these terms is made by BMJ to the Submitting Author unless you are acting as an employee on behalf of your employer or a postgraduate student of an affiliated institution which is paying any applicable article publishing charge ("APC") for Open Access articles. Where the Submitting Author wishes to make the Work available on an Open Access basis (and intends to pay the relevant APC), the terms of reuse of such Open Access shall be governed by a Creative Commons licence – details of these licences and which Creative Commons licence will apply to this Work are set out in our licence referred to above.

Other than as permitted in any relevant BMJ Author's Self Archiving Policies, I confirm this Work has not been accepted for publication elsewhere, is not being considered for publication elsewhere and does not duplicate material already published. I confirm all authors consent to publication of this Work and authorise the granting of this licence.

For peer review only

A Longitudinal Analysis of the 1958 British Birth Cohort Study examining the extent to which cognitive ability, behavioural problems and education in middle childhood and adolescence mediate the relationship between circumstances at birth and well-being outcomes at age 50 years

Corresponding author: Brian Dodgeon, Centre for Longitudinal Studies, UCL Institute of Education, 20 Bedford Way, London WC1H 0AL. b.dodgeon@ucl.ac.uk 020-7612-6877.
ORCID: 0000-0002-7130-8761

Co-authors:
Praveetha Patalay, Centre for Longitudinal Studies, UCL Institute of Education, 20 Bedford Way, London WC1H 0AL United Kingdom.
ORCID: 0000-0002-5341-3461

George B. Ploubidis, Centre for Longitudinal Studies, UCL Institute of Education, 20 Bedford Way, London WC1H 0AL United Kingdom.
ORCID: 0000-0002-8198-5790

Richard D. Wiggins, Centre for Longitudinal Studies, UCL Institute of Education, 20 Bedford Way, London WC1H 0AL United Kingdom.
ORCID: 0000-0007-2648-597X

Contributorship Statement

Brian Dodgeon performed the data management and structural equation modelling using the Mplus software, and took responsibility for completing all aspects of the paper.

Praveetha Patalay contributed to the conceptual planning of the analysis jointly with Richard Wiggins, performed preliminary regression modelling and advised on aspects of the structural equation modelling (SEM) strategy.

George Ploubidis advised on many aspects of the statistical analysis, and provided detailed comments on the drafting of the paper.

Richard Wiggins undertook the conceptual planning of the analysis jointly with Praveetha Patalay, and initiated the SEM approach, continuing to provide advice on the models as they became more complex, and collaborating with Brian Dodgeon on the write-up of the findings.

Word count:
3,211

ABSTRACT

1 Objectives

We aim to examine the relative contributions of pathways from early childhood/adolescence to mid-life well-being, health and cognition, in the context of family socio-economic status (SES) at birth, educational achievement and early-adulthood SES. We hypothesise that effects of childhood circumstances on mid-life physical & emotional well-being are strongly mediated by cognitive

For peer review only

performance during school years, with stronger mediated links for men than women from childhood-to-adult SES.

2 Setting/Participants

Data were the National Child Development Study, a fully-representative British Birth Cohort sample of 17,415 people born in one week in 1958.

3 Primary/secondary outcome measures

Our four primary mid-life outcome measures are: cognitive performance; physical and emotional well-being and quality of life. Our intermediate adult outcomes are early-adulthood social class and educational/vocational qualifications.

4 Results

Using structural equation modelling, we explore numerous pathways through childhood and early adulthood which are significantly linked to our outcomes. We specifically examine the mediating effects of the following: cognitive ability at ages 7, 11 and 16 years; childhood psychological issues ; family material difficulties at age 7: housing, unemployment, finance; educational/vocational qualifications and social class position at age 42 years.

We find that social class at birth has a strong indirect effect on the age 50 outcomes via its influence on cognitive ability, educational attainment and mid-life social class position, together with small direct effects on qualifications and social class position at age 42 years . Teenage cognitive performance has a strong positive effect on later physical health for women, whilst educational/vocational qualifications have a stronger positive effect on emotional well-being for men.

4 Conclusion

Our findings provide an understanding of the legacy of early life on multiple aspects of mid-life health, well-being, cognition and quality of life, showing stronger mediated links for men from childhood social class position to early adult social class position. The observed effect of qualifications supports those arguing that education is positively associated with subsequent cognitive functioning.

Article Summary

Strengths and limitations of this study:

- The richness of its longitudinal dimension, conveying the importance of early life circumstances upon early adult and mid-life measures of achievement, health and well-being;
- The holistic nature of the analysis, incorporating four outcomes, three of which are strongly correlated after the inclusion of several key early-life antecedents;
- It makes optimal use of all the available longitudinal data by employing Full Information Maximum Likelihood estimation to take account of item non-response;

For peer review only

- It shows the enduring importance of parental social class for cognitive performance and later life outcomes, controlling for family material difficulties in childhood
- A potential limitation is that our model does not include a broader set of demographic and psychosocial factors: a result of our decision to restrict the number of early-life influences to avoid over-complexity in the statistical modelling. There is also a small amount of attrition bias between birth and age 50.

INTRODUCTION

Authors since the 1990s have advanced the importance of a life course approach to explore the relationship between early life circumstances and later life health outcomes [1, 2]. Carr [3] emphasises how diverse early life experiences affect physical, emotional and cognitive health in later life by drawing upon a wealth of longitudinal data across the United States, Australia and Europe. Our empirical study is based on a single British Birth Cohort which covers several waves of data collection from birth (1958), middle childhood (age 7 & 11), adolescence (age 16) and mid-life at ages 42 and 50 years for over 8,000 sample members. Age 50 years represents a specific mid-point of mid-life (40-60) as defined by Midlife Development in the United States (MIDUS): Brim et al [4] suggest "Midlife has been described as the last uncharted territory of the life course". To this extent our conceptual framework is largely informed by Elder [5]: it may be described as a cumulative (dis-) advantage model [6] where we examine the legacy of early life circumstances, notably the British Registrar General's classification of occupations (RG social class) [7] as having lasting effects on health and well-being at age 50 years. Our approach implies a reciprocal relationship between socio-economic status (SES) and health, and allows for the possibility that favourable circumstances in later life can act counter to the effects of earlier disadvantage [8].

To this end we select outcomes at age 50 years covering four distinct areas: self-assessed quality of life, physical and emotional well-being, and a cognitive ability test.

Our research interest is in exploring the extent to which these outcomes inter-relate, adopting an empirical view that, taken together, these measures would tell us more about the consequences of the past than studied individually [9]. There is a growing body of evidence based on United Kingdom (UK) longitudinal studies [10,11,12] that provides a strong argument for combining these outcomes in order to provide a holistic perspective: in particular, Cooper et al [13] provided both substantive and empirical evidence for examining gender differences in this analysis.

Drawn from the same rich longitudinal resource, our predictors and mediators begin with birth circumstances: birthweight, breastfeeding, maternal smoking during pregnancy and parental SES. From the age 7 sweep of the study we include the effects of family material difficulties such as unemployment, finance and housing, in conjunction with childhood scores of socio-emotional adjustment and cognitive performance, each measured on three occasions spanning the ages 7, 11 and 16 years. The inclusion of SES at age 42 and lifetime highest qualification in the model enables us to trace which childhood effects persist, and which are built upon or attenuated by education and social mobility.

Our analysis builds upon the framework adopted by Wood et al [14] on the prediction of mental well-being as a single outcome [15] across four UK birth cohort studies, which showed that

For peer review only

childhood SES is directly and indirectly (through adult socioeconomic pathways) linked to adult emotional well-being. We expand their approach by having a fuller set of four outcome measures, albeit for a single birth cohort. Taken together, we examine the pathways from childhood to our multivariate mid-life outcome measures for men and women.

Our conceptual framework is summarised in Fig.1. The inter-relationships between the latent variables, and in particular the interplay between our outcomes, define a structural equation model (SEM) which underpins our statistical analysis [16].

Fig. 1 Conceptual model of pathways from early childhood to mid-life outcomes of physical and emotional well-being, quality of life and cognitive performance at age 50 years

[insert Fig. 1 here]

DATA AND METHODS

Our data source, the British National Child Development Study (NCDS) is fully representative of the population of Great Britain (GB), having interviewed the parents of 98.5% of all babies born in the same week of 1958 [17], and followed them up longitudinally with ten subsequent interviews throughout life. It provides operational measures regarding birth circumstances, the early and teen years covering ages 7, 11 and 16, and adulthood at ages 42 and 50 respectively.

The analysis is based upon 3815 male and 4209 female cohort members present at each occasion who all have at least one genuine response for each of the four separate age 50 life domains, and who also participated in at least one of the three cognitive and socio-emotional assessments, in order to permit a reliable strategy for handling missing items.

We are aware there is a certain amount of attrition bias as a result of differential loss-to follow-up from the 17,415 cohort members present at the birth study in 1958. As a check on the impact of sample loss, we were reassured by the close match of the distribution of the birth social class variable within our sample of 8,024 against that of the same variable within the full 17,415 birth sample (chi-square=1.29, 5d.f., non-significant) (see Appendix 1).

Our outcomes at age 50 years cover four distinct measures, the first two being self-reported physical and emotional well-being (PWB and EWB respectively) measured using sub-scales [18] of the RAND 36-Item Short-Form Health Survey [19-23] (hereafter SF-36). Third, a self-reported measure of quality of life (QoL) covering the life domains of 'Control, Autonomy, Self-Realisation and Pleasure (CASP-12 v2) [24]: a theoretically-informed measure of subjective well-being, closely following the approach adopted by Ed Diener and his colleagues [25]. The fourth domain outcome, cognitive ability (hereafter Age50Cog) consists of four cognitive tests examining memory (word recall & delayed recall), executive function (animal naming), attention and mental speed (letter cancellation) [26].

Appendix 2 lists all of the manifest and latent variables employed in the analysis. Indicators for cognitive function at age 50 years are listed here, while those for physical, emotional and quality of life outcomes are expanded upon more fully in Appendix 3. Childhood behavioural indicators are listed in Appendix 4 following Rutter [27]. We recognise of course that Rutter scores may be sub-

For peer review only

divided into constituent dimensions of problem behaviour (withdrawn/ anxious / oppositional/ inattentive [28], but for this paper we prefer to use Rutter total scores, following Parsons et al [29].

In fitting our structural equation models using the MPlus software [30] we obtain direct and indirect (mediated) effects of our background variables in terms of childhood social origins and social adjustment, on outcomes in early adulthood, and mid-life at age 50 years. We regard all four of our birth indicators as primary predictors, valuing the opportunity to assess the relative impact of each for policy interventions [31]. Our birth social class variable is based on father's occupation, since in 1958 when the cohort were born, only 38% of mothers were economically active.

The sets of predictors determining our paths differ slightly for each of the four outcomes so as to avoid the problem of 'seemingly unrelated regression' [32]

We employ full information maximum likelihood estimation (FIML) [33]. This addresses the issue of item non-response arising where questions are avoided or not answered by certain participants.

The SEM procedure that follows begins with an analysis of the sample as a whole, followed by separate analyses for men and women, supported by a multigroup analysis [34] to test for the structural equivalence of the models for men and women. Model 'goodness of fit' is assessed via conventional criteria: 'root mean square error' (RMSEA), 'comparative fit index', (CFI) and 'Tucker Lewis fit index', (TLI) where RMSEA<0.05, CFI>0.90 and TFI> 0.90 would provide evidence of 'good fit' [35].

Having a large number of SEM pathways involving 261 free parameters, we had regard to the n:q rule [36] and were satisfied that our sample-size-to-parameters ratio was comfortably within Jackson's guideline of 10:1. Or application of SEM involved the use of modification indices, which led to our running correlations on suggested variables. All our final models were identified, all manifest variables loading onto the respective latent variables with satisfactory fit. The details of the individual assessments of our measurement models can be made available on request.

Patient and Public involvement statement

Participant consent was obtained from parents of cohort members at the initiation of the original birth cohort study in 1958. On attaining adulthood, permission was obtained from cohort members themselves at each subsequent longitudinal sweep.

RESULTS

The results begin with consideration of the inter-correlations between our mid-life outcome measures. Further univariate descriptives are contained in Appendix 2. Figure 2 contains a full SEM diagram for the sample as a whole. Subsequent reference will be made to Appendix 5 for the details of mediation, total and indirect effects. Appendix 6 contains separate analyses by sex and Appendix 7 contains further information on our multigroup analysis based on gender.

(a) Descriptives & correlations

For peer review only

As a preliminary inspection of the inter-relationships between our four outcome measures, Table 1 contains a correlation matrix of the four summative indices based on these measures for both men and women.

Table 1. Associations between the four sets of mid-life outcomes as summative indices (Men/Women)

Age 50 Domain	Age 50 Cog	PWB	EWB	QoL
Cognitive Ability (Age 50 Cog)	1.00/1.00			
Physical Well-Being (PWB)	0.12/0.15	1.00/1.00		
Emotional Well-Being (EWB)	0.12/0.09	0.57/0.58	1.00/1.00	
Quality of Life (QoL)	0.15/0.10	0.48/0.48	0.63/0.64	1.00/1.00

Interestingly, QoL, PWB and EWB demonstrate moderate associations of inter-correlation. While cognitive ability is positively related to the physical and emotional well-being measures, the strength of the association is modest, with PWB having a higher coefficient for women and EWB & QoL a higher coefficient for men.

Fig. 2 contains estimates of association amongst these outcomes as defined by measurement models under a full SEM analysis.

(b) Birth social class effects on early mid-life outcomes

The path diagram shown in Fig 2 provides the model results for the sample as a whole showing only statistically significant paths at a p-level of .001, employing a Bonferroni correction for the fifty path coefficients being estimated (0.05/50=0.001) [37].

Fig 2. Estimated pathways of the Structural Equation Model, N=8024 (showing paths significant at p < .001)

[insert Fig.2 here]

Whilst there are various pathways that connect early life circumstances with cognitive performance, family material difficulties and social adjustment, there remains a small direct effect of social class at birth on each of our two early adult outcomes: Age 42 Social class and educational/vocational qualifications (both path coefficients equal 0.05). These two 'interim' or 'pivotal' destinations, in turn, have notable effects on our four mid-life outcomes at age 50, which we describe under (d) below.

(c) Indirect effects via pathways through childhood mediators

Inspecting the paths in Fig.2 from birth circumstances through ages 7, 11 and 16, we see firstly, that all four of our birth characteristics have direct effects on cognitive performance at age 7 years, when children are in primary school. Social class at birth has the most notable association with age 7

For peer review only

cognition (.27). This cognitive variable in turn has a strong direct effect on age 11 cognition (.92) and similarly for age 11 on cognitive performance at age 16 (.92).

There is a clear path via cognitive ability throughout childhood and adolescence, to adult achievement as indicated by Age 42 social class and educational qualifications (Age 16 Cog to Social class 42yrs .50, Age 16 Cog to Quals .59).

This legacy of childhood cognitive performance is unsurprising, but interestingly Age 16 cognition also has a direct association with age 50 physical well-being (.12) besides more naturally, Age 50 cognition (.47).

Family material difficulties in childhood, themselves associated with birth social class (-0.26), also play their part in understanding a child's trajectory in cognitive performance. Characterised by issues with housing, finance and employment when a child is 7 years old, these family material difficulties are seen to have negative consequences for cognitive performance at age 7 years (-.15) and a lagged negative effect on later performance age 16 years (-0.04).

Additional analyses also show that family material difficulties impact on a child's emotional and behavioural scores (based on Rutter) at ages 7, 11 and 16 (.09, .06 and .10 respectively). In turn these scores had small significant negative associations with cognitive performance at each corresponding age (-.13, -.04 and -.05 respectively). But most notably, Rutter scores at these three ages are significantly correlated (.49 for age 7 to 11 and .44 for age 11 to 16), and there are direct effects from age 16 Rutter scores to three of our four age 50 outcomes: EWB (-0.13), QoL (-0.10) and PWB (-0.07).

(d) Indirect effects via adulthood mediators

Adult social class and lifetime qualifications are, together, influential predictors of each of our four mid-life outcomes: adult social class has significant predictive effects (.08 and .11) on PWB and QoL, and our Bonferroni-corrected criterion of $p < .001$ narrowly excludes its link to EWB (.05 at $p<.004$).

In general, the socially-advantaged enjoy better health and report higher levels of quality of life than their less advantaged counterparts. Interestingly, qualifications predict age 50 cognitive ability (.05), EWB (.06) and QoL (.10) but not PWB (.04 at $p<.01$).

(e) Gender differences

Our multigroup analysis reveals that certain paths have significantly different effects for men and women. In Appendix 6 we display two separate path diagrams for men and women. For men, the direct effect of social class at birth on social class and qualifications in early adulthood is notably stronger (.09 and .07 respectively) than for women (.05, .05).

Both sexes see the birth social class effect remain equally strong for cognitive performance at age 7 years and beyond, but women experience a slightly worse negative effect of social disadvantage at birth on the latent variable 'family material difficulties' (-0.27 compared to -0.24 for men). In turn, family material difficulties have slightly more influence on Age 7 cognition (-.17) for men than for women (-.14), and a small lagged effect on Age 16 cognition only evident for women. After age 7

For peer review only

the Rutter social adjustment scores do not appear to impact upon the cognitive performance scores for men, but they do for women at 11 and 16, although the effect is small (.05).

Early adult social class has a slightly stronger influence for men than women on our age 50 outcomes PWB and QoL, and Qualifications have a significant effect on EWB for men but not for women. In contrast, women's age 16 cognition has a significant effect on their later PWB (.17), an effect not significant for men; and the path from age 16 cognition to age 50 Cog has a stronger coefficient for women (.47) than men (.43).

We pursue the evidence for gender differences via a sensitivity analysis under MPlus which formally tests for statistical difference between paths for each sex. Appendix 7 identifies six paths where the effects for men and women are significantly different, but only three have notable differences in terms of their effect sizes in the multigroup analysis. These are:

- stronger effect for women from age 16 cognition to age 50 cognition as noted above (.47 to .43);
- direct effect of social class at birth on early social class (much stronger for men); and
- small effect of age 42 social class on Age 50 Cognition (.07 at p<.002 for men, no significant effect women)

DISCUSSION

Structural equation modelling has elucidated the nuances of the influence of pathways from childhood and early adulthood to our four related outcomes of health, well-being and cognition in mid-life. The work demonstrates the benefits of a holistic approach to defining the aspects of the life course that affect well-being in mid-life. Physical and emotional health are important factors affecting a person's subjective account of their quality of life, and PWB correlates at .69 with EWB, which in turn correlates .83 with QoL. While cognitive performance at age 50 is relatively weakly correlated, the other three are closely intertwined. These associations are similar in magnitude to those reported in table 1; however they are now estimated by taking the legacy of the life course into account.

We also show that some of the paths to health and well-being in mid-life are different for men and women [38]. However, important communalities remain.

Social origins, in particular social class at birth, have a major indirect influence on mid-life outcomes, the mediation process beginning with a child's cognitive performance throughout formal schooling and subsequently via cognitive performance at age 16 years. Collectively, these influences are weakened or reinforced by the existence of family material difficulties in early childhood (themselves influenced by childhood social class). Whilst cognitive performance is a strong predictor of early adult social class and educational status for both men and women, social class at birth also has a lasting association with these mid-life indicators: more so for men than for women.

This could well suggest that women have a looser connection with the legacy of social class at birth, perhaps partly because their path to advantaged SES at age 42 may be impeded by caring responsibilities and gender inequalities in the labour market [39].

Early adult social class influences all four of our mid-life outcomes, but we see the direct effect of social class at birth on social class and qualifications in early adulthood is notably stronger for men (.09 and .07 respectively) than for women (.05, .05).

A relatively small but consistent direct effect of qualifications on Age 50 Cognition supports those arguing that education is positively associated with subsequent cognitive functioning [40], although we are aware that there are counter-arguments to this view [41]). Equally, the relationship between QoL and early adult social class finds support in the work of Blane et al [42].

The strength in the evidence presented here lies in the richness of its longitudinal dimension which conveys the importance of early life circumstances upon early adult and mid-life measures of achievement, health and well-being.

One limitation of our study is that our model does not fully take into account all of the potential demographic and psychosocial factors other researchers have studied as predictors of satisfactory midlife development [28]. Nevertheless, it can be argued that our birth social class variable acts as a 'proxy' for certain parental beliefs and behaviours [43]. We are embarking on developing a conceptual framework and modelling strategy to extend our empirical analyses in the future as our cohort members age: in particular, our subjects are not yet old enough for us to assess whether any decline in cognitive performance at age 50 years will lead to subsequent decline and become more influential on emotional well-being and quality of life. However, we were able to show that cognitive performance in the teen years holds a legacy for cognitive performance in mid-life. A final limitation is a certain amount of attrition bias between birth and age 50 (differential non-response) which was addressed earlier in the paper and in Appendix 1.

Social class at birth has a strong influence on early cognitive performance and beyond, which in turn has consequences for adult achievement and social class. Clearly, the presence of family material difficulties and behavioural issues will impede (mediate) this trajectory. Interestingly, childhood behaviour, in particular at age 16, has a lasting effect on mid-life EWB and Quality of Life.

In sum, health, emotion, quality of life and to a lesser extent cognitive function do not stand alone as markers of well-being in mid-life. They represent the constituents of an inter-related whole which is shaped by early life circumstances, family material difficulties, social adjustment and cognitive performance in childhood.

References

1 Williams, D.R. (1990). Socioeconomic differentials in health: A review and redirection. Social Psychology Quarterly 53, 81–99.

2 Wadsworth, M. E. J. (1997). Health inequalities in the life course perspective. Social Science & Medicine 44, 859–869.

3 Carr, D (2019). Early-life influences on later life well-being: innovations and explorations. The Journals of Gerontology, Series B: Psychological Sciences and Social Sciences, 74(5), 829-831.

4 Brim, O, Ryff, G, Kessler, C.D & Ronald, C (2004). How healthy are we? A National Study of Well-Being at Midlife. Chicago, IL: University of Chicago Press.

For peer review only

5 Elder, G (1994). Time, human agency, and social change: perspectives on the life course. Social Psychology Quarterly, 57, 4-15.

6 Dannefer, D (2003) Cumulative advantage/disadvantage and the life course: Cross-fertilizing age and social science theory. The Journals of Gerontology, Series B, 58(6), S327-S337.

7 Szreter, S.R.S (1984). The Genesis of the Registrar-General's Social Classification of Occupations. British Journal of Sociology 35(4), 522-54

8 Graham, H. (2002). Building an inter-disciplinary science of health inequalities: The example of lifecourse research. Social Science & Medicine 55(11), 2005–2016.

9 Marmot, M. G, Fuhrer, R, Ettner, S.L, Marks, N.F, Bumpass, L.L and Ryff, C.D (1998) Contribution of psychosocial factors to socioeconomic differences in health. Millbank Quarterly 76, 403-48.

10 Gale, C. R, Cooper, R, Craig, L, Elliott, J, Kuh, D, Richards, M & Deary, I. J (2012). Cognitive function in childhood and lifetime cognitive change in relation to mental wellbeing in four cohorts of older people. *PLoS ONE*, *7*(9), e44860. doi: 10.1371/journal.pone.0044860

11 Clouston, S. A, Brewster, P, Kuh, D, Richards, M, Cooper, R, Hardy, R, Rubin, M.S and Hofer, S M (2013). The Dynamic Relationship Between Physical Function and Cognition in Longitudinal Aging Cohorts. *Epidemiologic Reviews* 35(1), 33–50 doi: 10.1093/epirev

12 Cooper, R, Stafford, M, Hardy, R, Aihie Sayer, A, Ben-Shlomo, Y, Cooper, C & the HALCyon Study Team (2014). Physical capability and subsequent positive mental wellbeing in older people: findings from five HALCyon cohorts. *Age 36*(1), 445-456. doi: 10.1007/s11357-013-9553-8

13 Cooper, R, Hardy, R, Aihie Sayer, A, Ben-Shlomo, Y, Birnie, K. C, Craig, L, Deary, I.J, Demakakos, P, Gallacher, J, McNeill, G, Martin, R.M, Starr, J.M, Steptoe, A, Kuh, D and the HALCyon Study Team (2011). Age and Gender Differences in Physical Capability Levels from Mid-Life Onwards: The Harmonisation and Meta-Analysis of Data from Eight UK Cohort Studies. ***PLoS One***, 6(11), e27899.

14 Wood, N, Bann, D, Hardy et al (2017) Childhood socioeconomic position and adult mental wellbeing: evidence from British birth cohort studies. PLoS One, 10, e0185798.

15 Tennant, R, Hiller, L, Fishwick, R et al (2007) The Warwick-Edinburgh Mental Well-Bing Scale (WEMWBS): development and UK validation. Health and Quality of Life Outcomes 5(1), 63.

16 Kline, R.B. (1998). *Principles and Practice of structural equation modelling*. New York. Guildford Press.

17 Power, C and Elliott, J. (2006) Cohort Profile: 1958 British birth cohort (National Child Development Study). International Journal of Epidemiology 35(1), 34-41.

18 Doll, H.A, Petersen, S.E.K and Stewart-Brown, S.L (2012) Obesity and Physical and Emotional Well-Being: Associations between Body Mass Index, Chronic Illness, and the Physical and Mental Components of the SF-36 Questionnaire. Obesity 8(2), 160-170 doi: 10.1038/oby.2000.17

19 Ware, J.E and Donald Sherbourne, C (1992). The MOS 36-Item Short-Form Health Survey (SF-36): I. Conceptual Framework and Item Selection. Medical Care 30(6), 473-483.

20 Brazier, J.E, Harper, R, Jones et al (1992). Validating the SF-36 health survey questionnaire: new outcome measure for primary care. British Medical Journal 305(6846): 160-164.

21 Jenkinson, C., Wright, L. and Coulter, A. (1994). Criterion validity and reliability of the SF-36 in a population sample. Quality of Life Research 3(1), 7-12.

22 Burnholt, V & Nash, P. (2011). Short Form 36 (SF-36) Health Survey Questionnaire: normative data for Wales. Journal of Public Health, 33(4), 587-683.

23 Mishra, G.D, Gale, C.R, Aihie Sayer, A, Cooper, C, Dennison, E. M, Whalley, L.J, Craig, L, Kuh, D, Deary, I. J and the HALCyon Study Team (2011) How useful are the SF-36 sub-scales in older people? Mokken scaling of data from the HALCyon programme. *Quality of Life Research*, 20 (7), 1005-1010. (doi:10.1007/s11136-010-9838-7). (PMID:21225350)

24 Wiggins, R.D, Netuveli, G., Hyde et al (2008). The evaluation of a self-enumerated scale of quality of life (CASP-19) in the context of research on ageing: a combination of exploratory and confirmatory approaches. Social Indicators Research 89(1), 61-77.

25 Diener, E and Suh, E (1997) Measuring quality of life: Economic, social, and subjective indicators Social indicators Research, 40 (1-2), 189-216.

26 Brown, M and Dodgeon, B. (2010) NCDS Cognitive Assessments at Age 50: Initial Results. CLS Working Paper 2010/1. London: Centre for Longitudinal Studies. UCL.

27 Rutter, M, Tizard, J and Whitmore, K (1970) Education, Health & Behaviour, Longman.

28 Caspi, A, Henry, B, McGee, R.O, Moffitt, T.E & Silva, P (1995). Temperamental Origins of Child and Adolescent Behavior Problems: From Age Three to Age Fifteen Child Development 66 (1), 55-68 doi: 10.1111/j.1467-8624.1995.tb00855.x

29 Parsons, S, Green, F, Ploubidis, G.B, Sullivan, A and Wiggins, R.D (2017). The influence of private primary schooling on children's learning: Evidence from three generations of children living in the UK. British Educational Research Journal 43(5), 823-847 (doi: 10.1002/berj.3300)

30 Muthen, B.O & Muthen L.K (2018) Statistical Analysis with Latent Variables: MPlus v8.1 User Manual. Los Angeles CA: Muthen & Muthen.

31 Sacker, A, Kelly, Y, Iacovou, M, Cable, N and Bartley, M. (2013) Breast feeding and intergenerational social mobility: what are the mechanisms? ***Archives of Disease in Childhood***, 98(9), 666-671.

32 Zellner, A. 1962. An efficient method of estimating seemingly unrelated regression equations and tests for aggregation bias. Journal of the American Statistical Association 57: 348–368.

33 Arbuckle, J.L. (1996). Full Information estimation in the presence of incomplete data. In Marcoulides, G.A & & Schumaker, R.E (eds) *Advanced Structural Equation Modeling: Issues and Techniques.* Mahwah, N.J.: Erlbaum Associates.

34 Byrne, B.M. (2012). *Structural Equation Modeling with Mplus*. Routledge Taylor & Francis Group. New York & London

35 Bollen, K.A. & Scott Long, J. (eds) (1993). *Testing Structural Equation Models*. SAGE Focus edition. Newbury Park, London & New Delhi.

36 Jackson, D,L (2003) Revisiting Sample Size and Number of Parameter Estimates: Some Support for the N:q Hypothesis. Structural Equation Modeling 10(1), 128-141.

37 Weisstein, Eric W (2004) "Bonferroni Correction." From MathWorld--A Wolfram Web Resource. http://mathworld.wolfram.com/BonferroniCorrection.html

38 Arber, S. & Cooper, H. (1999). Gender differences in health in later life: the new paradox? Social Science and Medicine, 48(1), 61-76

39 Kahn, J.R, Garcia-Manglano, J and Bianchi, S.M (2014) The Motherhood Penalty at Midlife: Long-Term Effects of Children on Women's Careers. Journal of Marriage and the Family 76(1), 56-72.

40 Richards, M and Sacker, A (2010) Is education causal? Yes. International Journal of Epidemiology 40(2), 516–518.

41 Deary, I.J and Johnson, W (2010) Intelligence and education: causal perceptions drive analytic processes and therefore conclusions. International Journal of Epidemiology 39(5), 1362-1369.

42 Blane, D, Netuveli, G and Bartley, M (2007). Does quality of life at older ages vary with socio-economic position? Sociology **41**(4), 717-726.

43 Davis-Kean, P.E (2005). The Influence of Parent Education and Family Income on Child Achievement: The Indirect Role of Parental Expectations and the Home Environment. Journal of Family Psychology, 19(2), 294-304.

Footnotes

Funding

Assistance is acknowledged from ESRC grants ES/N003683/1 and ES/M008584/1.

Consent of authors

I, Brian Dodgeon, the Submitting Author, have the right to grant and do grant on behalf of all authors of the Work (as defined in the below author licence), an exclusive licence and/or a non-exclusive licence for contributions from authors who are: i) UK Crown employees; ii) where BMJ has agreed a CC-BY licence shall apply, and/or iii) in accordance with the terms applicable for US Federal Government officers or employees acting as part of their official duties; on a worldwide, perpetual, irrevocable, royalty-free basis to BMJ Publishing Group Ltd ("BMJ") its licensees and where the relevant Journal is co-owned by BMJ to the co-owners of the Journal, to publish the Work in BMJ Open and any other BMJ products and to exploit all rights, as set out in our licence.

For peer review only

The Submitting Author accepts and understands that any supply made under these terms is made by BMJ to the Submitting Author unless you are acting as an employee on behalf of your employer or a postgraduate student of an affiliated institution which is paying any applicable article publishing charge ("APC") for Open Access articles. Where the Submitting Author wishes to make the Work available on an Open Access basis (and intends to pay the relevant APC), the terms of reuse of such Open Access shall be governed by a Creative Commons licence – details of these licences and which Creative Commons licence will apply to this Work are set out in our licence referred to above.

Competing interests

All four authors declare they have no competing interests and no financial relationships with any organisation that might have an interest in the submitted work in the previous three years, and no other relationships or activities that could appear to have influenced the submitted work.

Data Sharing statement

The analysis is based on secondary data available for download from the UK Data Archive at https://beta.ukdataservice.ac.uk/datacatalogue/series/series?id=2000032. Syntax used to derive variables for this analysis can be obtained by e-mailing the corresponding author, Brian Dodgeon.

Ethics

Ethical approval was not required, since the study used the secondary data of the National Child Development Study, which itself had received ethics consent from the London Research Ethics Committee, ref 08/H0718/29 for its age 50 survey, the source of our outcome data. Multicentre Research Ethics Committee (MREC) approval was received for earlier NCDS surveys.

For peer review only

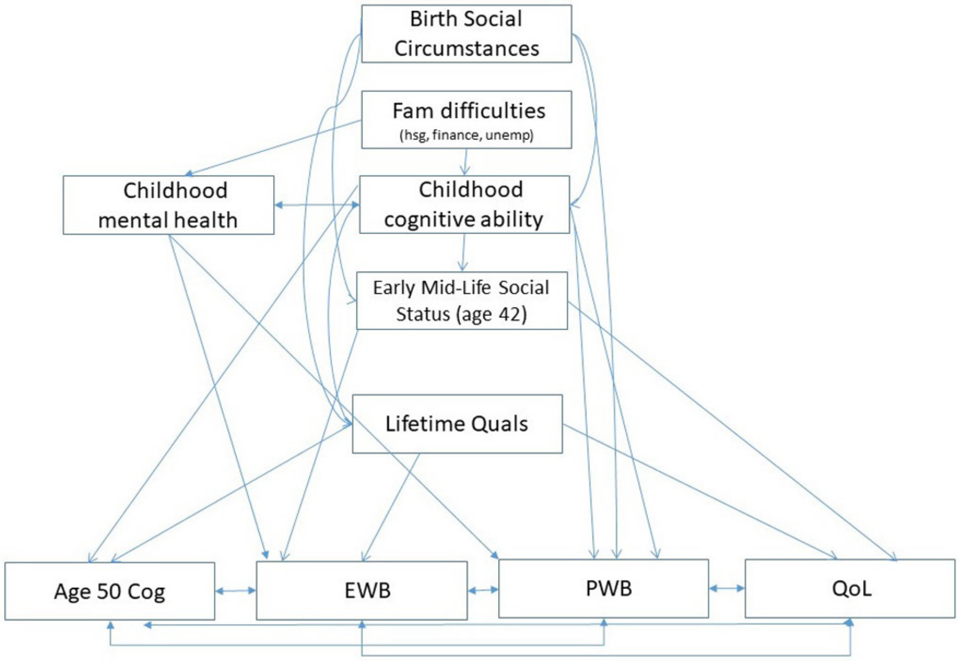

Fig.1 Conceptual model of Pathways from early childhood to mid-life outcomes of physical and emotional well-being, quality of life and cognitive performance at age 50 years

90x60mm (300 x 300 DPI)

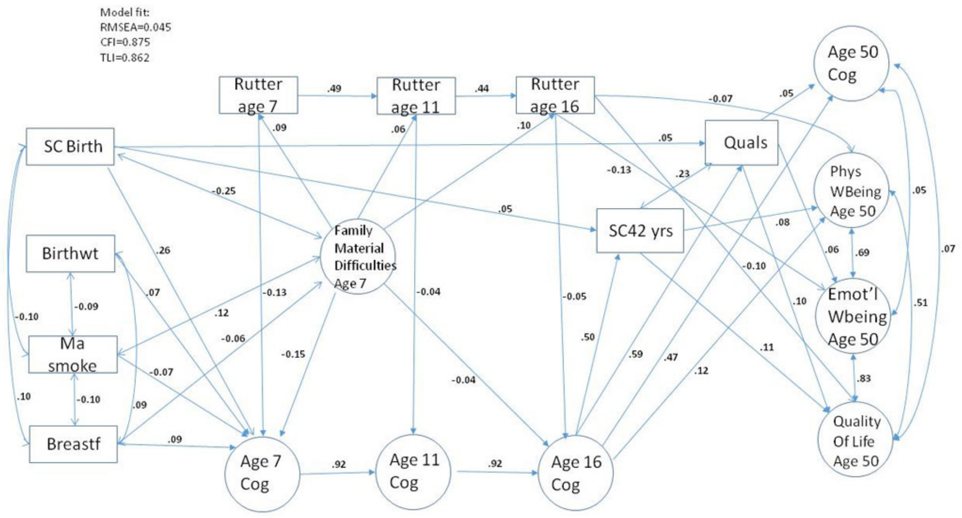

Fig. 2 Estimated pathways of the Structural Equation Model, N=8024 (showing paths significant at p < .001)

90x50mm (300 x 300 DPI)

Appendix 1

Check for attrition bias between full NCDS sample at birth and Analysis sample

Comparison of distributions of the same birth social class variable:

Father's social class at birth	Full birth survey N=17,415	Valid %	Analysis sample N=8024	Valid %
Class I (Professional)	746	4.5	386	5.2
Class II (Intermediate)	2133	13.0	1127	15.1
Class III (Skilled Manual/Non-Manual)	9981	60.6	4498	60.1
Class IV (Semi-Skilled Manual)	1995	12.1	871	11.6
Class V (Unskilled Manual)	1616	9.8	603	8.1
Total (valid Sclass)	16471	100.0	7485	100.0
Insufficient data on parental Social Class	944		539	
Total	17415		8024	

(chi-square=1.29, 5d.f., non-significant)

For peer review only

Appendix 2: Description of manifest variables, age CM at data collection; latent variable labelling

Variable	Survey Sweep	Range of recorded codes	Mean	St'd Dev'	% missing	Latent Variable
Early years						
Social class of father (GRO 1951)	Birth	1-6 (1=low)	2.98	0.92	6.7	
Birthweight	Birth	1 <2.5 kg<br>2 >=2.5kg	1.94	0.23	2.8	
Did mother smoke after 4th month of pregnancy	Birth	1-4(No/Variable Med'm/Heavy)	1.7	1.10	3.6	
Sex	Birth	1,2	52.5% female		0	
Mother breastfed child	Age 7	1 No; 2 <1 mth; 3 >1 month	2.15	0.86	10.4	
Problem arithmetic score	Age 7	0-10	5.38	2.43	9.1	Age7cog
Copying designs test age 7	Age 7	0-12	7.19	1.93	9.1	
Draw-A-Man test score	Age 7	0-51	24.40	6.95	10.6	
Southgate reading test score	Age 7	0-30	24.43	6.36	8.9	
Fam difficulties by age 7: Housing (health visitor assess't)	Age 7	0 No; 1 D/K 2Yes	0.17	0.51	9.9	Family Material Difficult's Age 7
Fam difficulties by age 7: Finance	Age 7	0,1,2	0.21	0.53	10.0	
Fam difficulties by age 7: Unempl	Age 7	0,1,2	0.11	0.38	10.0	
NFER general ability test	Age 11	0-80	46.14	15.2	12.0	Age11cog
Reading Comprehension score	Age 11	0-40	16.97	5.95	12.0	
Maths score	Age 11	0-40	18.40	10.1	12.0	
Copying designs test age 11	Age 11	0-12	8.45	1.40	12.2	
Rutter behavioural score (age 7) (mother assess't at each age)	Age 7	0-28	6.13	3.50	9.7	
Rutter behavioural scor (age 11)	Age 11	0-28	6.13	3.41	12.8	
Rutter behavioural score (age 16	Age 16	0-36	3.91	3.42	24.4	
Adolescence						
Reading Comprehension age 16	Age 16	0-40	26.58	6.23	23.1	Age16cog
Maths test age 16	Age 16	0-40	13.83	6.96	23.4	
Teacher assess't English ability	Age 16	1-5	3.44	1.12	22.9	
Teacher assess't Maths ability	Age 16	1-5	3.11	1.19	23.5	
Early Mid-Life						
Social class at age 42	Age 42	1-6 (1=low)	3.99	1.24	13.9	
Later Mid-Life						
Highest qual'n (acad or vocat) achieved by age 50	Age 50	0-5 (0=no quals; 1-5=NVQ)	2.68	1.36	0	
Latter cancellation speed	Age 50	0-800	334.60	87.8	3.5	Age50cog
Word recall	Age 50	0-10	6.60	1.46	2.1	
Delayed word recall	Age 50	0-10	5.49	1.81	2.7	
Animal naming test	Age 50	0-65	22.53	6.22	2.1	
SF-36 phys. health (4 scores: see Appx.1. Summative stats shown)	Age 50	0-100 (each of 4 scores)	315.4	83.8	0.2	PhysWbeing Age 50
SF-36 mental health (4 scores)	Age 50	0-100 (4 scores)	276.6	63.3	0.3	Emot'l Wbeing Age 50
CASP-12 (3 control scores, 3 autonomy, 3 pleasure, 3 self-realisation – see Appx.1	Age 50	0,1,2,3 (each of 12 scores)	23.94	5.42	0.7	Quality of Life Age 50

For peer review only

Appendix 3. Wording of SF-36 scale items allocated to combined life domains of Physical Health (PH) and Emotional Well-Being (EWB) and Quality of Life based on CASP-12 (v2) items

SF-36 Item	Wording
1 Self-assessed General Health<br>*(Domain in our model: Phys Health)*	In general, would you say your health is:<br>(1) Excellent; (2) Very good; (3) Good;<br>(4) Fair; (5) Poor
2 Self-assessed General Health compared to one year ago<br>*(Domain: Phys Health)*	2. Compared to one year ago, how would you rate your health in general now?<br>(1) Much better now than one year ago;<br>(2) Somewhat better now than 1 year ago;<br>(3) About the same;<br>(4) Somewhat worse now than 1 year ago;<br>(5) Much worse now than one year ago
Health now limiting specific physical activities:<br><br>3. Vigorous activities?<br>*(Domain: Phys Health)*<br><br>4. Moderate activities?<br>*(Domain: Phys Health)*<br><br>5. Lifting or carrying groceries?<br>*(Domain: Phys Health)*<br>6. Climbing several flights of stairs?<br>*(Domain: Phys Health)*<br>7. Climbing one flight of stairs?<br>*(Domain: Phys Health)*<br>8. Bending, kneeling, or stooping?<br>*(Domain: Phys Health)*<br><br>9. Walking more than a mile?<br>*(Domain: Phys Health)*<br><br>10. Walking several blocks?<br>*(Domain: Phys Health)*<br><br>11. Walking one block?<br>*(Domain: Phys Health)*<br><br>12. Bathing or dressing yourself?<br>*(Domain: Phys Health)*	The following items are about activities you might do during a typical day. Does your health now limit you in these activities? If so, how much?<br>3 Vigorous activities, such as running, lifting heavy objects, participating in strenuous sports<br>(1) Yes, limited a lot;<br>(2) Yes, limited a little;<br>(3) No, not limited at all<br>4. Moderate activities, such as moving a table, pushing a vacuum cleaner, bowling, or playing golf?<br>Answer (1)(2)(3)<br>5. Lifting or carrying groceries?<br>Answer (1)(2)(3)<br>6. Climbing several flights of stairs?<br>Answer (1)(2)(3)<br>7. Climbing one flight of stairs?<br>Answer (1)(2)(3)<br>8. Bending, kneeling, or stooping?<br>Answer (1)(2)(3)<br>9. Walking more than a mile?<br>Answer (1)(2)(3)<br>10. Walking several blocks?<br>Answer (1)(2)(3)<br>11. Walking one block?<br>Answer (1)(2)(3)<br>12. Bathing or dressing yourself?<br>Answer (1)(2)(3)
During the past 4 weeks, have you had any of the following problems with your work or other regular daily activities as a result of your physical health?<br>13. Cut down the amount of time you	13. Cut down the amount of time you spent on work or

For peer review only

spent on work or other activities?<br>*(Domain: Phys Health)*<br>14. Accomplished less than you would like<br>*(Domain: Phys Health)*<br>15. Were limited in the kind of work or other activities<br>*(Domain: Phys Health)*<br>16. Had difficulty performing the work or other activities<br>*(Domain: Phys Health)*	other activities?<br>(1) Yes (2) No<br>14. Accomplished less than you would like?<br>(1) Yes (2) No<br>15. Were limited in the kind of work or other activities<br>(1) Yes (2) No<br><br>16. Had difficulty performing the work or other activities (for example, it took extra effort)<br>(1) Yes (2) No
During the past 4 weeks, have you had any of the following problems with your work or other regular daily activities as a result of any emotional problems (such as feeling depressed or anxious)?<br><br>17. Cut down the amount of time you spent on work or other activities<br>*(Domain: Emotional WBeing)*<br>18. Accomplished less than you would like<br>*(Domain: Emotional WBeing)*<br>19. Didn't do work or other activities as carefully as usual<br>*(Domain: Emotional WBeing)*	17. Cut down the amount of time you spent on work or other activities?<br>(1) Yes (2) No<br>18. Accomplished less than you would like?<br>(1) Yes (2) No<br>19. Didn't do work or other activities as carefully as usual?<br>(1) Yes (2) No
20. During the past 4 weeks, to what extent has your physical health or emotional problems interfered with your normal social activities with family, friends, neighbours, or groups?<br>*(Domain: Emotional WBeing)*	20. During the past 4 weeks, to what extent has your physical health or emotional problems interfered with your normal social activities with family, friends, neighbours, or groups?<br>(1) Not at all;<br>(2) Slightly;<br>(3) Moderately;<br>(4) Quite a bit;<br>(5) Extremely
21. How much bodily pain have you had during the past 4 weeks?<br>*(Domain: Phys Health)*	21. How much bodily pain have you had during the past 4 weeks?<br>(1) None;<br>(2) Very mild;<br>(3) Mild;<br>(4) Moderate;<br>(5) Severe;<br>(6) Very severe
22. During the past 4 weeks, how much did pain interfere with your normal work (including both work outside the home and housework)?<br>*(Domain: Phys Health)*	22. During the past 4 weeks, how much did pain interfere with your normal work (including both work outside the home and housework)?<br>(1) Not at all;<br>(2) A little bit;<br>(3) Moderately;<br>(4) Quite a bit;<br>(5) Extremely
These questions are about how you feel and how things have been with you during	

For peer review only

the past 4 weeks. For each question, please give the one answer that comes closest to the way you have been feeling. How much of the time during the past 4 weeks...<br>23. Did you feel full of pep?<br>*(Domain: Emotional WBeing)*<br>24. Have you been a very nervous person?<br>*(Domain: Emotional WBeing)*<br>25. Have you felt so down in the dumps that nothing could cheer you up?<br>*(Domain: Emotional WBeing)*<br>26. Have you felt calm and peaceful?<br>*(Domain: Emotional WBeing)*<br>27. Did you have a lot of energy?<br>*(Domain: Emotional WBeing)*<br>28. Have you felt downhearted and blue?<br>*(Domain: Emotional WBeing)*<br>29. Did you feel worn out?<br>*(Domain: Emotional WBeing)*<br>30. Have you been a happy person?<br>*(Domain: Emotional WBeing)*<br>31. Did you feel tired?<br>*(Domain: Emotional WBeing)*	How much of the time during the past 4 weeks...<br>23. Did you feel full of pep?<br>(1) All of the time;<br>(2) Most of the time;<br>(3) A good bit of the time;<br>(4) Some of the time;<br>(5) A little of the time;<br>(6) None of the time<br>24. Have you been a very nervous person?<br>Answer (1)-(6)<br>25. Have you felt so down in the dumps that nothing could cheer you up?<br>Answer (1)-(6)<br>26. Have you felt calm and peaceful?<br>Answer (1)-(6)<br>27. Did you have a lot of energy?<br>Answer (1)-(6)<br>28. Have you felt downhearted and blue?<br>Answer (1)-(6)<br>29. Did you feel worn out?<br>Answer (1)-(6)<br>30. Have you been a happy person?<br>Answer (1)-(6)<br>31. Did you feel tired?<br>Answer (1)-(6)
32. During the past 4 weeks, how much of the time has your physical health or emotional problems interfered with your social activities (like visiting with friends, relatives, etc.)?<br>*(Domain: Emotional WBeing)*	32. During the past 4 weeks, how much of the time has your physical health or emotional problems interfered with your social activities (like visiting with friends, relatives, etc.)?<br>(1) All of the time;<br>(2) Most of the time;<br>(3) Some of the time;<br>(4) A little of the time;<br>(5) None of the time
How TRUE or FALSE is each of the following statements for you.<br>33. I seem to get sick a little easier than other people<br>*(Domain: Phys Health)*<br>34. I am as healthy as anybody I know<br>*(Domain: Phys Health)*<br>35. I expect my health to get worse<br>*(Domain: Phys Health)*	How TRUE or FALSE is each of the following statements for you.<br>33. I seem to get sick a little easier than other people<br>(1) Definitely true;<br>(2) Mostly true;<br>(3) Don't know;<br>(4) Mostly false;<br>(5) Definitely false<br>34. I am as healthy as anybody I know<br>Answer (1)-(5)<br>35. I expect my health to get worse<br>Answer (1)-(5)

For peer review only

36. My health is excellent<br>*(Domain: Phys Health)*	36. My health is excellent<br>Answer (1)-(5)

Note that the answers to the 36 questions were recoded on a scale from 0 to 100 with 100 indicating the highest levels of health (ie negatively-phrased questions being coded in reverse polarity).

These were then divided into eight sub-domains (four physical health, four emotional well-being, with the recoded variables being summed then divided by the number of questions asked:

Domain	Physical Well-Being		Emotional Well-Being	
Sub-domain	Physical functioning	Pain	Role limitations due to emotional problems	Fatigue
	Role limitations due to physical health	General Health	Emotional Well-Being	Social Functioning

Cronbach's Alpha for the four Physical Well-Being measures is 0.838.

For the four Emotional Well-Being measures, Alpha=0.684.

For peer review only

CASP-12 (v2) Item	Wording
1-3 Control	1 My age prevents me from doing the things I would like to.<br>(1) Often; (2) Sometimes; (3) Not often; (4) Never<br>2 I feel what happens to me is out of my control.<br>Answer (1)-(4)<br>3 I feel left out of things<br>Answer (1)-(4)
4-6 Autonomy	4 I can do the things I want to do.<br>Answer (1)-(4)<br>5 Family responsibil prevent me from doing what want to do.<br>Answer (1)-(4)<br>6 I feel that I can please myself what I do.<br>Answer (1)-(4)<br>6? Shortage of money stops CM doing things wants to do.<br>Answer (1)-(4)
7-9 Self-Realisation	7 I look forward to each day.<br>Answer (1)-(4)<br>8 I feel that my life has meaning.<br>Answer (1)-(4)<br>9 I enjoy the things that I do.<br>Answer (1)-(4)<br>9? On balance I look back on life with sense of happiness.<br>Answer (1)-(4)
10-12 Pleasure	10 I feel full of energy these days.<br>Answer (1)-(4)<br>11 I feel that life is full of opportunities.<br>Answer (1)-(4)<br>12 I feel that the future looks good for me.<br>Answer (1)-(4)

Cronbach's Alpha for the twelve CASP items is 0.861.

For peer review only

Appendix 4 Rutter Behaviour scale, NCDS aged 16 (18-point version asked to mother)

Below are a series of descriptions of behaviour often shown by young people. Please ask the mother about each one and ring the appropriate number to show the degree to which this description is true of the study child:

0 Does not apply
1 Applies somewhat
2 Certainly applies

1	Restless, difficulty staying seated
2	Squirmy,fidgety child
3	Often destroys others property
4	Frequently fights, quarrelsome
5	Not much liked by other children
6	Often worries about things
7	Does things on own, rather solitary
8	Irritable, flies off the handle
9	Appears miserable,unhappy & tearful
10	Twitches,mannerisms,tics-face or body
11	Frequently sucks thumb or fingers
12	Frequently bites nails or fingers
13	Is often disobedient
14	Cannot settle more than a few moments
15	Fearful of new situations or things
16	Fussy, over particular
17	Often tells lies
18	Bullies other children

Max score=36

Cronbach's Alpha for age 16 Rutter Scale = 0.687.
Age 11 Rutter scale has 14 items; Alpha=0.682.
Age 7 Rutter scale has 14 items; Alpha=0.669.

For peer review only

Appendix 5: Calculation of Indirect Effect and Total Effects in the Structural Equation Model

A Indirect effects from early-life circumstances to Age 50 outcomes (where total effect size >.01 and statistically significant)

Effects from	To	Total effect	Total indirect	Sig. direct effect? (p<.001)	Total effect significant?
SC Birth	Age 50 Cog	0.117	0.117	No	Yes
Birthwt	Age 50 Cog	0.047	0.030	No	Yes
Rutter7	Age 50 Cog	-0.065	-0.071	No	Yes
Rutter11	Age 50 Cog	-0.038	-0.025	No	Yes
Rutter16	Age 50 Cog	-0.018	-0.023	No	Yes
Masmoke	Age 50 Cog	-0.027	-0.027	No	Yes
Breastf	Age 50 Cog	0.039	0.039	No	Yes
SC Birth	PWB	0.081	0.048	No	Yes
Birthwt	PWB	0.011	0.011	No	Yes
Breastf	PWB	0.014	0.014	No	Yes
Rutter7	PWB	-0.043	-0.059	No	Yes
Rutter11	PWB	-0.081	-0.039	No	Yes
Rutter16	PWB	-0.074	-0.002	Yes	Yes
Masmoke	PWB	-0.010	-0.010	No	Yes
Rutter7	EWB	-0.046	-0.046	No	Yes
Rutter11	EWB	-0.080	-0.061	No	Yes
Rutter16	EWB	-0.133	-0.003	Yes	Yes
SC Birth	QoL	0.055	0.033	No	Yes
Rutter7	QoL	-0.048	-0.034	No	Yes
Rutter11	QoL	-0.049	-0.049	No	Yes
Rutter16	QoL	-0.103	-0.004	Yes	Yes

B Indirect effects from early-life circumstances to early mid-life outcomes (total effect size >.01 and statistically significant)

Effects from	To	Total effect	Total indirect	Sig. direct effect? (p<.001)	Total effect significant?
SC Birth	SC42yrs	0.167	0.113	Yes	Yes
SC Birth	Quals	0.184	0.135	Yes	Yes
Birthwt	SC42yrs	0.030	0.030	No	Yes
Birthwt	Quals	0.035	0.035	No	Yes
Masmoke	SC42yrs	-0.027	-0.027	No	Yes
Masmoke	Quals	-0.032	-0.032	No	Yes
Breastf	SC42yrs	0.039	0.039	No	Yes
Breastf	Quals	0.047	0.047	No	Yes

For peer review only

C Indirect effects from early life circumstances to Age 50 outcomes (total effect size <.01 or not statistically significant)

Effects from	to	Total effect	Total indirect	Sig. direct effect? (p<.001)	Total effect significant?
SC Birth	EWB	0.031	0.012	No	No
Birthwt	EWB	0.007	0.004	No	No
Masmoke	EWB	-0.003	-0.003	No	Yes
Breastf	EWB	0.005	0.005	No	Yes
Birthwt	QoL	0.003	0.006	No	No
Masmoke	QoL	-0.005	-0.005	No	Yes
Breastf	QoL	0.008	0.008	No	Yes

Commentary: Three direct influences stand out in Table A, each involving the Rutter scores at age 16 years (see Appx 4).

These are modest direct influences on PWB and QoL, but a relatively stronger negative influence on EWB at age 50 years.

Two direct influences stand out in Table B: from social class at birth to social class at 42 years and Qualifications obtained. The direct influences are shown in our estimated pathways diagram (Fig.2), but we also see in Table C there is an additional indirect effect (via childhood cognition at ages 7/11/16), over twice as large as the direct effect in both cases.

For peer review only

Appendix 6: Separate analyses by sex

Running the analyses separately by sex, we see that certain pathways are significant for one sex but not the other (p< .001), and certain path coefficients are notably different:

Men only (N=3815)

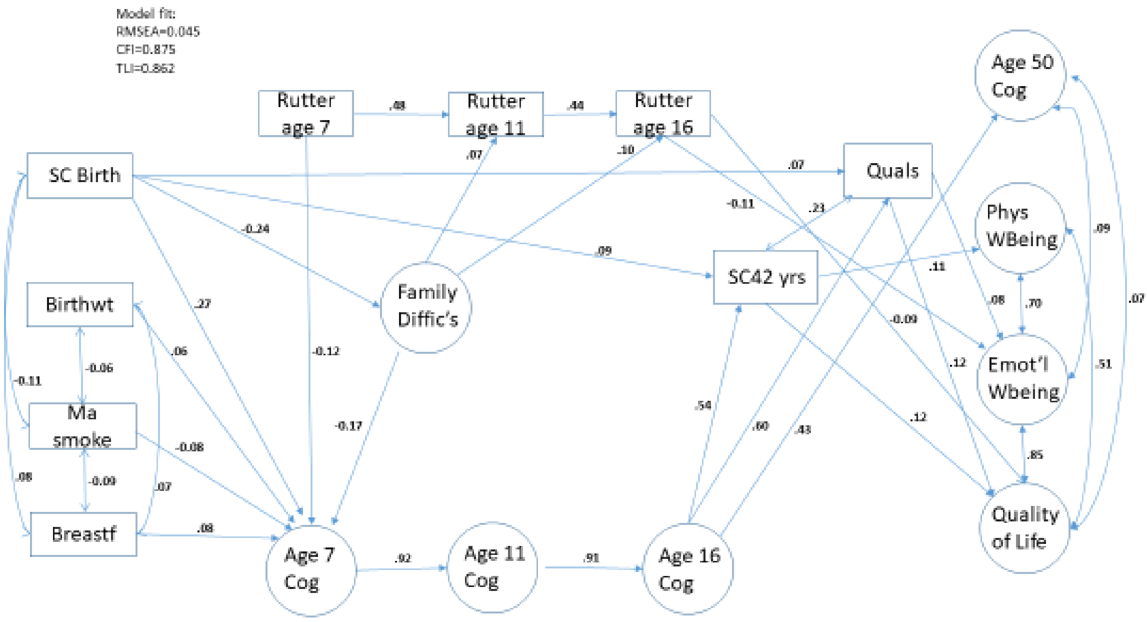

Women only (N=4209)

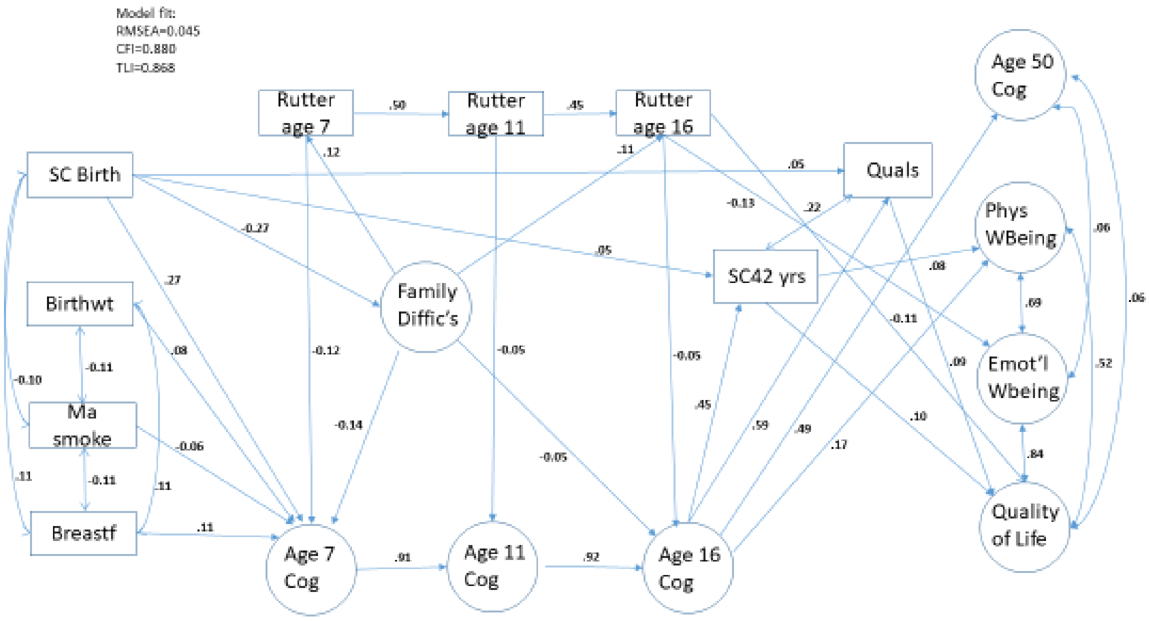

Appendix 7: Sensitivity analysis (multigroup by sex)

An MPlus multigroup run by sex was performed as a sensitivity analysis, in which each of the 50 possible paths in Fig.2 was labelled m1-m50, and (in the female model) f1-f49, then a set of 50 'difference' variables was defined diff(i)=m(i)-f(i).

Testing the significance of these 50 'diff' variables, 6 were found to be significant, and the other 43 non-significant.

The MPlus multigroup model was then re-run, constraining all the 44 non-significant paths to be equal for males and females, but allowing the other six paths to vary, thus increasing the parsimony of the model.

The six paths found to be significantly different between men and women are shown in the table below:

	Men: Estimate	P-val	Women: Estimate	P-val
Age 50 cog on SC 42 yrs	0.07	0.002	-0.03	0.146
Age 50 cog on Age 16 Cog	0.42	0.000	0.47	0.000
SC42yrs on SC Birth	0.10	0.000	0.01	0.461
Phys Health on Age 16 Cog	0.12	0.000	0.13	0.000
Quals on Age 16 Cog	0.61	0.000	0.59	0.000
Age 16 Cog on Age 11 Cog	0.91	0.000	0.92	0.000

Model fit:
RMSEA 0.047
CFI 0.861
TLI 0.855

The first three paths are notable, but in the case of the last three the difference in the point estimates, though significant, is so small as to be unremarkable.

We see that men's Age 50 cognitive ability at age 50 has a stronger link to age 42 SES than for women, where the path is not significant (strictly speaking, male p-value falls just sort of satisfying Bonferroni-corrected significance level .001, but female p-value is nowhere near).

In contrast, men's Age 50 cognitive ability has a weaker link with Age 16 cognition (.42) than for women (.47).

Finally, for men there is a significant direct link from SC birth to SC 42 years, a result not significant for women.

For peer review only

BMJ Open

Exploring the role of early life circumstances, abilities and achievements on well-being at age 50 years: evidence from the 1958 British Birth Cohort Study

Journal:	*BMJ Open*
Manuscript ID	bmjopen-2019-031416.R2
Article Type:	Original research
Date Submitted by the Author:	28-Oct-2019
Complete List of Authors:	Dodgeon, Brian; UCL Institute of Education Centre for Longitudinal Studies, Social Science<br>Patalay, Praveetha; University College London, Centre for Longitudinal Studies<br>Ploubidis, George; University College London Institute of Education, Centre for Longitudinal Studies<br>Wiggins, Richard; University College London, Centre for Longitudinal Studies
**Primary Subject Heading**:	Public health
Secondary Subject Heading:	Mental health, Research methods, Sociology, Epidemiology
Keywords:	MENTAL HEALTH, PUBLIC HEALTH, STATISTICS & RESEARCH METHODS, mid-life physical and emotional well-being

SCHOLARONE™
Manuscripts

I, the Submitting Author has the right to grant and does grant on behalf of all authors of the Work (as defined in the below author licence), an exclusive licence and/or a non-exclusive licence for contributions from authors who are: i) UK Crown employees; ii) where BMJ has agreed a CC-BY licence shall apply, and/or iii) in accordance with the terms applicable for US Federal Government officers or employees acting as part of their official duties; on a worldwide, perpetual, irrevocable, royalty-free basis to BMJ Publishing Group Ltd ("BMJ") its licensees and where the relevant Journal is co-owned by BMJ to the co-owners of the Journal, to publish the Work in this journal and any other BMJ products and to exploit all rights, as set out in our licence.

The Submitting Author accepts and understands that any supply made under these terms is made by BMJ to the Submitting Author unless you are acting as an employee on behalf of your employer or a postgraduate student of an affiliated institution which is paying any applicable article publishing charge ("APC") for Open Access articles. Where the Submitting Author wishes to make the Work available on an Open Access basis (and intends to pay the relevant APC), the terms of reuse of such Open Access shall be governed by a Creative Commons licence – details of these licences and which Creative Commons licence will apply to this Work are set out in our licence referred to above.

Other than as permitted in any relevant BMJ Author's Self Archiving Policies, I confirm this Work has not been accepted for publication elsewhere, is not being considered for publication elsewhere and does not duplicate material already published. I confirm all authors consent to publication of this Work and authorise the granting of this licence.

For peer review only

Exploring the role of early life circumstances, abilities and achievements on well-being at age 50 years: evidence from the 1958 British Birth Cohort Study

Corresponding author: Brian Dodgeon, Centre for Longitudinal Studies, UCL Institute of Education, 20 Bedford Way, London WC1H 0AL. b.dodgeon@ucl.ac.uk 020-7612-6877.
ORCID: 0000-0002-7130-8761

Co-authors:
Praveetha Patalay, Centre for Longitudinal Studies, UCL Institute of Education, 20 Bedford Way, London WC1H 0AL United Kingdom.
ORCID: 0000-0002-5341-3461

George B. Ploubidis, Centre for Longitudinal Studies, UCL Institute of Education, 20 Bedford Way, London WC1H 0AL United Kingdom.
ORCID: 0000-0002-8198-5790

Richard D. Wiggins, Centre for Longitudinal Studies, UCL Institute of Education, 20 Bedford Way, London WC1H 0AL United Kingdom.
ORCID: 0000-0007-2648-597X

Contributorship Statement

Brian Dodgeon performed the data management and structural equation modelling using the Mplus software, and took responsibility for completing all aspects of the paper.

Praveetha Patalay contributed to the conceptual planning of the analysis jointly with Richard Wiggins, performed preliminary regression modelling and advised on aspects of the structural equation modelling (SEM) strategy.

George Ploubidis advised on many aspects of the statistical analysis, and provided detailed comments on the drafting of the paper.

Richard Wiggins undertook the conceptual planning of the analysis jointly with Praveetha Patalay, and initiated the SEM approach, continuing to provide advice on the models as they became more complex, and collaborating with Brian Dodgeon on the write-up of the findings.

Word count:
3,121

ABSTRACT

1 Objectives

We examine the relative contributions of pathways from middle childhood/adolescence to mid-life well-being, health and cognition, including family socio-economic status (SES) at birth, educational achievement and early-adulthood SES. Our approach is largely exploratory, suspecting key mediators between childhood circumstances and mid-life physical & emotional well-being may be cognitive performance during school years, material & behavioural difficulties, and educational achievement. We explore whether these effects differ between men and women.

2 Setting/Participants

For peer review only

Data were the National Child Development Study, a fully-representative British Birth Cohort sample of 17,415 born in one week in 1958.

3 Primary/secondary outcome measures

Four primary mid-life outcome measures: cognitive performance; physical and emotional well-being and quality of life. Our intermediate adult outcomes are early-adulthood social class and educational/vocational qualifications.

4 Results

Using structural equation modelling, we explore pathways through childhood and early adulthood which link significantly to our outcomes. We examine the mediating effects of: cognitive ability at ages 7, 11 and 16 years; childhood psychological issues; family material difficulties at age 7: housing, unemployment, finance; educational/vocational qualifications, social class position age 42 years.

We find social class at birth has a strong indirect effect on the age 50 outcomes via its influence on cognitive performance in childhood/adolescence, educational attainment and mid-life social class position, together with small direct effects on qualifications and social class position at 42 years. Teenage cognitive performance has a strong positive effect on later physical health for women; qualifications have a stronger positive effect on emotional well-being for men.

4 Conclusion

We show the legacy of early life on multiple aspects of mid-life health, well-being, cognition and quality of life: stronger links for men from childhood social class position to adult social class position. The observed effect of qualifications supports those arguing that education associates positively with subsequent cognitive functioning.

Article Summary

Strengths and limitations of this study:

- The richness of its longitudinal dimension, conveying the importance of early life circumstances upon early adult and mid-life measures of achievement, health and well-being;
- The integrated nature of the analysis simultaneously models pathways to four mid-life outcomes, three of which are strongly correlated after the inclusion of several key early-life antecedents;
- It makes optimal use of all the available longitudinal data by employing Full Information Maximum Likelihood estimation to take account of item non-response;
- It shows the enduring importance of parental social class for cognitive performance and later life outcomes, controlling for family material difficulties in childhood.
- A potential limitation is that our model does not include a broader set of demographic and psychosocial factors: a result of our decision to restrict the number of early-life influences to avoid over-complexity in the statistical modelling. There is also a small amount of attrition bias between birth and age 50.

For peer review only

INTRODUCTION

Authors since the 1990s have advanced the importance of a life course approach to explore the relationship between early life circumstances and later life health outcomes [1, 2]. Carr [3] emphasises how diverse early life experiences affect physical, emotional and cognitive health in later life by drawing upon a wealth of longitudinal data across the United States, Australia and Europe. Our empirical study is based on a single British Birth Cohort which covers several waves of data collection from birth (1958), middle childhood (age 7 & 11), adolescence (age 16) and mid-life at ages 42 and 50 years for over 8,000 sample members. Age 50 years represents a specific mid-point of mid-life (40-60) as defined by Midlife Development in the United States (MIDUS): Brim et al [4] suggest “Midlife has been described as the last uncharted territory of the life course”. To this extent our conceptual framework is largely informed by Elder [5]: it may be described as a cumulative (dis-) advantage model [6] where we examine the legacy of early life circumstances, notably the British Registrar General’s classification of occupations (RG social class) [7] as having lasting effects on health and well-being at age 50 years. Our approach implies a reciprocal relationship between socio-economic status (SES) and health, and allows for the possibility that favourable circumstances in later life can act counter to the effects of earlier disadvantage [8].

To this end we select outcomes at age 50 years covering four distinct areas: self-assessed quality of life, physical and emotional well-being, and a cognitive ability test.

Our research interest is in exploring the extent to which these outcomes inter-relate, adopting an empirical view that, taken together, these measures would tell us more about the consequences of the past than studied individually [9]. There is a growing body of evidence based on United Kingdom (UK) longitudinal studies [10,11,12] that provides a strong argument for looking at these outcomes together within the same model. Cooper et al [13] provided both substantive and empirical evidence for examining gender differences in this analysis.

Drawn from the same rich longitudinal resource, our predictors and mediators begin with birth circumstances: birthweight, breastfeeding, maternal smoking during pregnancy and parental SES. From the age 7 sweep of the study we include the effects of family material difficulties such as unemployment, finance and housing, in conjunction with childhood scores of socio-emotional adjustment and cognitive performance, each measured on three occasions spanning the ages 7, 11 and 16 years. The inclusion of highest qualification and SES at age 42 in the model enables us to trace which childhood effects persist, and which are built upon or attenuated by education and social mobility.

Our analysis builds upon the framework adopted by Wood et al [14] on the prediction of mental well-being as a single outcome [15] across four UK birth cohort studies, which showed that childhood SES is directly and indirectly (through adult socioeconomic pathways) linked to adult emotional well-being. We expand upon their approach by including four outcome measures, albeit for a single birth cohort.

Our analysis adopts a structural equation modelling (SEM) approach [16], in order to explore the pathways from childhood and adolescence to our multivariate mid-life outcomes for both men and women.

For peer review only

DATA AND METHODS

Our data source, the British National Child Development Study (NCDS) is fully representative of the population of Great Britain (GB), having interviewed the parents of 98.5% of all babies born in the same week of 1958 [17], and followed them up longitudinally with ten subsequent interviews throughout life. It provides operational measures regarding birth circumstances, the early and teen years covering ages 7, 11 and 16, and adulthood at ages 42 and 50 respectively.

The analysis is based upon 3815 male and 4209 female cohort members present at each occasion who all have at least one genuine response for each of the four separate age 50 life domains, and who also participated in at least one of the three cognitive and socio-emotional assessments, in order to permit a reliable strategy for handling missing items.

We are aware there is a certain amount of attrition bias as a result of differential loss-to follow-up from the 17,415 cohort members present at the birth study in 1958. As a check on the impact of sample loss, we were reassured by the close match of the distribution of the birth social class variable within our sample of 8,024 against that of the same variable within the full 17,415 birth sample (chi-square=1.29, 5d.f., non-significant) (see Appendix 1).

Our outcomes at age 50 years cover four distinct measures, the first two being self-reported physical and emotional well-being (PWB and EWB respectively) measured using sub-scales [18] of the RAND 36-Item Short-Form Health Survey [19-23] (hereafter SF-36). Third, a self-reported measure of quality of life (QoL) covering the life domains of 'Control, Autonomy, Self-Realisation and Pleasure (CASP-12 v2) [24]: a theoretically-informed measure of subjective well-being, closely following the approach adopted by Ed Diener and his colleagues [25]. The fourth domain outcome, cognitive ability (hereafter Age50Cog) consists of four cognitive tests examining memory (word recall & delayed recall), executive function (animal naming), attention and mental speed (letter cancellation) [26].

Appendix 2 lists all of the manifest and latent variables employed in the analysis. Indicators for cognitive function at age 50 years are listed here, while those for physical, emotional and quality of life outcomes are expanded upon more fully in Appendix 3. Childhood behavioural indicators are listed in Appendix 4 following Rutter [27]. We recognise of course that Rutter scores may be sub-divided into constituent dimensions of problem behaviour (withdrawn/ anxious / oppositional/ inattentive [28], but for this paper we prefer to use Rutter total scores, following Parsons et al [29].

In fitting our structural equation models using the MPlus software [30] we obtain direct and indirect (mediated) effects of our background variables in terms of childhood social origins and social adjustment, on outcomes in early adulthood, and mid-life at age 50 years. We regard all four of our birth indicators as primary predictors, valuing the opportunity to assess the relative impact of each for policy interventions [31]. Our birth social class variable is based on father's occupation, since in 1958 when the cohort were born, only 38% of mothers were economically active.

The sets of predictors determining our paths differ slightly for each of the four outcomes so as to avoid the problem of 'seemingly unrelated regression' [32]

For peer review only

We employ full information maximum likelihood estimation (FIML) [33]. This addresses the issue of item non-response arising where questions are avoided or not answered by certain participants.

The SEM procedure that follows begins with an analysis of the sample as a whole, followed by separate analyses for men and women, supported by a multigroup analysis [34] to test for the structural equivalence of the models for men and women. Model 'goodness of fit' is assessed via conventional criteria: 'root mean square error' (RMSEA), 'comparative fit index', (CFI) and 'Tucker Lewis fit index', (TLI) where RMSEA<0.05, CFI>0.90 and TFI> 0.90 would provide evidence of 'good fit' [35].

Having a large number of SEM pathways involving 261 free parameters, we had regard to the n:q rule [36] and were satisfied that our sample-size-to-parameters ratio was comfortably within Jackson's guideline of 10:1. Our application of SEM involved the use of modification indices, which led to our running correlations on suggested variables. All our final models were identified, all manifest variables loading onto the respective latent variables with satisfactory fit. The details of the individual assessments of our measurement models appear in Appendix 5.

Patient and Public involvement statement

Participant consent was obtained from parents of cohort members at the initiation of the original birth cohort study in 1958. On attaining adulthood, permission was obtained from cohort members themselves at each subsequent longitudinal sweep.

RESULTS

The results begin with consideration of the inter-correlations between our mid-life outcome measures. Further univariate descriptives are contained in Appendix 2. Figure 1 contains a final SEM diagram for the sample as a whole. Subsequent reference will be made to Appendix 6 for the details of mediation, total and indirect effects. Appendix 7 contains separate analyses by sex, and Appendix 8 contains further information on our multigroup analysis based on gender.

(a) Descriptives & correlations

As a preliminary inspection of the inter-relationships between our four outcome measures, Table 1 contains a correlation matrix of the four summative indices based on these measures for both men and women.

Table 1. Associations between the four sets of mid-life outcomes as summative indices (Men/Women)

Age 50 Domain	Age 50 Cog	PWB	EWB	QoL
Cognitive Ability (Age 50 Cog)	1.00/1.00			
Physical Well-Being (PWB)	0.12/0.15	1.00/1.00		
Emotional Well-Being (EWB)	0.12/0.09	0.57/0.58	1.00/1.00	

For peer review only - http://bmjopen.bmj.com/site/about/guidelines.xhtml

Quality of Life (QoL)	0.15/0.10	0.48/0.48	0.63/0.64	1.00/1.00

Interestingly, QoL, PWB and EWB demonstrate moderate associations of inter-correlation. While cognitive ability is positively related to the physical and emotional well-being measures, the strength of the association is modest, with PWB having a higher coefficient for women and EWB & QoL a higher coefficient for men.

Fig. 1 contains estimates of association amongst these outcomes as defined by measurement models under a full SEM analysis. As a check on the robustness of our statistical findings the SEM analyses were subject to bootstrap analysis under 1,000 repetitions. We also carried out a check on the influence of outliers (results available on request).

(b) Birth social class effects on early mid-life outcomes

The path diagram shown in Fig 1 provides the model results for the sample as a whole showing only statistically significant paths at a p-level of .001, employing a Bonferroni correction for the fifty path coefficients being estimated (0.05/50=0.001) [37].

Fig 1. Estimated pathways of the Structural Equation Model, N=8024 (showing paths significant at p < .001)

[insert Fig.1 here]

Whilst there are various pathways that connect early life circumstances with cognitive performance, family material difficulties and social adjustment, there remains a small direct effect of social class at birth on each of our two early adult outcomes: Age 42 Social class and educational/vocational qualifications (β=0.05 for both paths). These two 'interim' or 'pivotal' destinations, in turn, have notable effects on our four mid-life outcomes at age 50, which we describe under (d) below.

(c) Indirect effects via pathways through childhood mediators

Inspecting the paths in Fig.1 from birth circumstances through ages 7, 11 and 16, we see firstly, that all four of our birth characteristics have direct effects on cognitive performance at age 7 years, when children are in primary school. Social class at birth has the most notable association with age 7 cognition (β=.27). This cognitive variable in turn has a strong direct effect on age 11 cognition (β=.92) and similarly for age 11 on cognitive performance at age 16 (β=.92).

There is a clear path via cognitive ability throughout childhood and adolescence, to adult achievement as indicated by Age 42 social class and educational qualifications (Age 16 Cog to Social class 42yrs β=.50, Age 16 Cog to Quals β=.59).

This legacy of childhood cognitive performance is unsurprising, but interestingly Age 16 cognition also has a direct association with age 50 physical well-being (β=.12) besides more naturally, Age 50 cognition (β=.47).

Family material difficulties in childhood, themselves associated with birth social class (β=-0.26), also play their part in understanding a child's trajectory in cognitive performance. Characterised by

For peer review only - http://bmjopen.bmj.com/site/about/guidelines.xhtml

issues with housing, finance and employment when a child is 7 years old, these family material difficulties are seen to have negative consequences for cognitive performance at age 7 years (β=-.15) and a lagged negative effect on later performance age 16 years (β=-0.04).

Additional analyses also show that family material difficulties impact on a child's emotional and behavioural scores (based on Rutter) at ages 7, 11 and 16 (β=.09, .06 and .10 respectively). In turn these scores had small significant negative correlations with cognitive performance at each corresponding age (-.15, -.11 and -.11 respectively). But most notably, Rutter scores at these three ages are significantly correlated (.49 for age 7 to 11 and .44 for age 11 to 16), and there are direct effects from age 16 Rutter scores to three of our four age 50 outcomes: EWB (β=-0.13), QoL (β= -0.10) and PWB (β=-0.07).

(d) Indirect effects via adulthood mediators

Adult social class and lifetime qualifications are, together, influential predictors of each of our four mid-life outcomes: adult social class has significant predictive effects (β=.08 and .11) on PWB and QoL.

In general, the socially-advantaged enjoy better health and report higher levels of quality of life than their less advantaged counterparts. Interestingly, qualifications predict age 50 cognitive ability (β=.05), EWB (β=.06) and QoL (β=.10) but not PWB.

(e) Gender differences

Our multigroup analysis reveals that certain paths have significantly different effects for men and women. In Appendix 7 we display two separate path diagrams for men and women. For men, there is a direct effect of social class at birth on Age 42 social class and qualifications (β=.09 and .07 respectively), but for women there is no significant effect.

Both sexes see the birth social class effect remain equally strong for cognitive performance at age 7 years and beyond, but women experience a slightly worse negative effect of social disadvantage at birth on the latent variable 'family material difficulties' (β=-0.27 compared to -0.24 for men). In turn, family material difficulties have slightly more influence on Age 7 cognition (β=-.18) for men than for women (β=-.16), and a small lagged effect on Age 16 cognition only evident for women (β=-.05). The Rutter social adjustment scores at age 11 do not significantly correlate with the cognitive performance scores for men, but they do for women at ages 7, 11 and 16 (-.15, -.11 and -.13 compared with men's -.14 at age 7, -.11 at age 16).

Early adult social class has a slightly stronger influence for men than women on our age 50 outcomes PWB and QoL, and Qualifications have a significant effect on EWB for men, but not for women. In contrast, women's age 16 cognition has a significant effect on their later PWB (β=.17), an effect not significant for men; and the path from age 16 cognition to age 50 Cog has a stronger coefficient for women (β=.49) than men (β=.43).

We pursue the evidence for gender differences via a sensitivity analysis under MPlus which formally tests for statistical difference between paths for each sex. Appendix 8 identifies six paths where the

For peer review only

effects for men and women are significantly different, but only three have notable differences in terms of their effect sizes in the multigroup analysis. These are:

- stronger effect for women from age 16 cognition to age 50 cognition as noted above (β=.49 to .43);
- direct effect of social class at birth on Age 42 social class (β=.09 for men, no significant effect for women); and
- link for women from Age16 Cognition to Phys WBeing (β= .17)(no significant effect for men)

DISCUSSION

Structural equation modelling has elucidated the nuances of the influence of pathways from childhood and early adulthood to our four related outcomes of health, well-being and cognition in mid-life. The work demonstrates the benefits of an integrated approach to defining the aspects of the life course that affect well-being in mid-life. Physical and emotional health are important factors affecting a person's subjective account of their quality of life, and PWB correlates at .69 with EWB, which in turn correlates .83 with QoL. While cognitive performance at age 50 is relatively weakly correlated, the other three are closely intertwined. These associations are similar in magnitude to those reported in table 1; however they are now estimated by taking measurement error and the legacy of the life course into account.

We also show that some of the paths to health and well-being in mid-life are different for men and women [38]. However, important communalities remain.

Social origins, in particular social class at birth, have a major indirect influence on mid-life outcomes, the mediation process beginning with a child's cognitive performance throughout formal schooling and subsequently via cognitive performance at age 16 years. Collectively, these influences are weakened or reinforced by the existence of family material difficulties in early childhood (themselves influenced by childhood social class). Whilst cognitive performance is a strong predictor of early adult social class and educational status for both men and women, social class at birth also has a lasting association with these mid-life indicators for men but not for women.

This could well suggest that women have a looser connection with the legacy of social class at birth, perhaps partly because their path to advantaged SES at age 42 may be impeded by caring responsibilities and gender inequalities in the labour market [39].

Early adult social class influences all four of our mid-life outcomes, but we see the direct effect of social class at birth on social class and qualifications in early adulthood is pronounced for men (β=.09 and .07 respectively), but not significant for women.

A relatively small but consistent direct effect of qualifications on Age 50 Cognition supports those arguing that education is positively associated with subsequent cognitive functioning [40], although we are aware that there are counter-arguments to this view [41]). Equally, the relationship between QoL and early adult social class finds support in the work of Blane et al [42].

The strength in the evidence presented here lies in the richness of its longitudinal dimension which conveys the importance of early life circumstances upon early adult and mid-life measures of achievement, health and well-being.

For peer review only

One limitation of our study is that our model does not fully take into account all of the potential demographic and psychosocial factors other researchers have studied as predictors of satisfactory midlife development [28]. Nevertheless, it can be argued that our birth social class variable acts as a 'proxy' for certain parental beliefs and behaviours [43]. We are embarking on developing a conceptual framework and modelling strategy to extend our empirical analyses in the future as our cohort members age: in particular, our subjects are not yet old enough for us to assess whether any decline in cognitive performance at age 50 years will lead to subsequent decline and become more influential on emotional well-being and quality of life. However, we were able to show that cognitive performance in the teen years holds a legacy for cognitive performance in mid-life. A final limitation is a certain amount of attrition bias between birth and age 50 (differential non-response) which was addressed earlier in the paper and in Appendix 1.

Social class at birth has a strong influence on early cognitive performance and beyond, which in turn has consequences for adult achievement and social class. Clearly, the presence of family material difficulties and behavioural issues will impede (mediate) this trajectory. Interestingly, childhood behaviour, in particular at age 16, has a lasting effect on mid-life PWB, EWB and Quality of Life.

In sum, health, emotion, quality of life and to a lesser extent cognitive function do not stand alone as markers of well-being in mid-life. They represent the constituents of an inter-related whole which is shaped by early life circumstances, family material difficulties, social adjustment and cognitive performance in childhood.

Acknowledgements

We would like to acknowledge the thoughtful and constructive comments of our three reviewers, which strengthened our paper.

References

1 Williams, D.R. (1990). Socioeconomic differentials in health: A review and redirection. Social Psychology Quarterly 53, 81–99.

2 Wadsworth, M. E. J. (1997). Health inequalities in the life course perspective. Social Science & Medicine 44, 859–869.

3 Carr, D (2019). Early-life influences on later life well-being: innovations and explorations. The Journals of Gerontology, Series B: Psychological Sciences and Social Sciences, 74(5), 829-831.

4 Brim, O, Ryff, G, Kessler, C.D & Ronald, C (2004). How healthy are we? A National Study of Well-Being at Midlife. Chicago, IL: University of Chicago Press.

5 Elder, G (1994). Time, human agency, and social change: perspectives on the life course. Social Psychology Quarterly, 57, 4-15.

6 Dannefer, D (2003) Cumulative advantage/disadvantage and the life course: Cross-fertilizing age and social science theory. The Journals of Gerontology, Series B, 58(6), S327-S337.

7 Szreter, S.R.S (1984). The Genesis of the Registrar-General's Social Classification of Occupations. British Journal of Sociology 35(4), 522-54

For peer review only

8 Graham, H. (2002). Building an inter-disciplinary science of health inequalities: The example of lifecourse research. Social Science & Medicine 55(11), 2005–2016.

9 Marmot, M. G, Fuhrer, R, Ettner, S.L, Marks, N.F, Bumpass, L.L and Ryff, C.D (1998) Contribution of psychosocial factors to socioeconomic differences in health. Millbank Quarterly 76, 403-48.

10 Gale, C. R, Cooper, R, Craig, L, Elliott, J, Kuh, D, Richards, M & Deary, I. J (2012). Cognitive function in childhood and lifetime cognitive change in relation to mental wellbeing in four cohorts of older people. *PLoS ONE*, *7*(9), e44860. doi: 10.1371/journal.pone.0044860

11 Clouston, S. A, Brewster, P, Kuh, D, Richards, M, Cooper, R, Hardy, R, Rubin, M.S and Hofer, S M (2013). The Dynamic Relationship Between Physical Function and Cognition in Longitudinal Aging Cohorts. *Epidemiologic Reviews* 35(1), 33–50 doi: 10.1093/epirev

12 Cooper, R, Stafford, M, Hardy, R, Aihie Sayer, A, Ben-Shlomo, Y, Cooper, C & the HALCyon Study Team (2014). Physical capability and subsequent positive mental wellbeing in older people: findings from five HALCyon cohorts. *Age 36*(1), 445-456. doi: 10.1007/s11357-013-9553-8

13 Cooper, R, Hardy, R, Aihie Sayer, A, Ben-Shlomo, Y, Birnie, K. C, Craig, L, Deary, I.J, Demakakos, P, Gallacher, J, McNeill, G, Martin, R.M, Starr, J.M, Steptoe, A, Kuh, D and the HALCyon Study Team (2011). Age and Gender Differences in Physical Capability Levels from Mid-Life Onwards: The Harmonisation and Meta-Analysis of Data from Eight UK Cohort Studies. ***PLoS One***, 6(11), e27899.

14 Wood, N, Bann, D, Hardy et al (2017) Childhood socioeconomic position and adult mental wellbeing: evidence from British birth cohort studies. PLoS One, 10, e0185798.

15 Tennant, R, Hiller, L, Fishwick, R et al (2007) The Warwick-Edinburgh Mental Well-Bing Scale (WEMWBS): development and UK validation. Health and Quality of Life Outcomes 5(1), 63.

16 Kline, R.B. (1998). *Principles and Practice of structural equation modelling*. New York. Guildford Press.

17 Power, C and Elliott, J. (2006) Cohort Profile: 1958 British birth cohort (National Child Development Study). International Journal of Epidemiology 35(1), 34-41.

18 Doll, H.A, Petersen, S.E.K and Stewart-Brown, S.L (2012) Obesity and Physical and Emotional Well-Being: Associations between Body Mass Index, Chronic Illness, and the Physical and Mental Components of the SF-36 Questionnaire. Obesity 8(2), 160-170 doi: 10.1038/oby.2000.17

19 Ware, J.E and Donald Sherbourne, C (1992). The MOS 36-Item Short-Form Health Survey (SF-36): I. Conceptual Framework and Item Selection. Medical Care 30(6), 473-483.

20 Brazier, J.E, Harper, R, Jones et al (1992). Validating the SF-36 health survey questionnaire: new outcome measure for primary care. British Medical Journal 305(6846): 160-164.

21 Jenkinson, C., Wright, L. and Coulter, A. (1994). Criterion validity and reliability of the SF-36 in a population sample. Quality of Life Research 3(1), 7-12.

22 Burnholt, V & Nash, P. (2011). Short Form 36 (SF-36) Health Survey Questionnaire: normative data for Wales. Journal of Public Health, 33(4), 587-683.

23 Mishra, G.D, Gale, C.R, Aihie Sayer, A, Cooper, C, Dennison, E. M, Whalley, L.J, Craig, L, Kuh, D, Deary, I. J and the HALCyon Study Team (2011) How useful are the SF-36 sub-scales in older people? Mokken scaling of data from the HALCyon programme. *Quality of Life Research*, 20 (7), 1005-1010. (doi:10.1007/s11136-010-9838-7). (PMID:21225350)

24 Wiggins, R.D, Netuveli, G., Hyde et al (2008). The evaluation of a self-enumerated scale of quality of life (CASP-19) in the context of research on ageing: a combination of exploratory and confirmatory approaches. Social Indicators Research 89(1), 61-77.

25 Diener, E and Suh, E (1997) Measuring quality of life: Economic, social, and subjective indicators Social indicators Research, 40 (1-2), 189-216.

26 Brown, M and Dodgeon, B. (2010) NCDS Cognitive Assessments at Age 50: Initial Results. CLS Working Paper 2010/1. London: Centre for Longitudinal Studies. UCL.

27 Rutter, M, Tizard, J and Whitmore, K (1970) Education, Health & Behaviour, Longman.

28 Caspi, A, Henry, B, McGee, R.O, Moffitt, T.E & Silva, P (1995). Temperamental Origins of Child and Adolescent Behavior Problems: From Age Three to Age Fifteen Child Development 66 (1), 55-68 doi: 10.1111/j.1467-8624.1995.tb00855.x

29 Parsons, S, Green, F, Ploubidis, G.B, Sullivan, A and Wiggins, R.D (2017). The influence of private primary schooling on children's learning: Evidence from three generations of children living in the UK. British Educational Research Journal 43(5), 823-847 (doi: 10.1002/berj.3300)

30 Muthen, B.O & Muthen L.K (2018) Statistical Analysis with Latent Variables: MPlus v8.1 User Manual. Los Angeles CA: Muthen & Muthen.

31 Sacker, A, Kelly, Y, Iacovou, M, Cable, N and Bartley, M. (2013) Breast feeding and intergenerational social mobility: what are the mechanisms? ***Archives of Disease in Childhood***, 98(9), 666-671.

32 Zellner, A. 1962. An efficient method of estimating seemingly unrelated regression equations and tests for aggregation bias. Journal of the American Statistical Association 57: 348–368.

33 Arbuckle, J.L. (1996). Full Information estimation in the presence of incomplete data. In Marcoulides, G.A & & Schumaker, R.E (eds) *Advanced Structural Equation Modeling: Issues and Techniques.* Mahwah, N.J.: Erlbaum Associates.

34 Byrne, B.M. (2012). *Structural Equation Modeling with Mplus*. Routledge Taylor & Francis Group. New York & London

35 Bollen, K.A. & Scott Long, J. (eds) (1993). *Testing Structural Equation Models*. SAGE Focus edition. Newbury Park, London & New Delhi.

For peer review only

36 Jackson, D,L (2003) Revisiting Sample Size and Number of Parameter Estimates: Some Support for the N:q Hypothesis. Structural Equation Modeling 10(1), 128-141.

37 Weisstein, Eric W (2004) "Bonferroni Correction." From MathWorld--A Wolfram Web Resource. http://mathworld.wolfram.com/BonferroniCorrection.html

38 Arber, S. & Cooper, H. (1999). Gender differences in health in later life: the new paradox? Social Science and Medicine, 48(1), 61-76

39 Kahn, J.R, Garcia-Manglano, J and Bianchi, S.M (2014) The Motherhood Penalty at Midlife: Long-Term Effects of Children on Women's Careers. Journal of Marriage and the Family 76(1), 56-72.

40 Richards, M and Sacker, A (2010) Is education causal? Yes. International Journal of Epidemiology 40(2), 516–518.

41 Deary, I.J and Johnson, W (2010) Intelligence and education: causal perceptions drive analytic processes and therefore conclusions. International Journal of Epidemiology 39(5), 1362-1369.

42 Blane, D, Netuveli, G and Bartley, M (2007). Does quality of life at older ages vary with socio-economic position? Sociology **41**(4), 717-726.

43 Davis-Kean, P.E (2005). The Influence of Parent Education and Family Income on Child Achievement: The Indirect Role of Parental Expectations and the Home Environment. Journal of Family Psychology, 19(2), 294-304.

Footnotes

Funding

Assistance is acknowledged from ESRC grants ES/N003683/1 and ES/M008584/1.

Consent of authors

I, Brian Dodgeon, the Submitting Author, have the right to grant and do grant on behalf of all authors of the Work (as defined in the below author licence), an exclusive licence and/or a non-exclusive licence for contributions from authors who are: i) UK Crown employees; ii) where BMJ has agreed a CC-BY licence shall apply, and/or iii) in accordance with the terms applicable for US Federal Government officers or employees acting as part of their official duties; on a worldwide, perpetual, irrevocable, royalty-free basis to BMJ Publishing Group Ltd ("BMJ") its licensees and where the relevant Journal is co-owned by BMJ to the co-owners of the Journal, to publish the Work in BMJ Open and any other BMJ products and to exploit all rights, as set out in our licence.

The Submitting Author accepts and understands that any supply made under these terms is made by BMJ to the Submitting Author unless you are acting as an employee on behalf of your employer or a postgraduate student of an affiliated institution which is paying any applicable article publishing charge ("APC") for Open Access articles. Where the Submitting Author wishes to make the Work available on an Open Access basis (and intends to pay the relevant APC), the terms of reuse of such

Open Access shall be governed by a Creative Commons licence – details of these licences and which Creative Commons licence will apply to this Work are set out in our licence referred to above.

Competing interests

All four authors declare they have no competing interests and no financial relationships with any organisation that might have an interest in the submitted work in the previous three years, and no other relationships or activities that could appear to have influenced the submitted work.

Data Sharing statement

The analysis is based on secondary data available for download from the UK Data Archive at https://beta.ukdataservice.ac.uk/datacatalogue/series/series?id=2000032. Syntax used to derive variables for this analysis can be obtained by e-mailing the corresponding author, Brian Dodgeon.

Ethics

Ethical approval was not required, since the study used the secondary data of the National Child Development Study, which itself had received ethics consent from the London Research Ethics Committee, ref 08/H0718/29 for its age 50 survey, the source of our outcome data. Multicentre Research Ethics Committee (MREC) approval was received for earlier NCDS surveys.

For peer review only

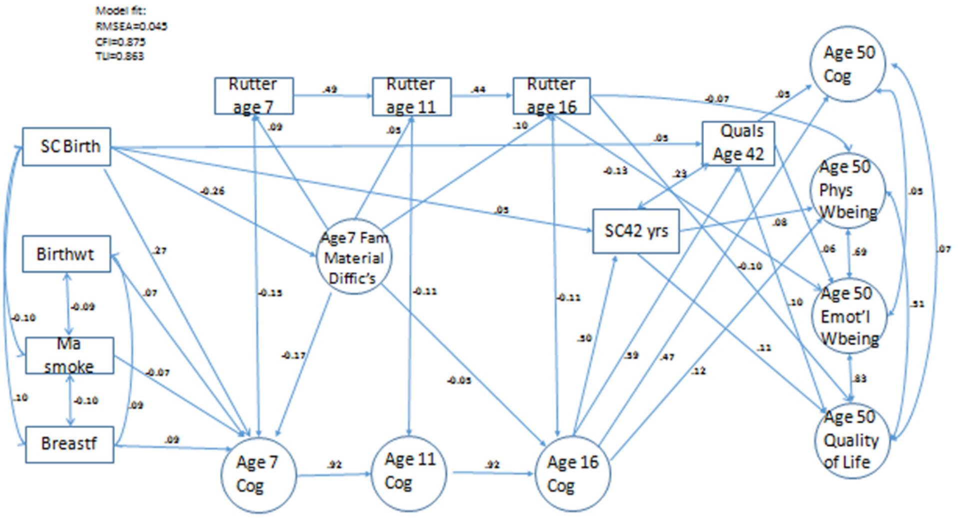

Fig 1. Estimated pathways of the Structural Equation Model, N=8024

Appendix 1

Check for attrition bias between full NCDS sample at birth and Analysis sample

Comparison of the distributions of the social class at birth variable for the original birth cohort compared to the sample used for the current analysis

Father's social class at birth	Full birth survey N=17,415	Valid %	Analysis sample N=8024	Valid %
Class I (Professional)	746	4.5	386	5.2
Class II (Intermediate)	2133	13.0	1127	15.1
Class III (Skilled Manual/Non-Manual)	9981	60.6	4498	60.1
Class IV (Semi-Skilled Manual)	1995	12.1	871	11.6
Class V (Unskilled Manual)	1616	9.8	603	8.1
Total (valid Sclass)	16471	100.0	7485	100.0
Insufficient data on parental Social Class	944		539	
Total	17415		8024	

(chi-square=1.29, 5d.f., non-significant)

For peer review only

Appendix 2: Manifest variables, age of cohort member at survey sweep, latent variable labels

Variable	Survey Sweep	Range of recorded codes	Mean	S.D.	% missing	Latent Variable
Early years						
Social class of father (GRO 1951)	Birth	1-6 (1=low)	2.98	0.92	6.7	
Birthweight	Birth	1 <2.5 kg<br>2 >=2.5kg	1.94	0.23	2.8	
Did mother smoke after 4th month of pregnancy	Birth	1-4(No/Variable Med'm/Heavy)	1.7	1.10	3.6	
Sex	Birth	1,2	52.5% female		0	
Mother breastfed child	Age 7	1 No; 2 <1 mth; 3 >1 month	2.15	0.86	10.4	
Problem arithmetic score	Age 7	0-10	5.38	2.43	9.1	Age7cog
Copying designs test age 7	Age 7	0-12	7.19	1.93	9.1	
Draw-A-Man test score	Age 7	0-51	24.40	6.95	10.6	
Southgate reading test score	Age 7	0-30	24.43	6.36	8.9	
Fam difficulties by age 7: Housing (health visitor assess't)	Age 7	0 No; 1 D/K 2Yes	0.17	0.51	9.9	Family Material Difficult's Age 7
Fam difficulties by age 7: Finance	Age 7	0,1,2	0.21	0.53	10.0	
Fam difficulties by age 7: Unempl	Age 7	0,1,2	0.11	0.38	10.0	
NFER general ability test	Age 11	0-80	46.14	15.2	12.0	Age11cog
Reading Comprehension score	Age 11	0-40	16.97	5.95	12.0	
Maths score	Age 11	0-40	18.40	10.1	12.0	
Copying designs test age 11	Age 11	0-12	8.45	1.40	12.2	
Rutter behavioural score (age 7) (mother assess't at each age)	Age 7	0-28	6.13	3.50	9.7	
Rutter behavioural scor (age 11)	Age 11	0-28	6.13	3.41	12.8	
Rutter behavioural score (age 16	Age 16	0-36	3.91	3.42	24.4	
Adolescence						
Reading Comprehension age 16	Age 16	0-40	26.58	6.23	23.1	Age16cog
Maths test age 16	Age 16	0-40	13.83	6.96	23.4	
Teacher assess't English ability	Age 16	1-5	3.44	1.12	22.9	
Teacher assess't Maths ability	Age 16	1-5	3.11	1.19	23.5	
Early Mid-Life						
Social class at age 42	Age 42	1-6 (1=low)	3.99	1.24	13.9	
Highest qual'n (acad or vocat) achieved by age 42	Age 42	0-5 (0=no quals; 1-5=NVQ)	2.68	1.36	0	
Later Mid-Life						
Latter cancellation speed	Age 50	0-800	334.60	87.8	3.5	Age50cog
Word recall	Age 50	0-10	6.60	1.46	2.1	
Delayed word recall	Age 50	0-10	5.49	1.81	2.7	
Animal naming test	Age 50	0-65	22.53	6.22	2.1	
SF-36 phys. health (4 scores: see Appx.3. Summative stats shown)	Age 50	0-100 (each of 4 scores)	315.4	83.8	0.2	PhysWbeing Age 50
SF-36 mental health (4 scores)	Age 50	0-100 (4 scores)	276.6	63.3	0.3	Emot'l Wbeing Age 50
CASP-12 (3 control scores, 3 autonomy, 3 pleasure, 3 self-realisation – see Appx.3)	Age 50	0,1,2,3 (each of 12 scores)	23.94	5.42	0.7	Quality of Life Age 50

For peer review only

Appendix 3. SF-36 scale item number, response options and wording by Model Domain, and Quality of Life based on CASP-12 (v2) items

Physical Health Domain (contains 4 sub-domains)

(a) Sub-domain 'General Health' (6 items, self-assessed general health)

1. In general, would you say your health is: (1) Excellent; (2) Very good; (3) Good; (4) Fair; (5) Poor
2. Compared to one year ago, how would you rate your health in general now? (1) Much better now than one year ago; (2) Somewhat better now than 1 year ago; (3) About the same; (4) Somewhat worse now than 1 year ago; (5) Much worse now than one year ago

How TRUE or FALSE is each of the following four statements for you.

33. I seem to get sick a little easier than other people
34. I am as healthy as anybody I know
35. I expect my health to get worse
36. My health is excellent

(b) Sub-domain 'Physical functioning' (10 items)

The following items are about activities you might do during a typical day. Does your health now limit you in these activities? If so, how much? (1) Yes, limited a lot; (2) Yes, limited a little; (3) No, not limited at all.

3. Vigorous activities, such as running, lifting heavy objects, participating in strenuous sports
4. Moderate activities, such as moving a table, pushing a vacuum cleaner, bowling, playing golf?
5. Lifting or carrying groceries?
6. Climbing several flights of stairs?
7. Climbing one flight of stairs?
8. Bending, kneeling, or stooping?
9. Walking more than a mile?
10. Walking several blocks?
11. Walking one block?
12. Bathing or dressing yourself?

(c) Sub-domain 'Role limitations due to physical health' (4 items)

During the past 4 weeks, have you had any of the following problems with your work or other regular daily activities as a result of your physical health?

13. Cut down the amount of time you spent on work or other activities?
14. Accomplished less than you would like
15. Were limited in the kind of work or other activities
16. Had difficulty performing the work or other activities

(d) Sub-domain 'Pain' (10 items)

21. How much bodily pain have you had during the past 4 weeks?
    (1) None; (2) Very mild; (3) Mild; (4) Moderate; (5) Severe; (6) Very severe
22. During the past 4 weeks, how much did pain interfere with your normal work (including work outside the home)?

For peer review only

Emotional Well-Being Domain (contains 4 sub-domains)

(a) Sub-domain 'Role limitations due to physical health' (4 items)

During the past 4 weeks, have you had any of the following problems with your work or other regular daily activities as a result of any emotional problems (such as feeling depressed or anxious)?

17. Cut down the amount of time you spent on work or other activities
18. Accomplished less than you would like
19. Didn't do work or other activities as carefully as usual

(b) Sub-domain 'Social functioning' (2 items)

20. During the past 4 weeks, to what extent has your physical health or emotional problems interfered with your normal social activities with family, friends, neighbours, or groups?
32. During the past 4 weeks, how much of the time has your physical health or emotional problems interfered with your social activities (like visiting with friends, relatives, etc?

(c) Sub-domain 'Fatigue/vitality' (4 items)

These questions are about how you feel and how things have been with you during For each question, please give the one answer that comes closest to the way you have been feeling. How much of the time during the past 4 weeks... (1) All of the time; (2) Most of the time; (3) A good bit of the time; (4) Some of the time; (5) A little of the time; (6) None of the time

23. Did you feel full of life?
27. Did you have a lot of energy?
29. Did you feel worn out?
31. Did you feel tired?

(d) Sub-domain 'Emotional well-being' (5 items)

These questions are about how you feel and how things have been with you during For each question, please give the one answer that comes closest to the way you have been feeling. How much of the time during the past 4 weeks... (1) All of the time; (2) Most of the time; (3) A good bit of the time; (4) Some of the time; (5) A little of the time; (6) None of the time

24. Have you been a very nervous person?
25. Have you felt so down in the dumps that nothing could cheer you up?
26. Have you felt calm and peaceful?
28. Have you felt downhearted and blue?
30. Have you been a happy person?

Note that the answers to the 36 questions were recoded on a scale from 0 to 100 with 100 indicating the highest levels of health (ie negatively-phrased questions being coded in reverse polarity).

These were then divided into the eight sub-domains (four physical health, four emotional well-being, with the recoded variables being summed then divided by the number of questions asked:

Cronbach's Alpha for the four Physical Well-Being measures (sub-domains) is 0.838.
For the four Emotional Well-Being measures (sub-domains), Alpha=0.684.

For peer review only

CASP-12 (v2) Item	Wording
1-3 Control	1 My age prevents me from doing the things I would like to.<br>(1) Often; (2) Sometimes; (3) Not often;<br>(4) Never<br>2 I feel what happens to me is out of my control.<br>Answer (1)-(4)<br>3 I feel left out of things<br>Answer (1)-(4)
4-6 Autonomy	4 I can do the things I want to do.<br>Answer (1)-(4)<br>5 Family responsibil prevent me from doing what want to do.<br>Answer (1)-(4)<br>6 I feel that I can please myself what I do.<br>Answer (1)-(4)<br>6? Shortage of money stops CM doing things wants to do.<br>Answer (1)-(4)
7-9 Self-Realisation	7 I look forward to each day.<br>Answer (1)-(4)<br>8 I feel that my life has meaning.<br>Answer (1)-(4)<br>9 I enjoy the things that I do.<br>Answer (1)-(4)<br>9? On balance I look back on life with sense of happiness.<br>Answer (1)-(4)
10-12 Pleasure	10 I feel full of energy these days.<br>Answer (1)-(4)<br>11 I feel that life is full of opportunities.<br>Answer (1)-(4)<br>12 I feel that the future looks good for me.<br>Answer (1)-(4)

Cronbach's Alpha for the twelve CASP items is 0.861.

For peer review only

Appendix 4 Rutter Behaviour scale, NCDS aged 16 (18-point version asked to mother)

Below are a series of descriptions of behaviour often shown by young people. Please ask the mother about each one and ring the appropriate number to show the degree to which this description is true of the study child:

0 Does not apply
1 Applies somewhat
2 Certainly applies

1	Restless, difficulty staying seated
2	Squirmy,fidgety child
3	Often destroys others property
4	Frequently fights, quarrelsome
5	Not much liked by other children
6	Often worries about things
7	Does things on own, rather solitary
8	Irritable, flies off the handle
9	Appears miserable,unhappy & tearful
10	Twitches,mannerisms,tics-face or body
11	Frequently sucks thumb or fingers
12	Frequently bites nails or fingers
13	Is often disobedient
14	Cannot settle more than a few moments
15	Fearful of new situations or things
16	Fussy, over particular
17	Often tells lies
18	Bullies other children

Max score=36

Cronbach's Alpha for age 16 Rutter Scale = 0.687.
Age 11 Rutter scale has 14 items; Alpha=0.682.
Age 7 Rutter scale has 14 items; Alpha=0.669.

For peer review only

Appendix 5:

Measurement models for the eight latent variables in the SEM analysis, together with reported goodness of fit criteria (CFI, TLI and RMSEA)

(a) FAMDIFFS: Family material difficulties at Age 7 years (1.00, 1.00, 0.00)

	Indicator loading (β)	Standard Error
Housing	0.46	0.02
Finance	0.79	0.02
Unemployment	0.69	0.02

(b) Age7cog: Cognition at Age 7 years (0.96, 0.88, 0.105)

Arithmetic	0.67	0.01
Copying designs	0.44	0.01
Draw-A-Man	0.48	0.01
Southgate reading	0.69	0.01

(c) Age11Cog: Cognition at age 11 years (1.0, 0.99, 0.018)

NFER General ability	0.78	0.01
Reading comprehension	0.65	0.01
Maths	0.79	0.01
Copying designs	0.12	0.01

(d) Age16cog: Cognition at Age 16 years (0.97, 0.91, 0.172)

Reading comprehension	0.82	0.01
Mathematics	0.83	0.00
Teacher assessment Eng Ability	0.85	0.00
Teacher assessment Math Abil	0.86	0.01

(e) Age50Cog: Cognition at age 50 years (0.98, 0.94, 0.068)

Letter Cancellation Speed	0.12	0.01
Word Recall	0.80	0.01
Delayed word recall	0.80	0.01
Animal naming	0.85	0.01

(f) PWB: Physical Well-Being at age 50 years (0.99, 0.97, 0.07)

Physical functioning	0.73	0.01
Role limitations due to physical health	0.77	0.01
Pain	0.82	0.01
General health	0.76	0.01

(g) EWB: Emotional Well-Being at age 50 years (0.99, 0.95, 0.077)

Role limitations due to emotional problems	0.62	0.01
Fatigue/vitality	0.86	0.01
Emotional well-being	0.84	0.01
Social functioning	0.12	0.02

For peer review only

(h) CASP12: Quality of Life at age 50 years (0.88, 0.85, 0.088)

	Indicator loading (β)	Standard Error
Control1	0.39	0.01
Control2	0.55	0.01
Control3	0.55	0.01
Autonmy1	0.49	0.01
Autonmy2	0.38	0.01
Autonmy3	0.37	0.01
Pleasur1	0.75	0.01
Pleasur2	0.69	0.01
Pleasur3	0.72	0.01
SlfReal1	0.74	0.01
SlfReal2	0.72	0.01
SlfReal3	0.80	0.01

For peer review only

Appendix 6: Calculation of Indirect Effect and Total Effects in the Structural Equation Model

A Indirect effects from early-life circumstances to Age 50 outcomes (where total effect size >.01 and statistically significant)

Effects from	To	Total effect	Total indirect	Significant direct effect? (p<.001)	Total effect significant?
SC Birth	Age 50 Cog	0.117	0.117	No	Yes
Birthwt	Age 50 Cog	0.047	0.030	No	Yes
Rutter7	Age 50 Cog	-0.065	-0.071	No	Yes
Rutter11	Age 50 Cog	-0.038	-0.025	No	Yes
Rutter16	Age 50 Cog	-0.018	-0.023	No	Yes
Masmoke	Age 50 Cog	-0.027	-0.027	No	Yes
Breastf	Age 50 Cog	0.039	0.039	No	Yes
SC Birth	PWB	0.081	0.048	No	Yes
Birthwt	PWB	0.011	0.011	No	Yes
Breastf	PWB	0.014	0.014	No	Yes
Rutter7	PWB	-0.043	-0.059	No	Yes
Rutter11	PWB	-0.081	-0.039	No	Yes
Rutter16	PWB	-0.074	-0.002	Yes	Yes
Masmoke	PWB	-0.010	-0.010	No	Yes
Rutter7	EWB	-0.046	-0.046	No	Yes
Rutter11	EWB	-0.080	-0.061	No	Yes
Rutter16	EWB	-0.133	-0.003	Yes	Yes
SC Birth	QoL	0.055	0.033	No	Yes
Rutter7	QoL	-0.048	-0.034	No	Yes
Rutter11	QoL	-0.049	-0.049	No	Yes
Rutter16	QoL	-0.103	-0.004	Yes	Yes

B Indirect effects from early-life circumstances to early mid-life outcomes (total effect size >.01 and statistically significant)

Effects from	To	Total effect	Total indirect	Significant direct effect? (p<.001)	Total effect significant?
SC Birth	SC42yrs	0.167	0.113	Yes	Yes
SC Birth	Quals	0.184	0.135	Yes	Yes
Birthwt	SC42yrs	0.030	0.030	No	Yes
Birthwt	Quals	0.035	0.035	No	Yes
Masmoke	SC42yrs	-0.027	-0.027	No	Yes
Masmoke	Quals	-0.032	-0.032	No	Yes
Breastf	SC42yrs	0.039	0.039	No	Yes
Breastf	Quals	0.047	0.047	No	Yes

For peer review only

C Indirect effects from early life circumstances to Age 50 outcomes (total effect size <.01 or not statistically significant)

Effects from	to	Total effect	Total indirect	Significant direct effect? (p<.001)	Total effect significant?
SC Birth	EWB	0.031	0.012	No	No
Birthwt	EWB	0.007	0.004	No	No
Masmoke	EWB	-0.003	-0.003	No	Yes
Breastf	EWB	0.005	0.005	No	Yes
Birthwt	QoL	0.003	0.006	No	No
Masmoke	QoL	-0.005	-0.005	No	Yes
Breastf	QoL	0.008	0.008	No	Yes

Commentary: Three direct influences stand out in Table A, each involving the Rutter scores at age 16 years (see Appx 4).

These are modest direct influences on PWB and QoL, but a relatively stronger negative influence on EWB at age 50 years.

Two direct influences stand out in Table B: from social class at birth to social class at 42 years and Qualifications obtained. The direct influences are shown in our estimated pathways diagram (Fig.2), but we also see in Table C there is an additional indirect effect (via childhood cognition at ages 7/11/16), over twice as large as the direct effect in both cases.

For peer review only

Appendix 7: Separate SEM path diagrams for men and women

Running the analyses separately by sex, we see that certain pathways are significant for one sex but not the other (p<.001) and certain path coefficients are notably different:

Men only (N=3815)

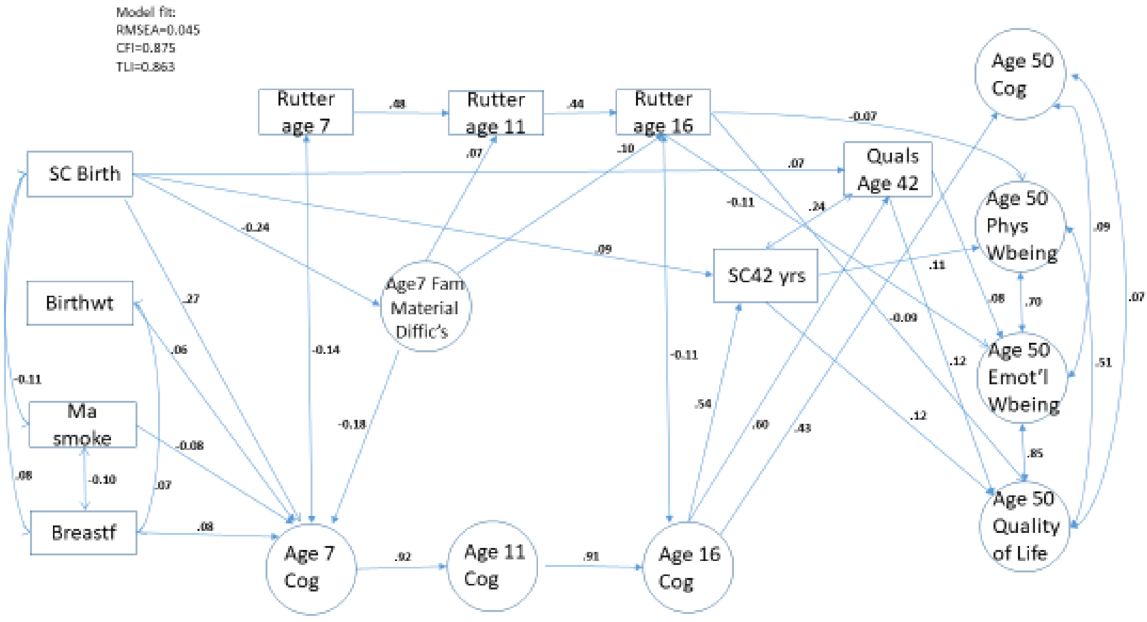

Women only (N=4209)

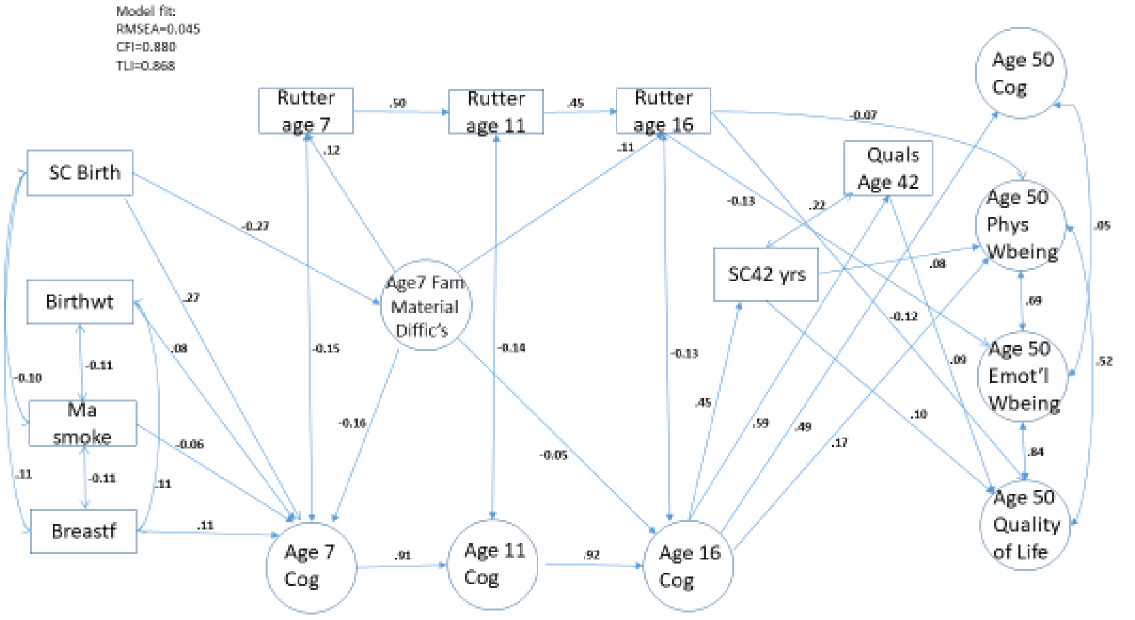

Appendix 8: Multigroup analysis by sex

An MPlus multigroup run by sex was performed as a sensitivity analysis, in which each of the 50 possible paths in Fig.1 was labelled m1-m50, and (in the female model) f1-f50, then a set of 50 'difference' variable was defined diff(i)=m(i)-f(i).

Testing the significance of these 50 'diff' variables, 6 were found to be significant, and the other 43 non-significant.

The MPlus multigroup model was then re-run, constraining all the 44 non-significant paths to be equal for males and females, but allowing all the other six paths to vary, thus increasing the parsimony of the model.

The six paths found to be significantly different between men and women are shown in this table:

	Men: Estimate	P-val	Women: Estimate	P-val
Age 50 Cog on Age 16 Cog	0.43	0.000	0.49	0.000
SC 42yrs on SC Birth	0.09	0.000	0.01	0.461
Phys WBeing on Age 16 Cog	0.07	0.013	0.17	0.000
Age 50 Cog on SC 42yrs	0.06	0.002	-0.02	-0.146
Quals Age 42 on Age 16 Cog	0.60	0.000	0.59	0.000
Age 16 Cog on Age 11 Cog	0.91	0.000	0.92	0.000

Model fit:

RMSEA 0.047
CFI 0.861
TLI 0.855

The first three paths are notable. The fourth is only significant at the .002 level, and in the case of the last two, the difference in the point estimates, though significant, is so small as to be unremarkable.

We see that women's Age 50 cognitive ability has a stronger link with Age 16 cognition (β=.49) than men's (β=.43), and that for men there is a direct link from SC Birth to SC 42 yrs (β=.09), a result not significant for women.

Finally, the effect from Age 16 Cog to Phys WBeing is significant for women (β=.17), a result not significant for men.

For peer review only

BMJ Open

Exploring the role of early life circumstances, abilities and achievements on well-being at age 50 years: evidence from the 1958 British Birth Cohort Study

Journal:	*BMJ Open*
Manuscript ID	bmjopen-2019-031416.R3
Article Type:	Original research
Date Submitted by the Author:	18-Jan-2020
Complete List of Authors:	Dodgeon, Brian; UCL Institute of Education Centre for Longitudinal Studies, Social Science<br>Patalay, Praveetha; University College London, Centre for Longitudinal Studies<br>Ploubidis, George; University College London Institute of Education, Centre for Longitudinal Studies<br>Wiggins, Richard; University College London, Centre for Longitudinal Studies
**Primary Subject Heading**:	Public health
Secondary Subject Heading:	Mental health, Research methods, Sociology, Epidemiology
Keywords:	MENTAL HEALTH, PUBLIC HEALTH, STATISTICS & RESEARCH METHODS, mid-life physical and emotional well-being

SCHOLARONE™
Manuscripts

I, the Submitting Author has the right to grant and does grant on behalf of all authors of the Work (as defined in the below author licence), an exclusive licence and/or a non-exclusive licence for contributions from authors who are: i) UK Crown employees; ii) where BMJ has agreed a CC-BY licence shall apply, and/or iii) in accordance with the terms applicable for US Federal Government officers or employees acting as part of their official duties; on a worldwide, perpetual, irrevocable, royalty-free basis to BMJ Publishing Group Ltd ("BMJ") its licensees and where the relevant Journal is co-owned by BMJ to the co-owners of the Journal, to publish the Work in this journal and any other BMJ products and to exploit all rights, as set out in our licence.

The Submitting Author accepts and understands that any supply made under these terms is made by BMJ to the Submitting Author unless you are acting as an employee on behalf of your employer or a postgraduate student of an affiliated institution which is paying any applicable article publishing charge ("APC") for Open Access articles. Where the Submitting Author wishes to make the Work available on an Open Access basis (and intends to pay the relevant APC), the terms of reuse of such Open Access shall be governed by a Creative Commons licence – details of these licences and which Creative Commons licence will apply to this Work are set out in our licence referred to above.

Other than as permitted in any relevant BMJ Author's Self Archiving Policies, I confirm this Work has not been accepted for publication elsewhere, is not being considered for publication elsewhere and does not duplicate material already published. I confirm all authors consent to publication of this Work and authorise the granting of this licence.

For peer review only

Exploring the role of early life circumstances, abilities and achievements on well-being at age 50 years: evidence from the 1958 British Birth Cohort Study

Corresponding author: Brian Dodgeon, Centre for Longitudinal Studies, UCL Institute of Education, 20 Bedford Way, London WC1H 0AL. b.dodgeon@ucl.ac.uk 020-7612-6877.
ORCID: 0000-0002-7130-8761

Co-authors:
Praveetha Patalay, Centre for Longitudinal Studies, UCL Institute of Education, 20 Bedford Way, London WC1H 0AL United Kingdom.
ORCID: 0000-0002-5341-3461

George B. Ploubidis, Centre for Longitudinal Studies, UCL Institute of Education, 20 Bedford Way, London WC1H 0AL United Kingdom.
ORCID: 0000-0002-8198-5790

Richard D. Wiggins, Centre for Longitudinal Studies, UCL Institute of Education, 20 Bedford Way, London WC1H 0AL United Kingdom.
ORCID: 0000-0007-2648-597X

For peer review only

Contributorship Statement

Brian Dodgeon performed the data management and structural equation modelling using the Mplus software, and took responsibility for completing all aspects of the paper.

Praveetha Patalay contributed to the conceptual planning of the analysis jointly with Richard Wiggins, performed preliminary regression modelling and advised on aspects of the structural equation modelling (SEM) strategy.

George Ploubidis advised on many aspects of the statistical analysis, and provided detailed comments on the drafting of the paper.

Richard Wiggins undertook the conceptual planning of the analysis jointly with Praveetha Patalay, and initiated the SEM approach, continuing to provide advice on the models as they became more complex, and collaborating with Brian Dodgeon on the write-up of the findings.

Word count:
3,157

ABSTRACT

1 Objectives

We aim to examine the relative contributions of pathways from middle childhood/adolescence to mid-life well-being, health and cognition, in the context of family socio-economic status (SES) at birth, educational achievement and early-adulthood SES. Our approach is largely exploratory, suspecting that the strongest mediators between childhood circumstances and mid-life physical & emotional well-being may be cognitive performance during school years, material and behavioural difficulties, and educational achievement. We also explore whether the effects of childhood circumstances on mid-life physical & emotional well-being differ between men and women.

2 Setting/Participants

Data were the National Child Development Study, a fully-representative British Birth Cohort sample of 17,415 people born in one week in 1958.

3 Primary/secondary outcome measures

Our four primary mid-life outcome measures are: cognitive performance; physical and emotional well-being and quality of life. Our intermediate adult outcomes are early-adulthood social class and educational/vocational qualifications.

4 Results

Using structural equation modelling, we explore numerous pathways through childhood and early adulthood which are significantly linked to our outcomes. We specifically examine the mediating effects of the following: cognitive ability at ages 7, 11 and 16 years; childhood psychological issues ; family material difficulties at age 7: housing, unemployment, finance; educational/vocational qualifications and social class position at age 42 years.

We find that social class at birth has a strong indirect effect on the age 50 outcomes via its influence on cognitive performance in childhood and adolescence, educational attainment and mid-life social class position, together with small direct effects on qualifications and social class position at age 42 years. Teenage cognitive performance has a strong positive effect on later physical health for women, whilst educational/vocational qualifications have a stronger positive effect on emotional well-being for men.

4 Conclusion

Our findings provide an understanding of the legacy of early life on multiple aspects of mid-life health, well-being, cognition and quality of life, showing stronger mediated links for men from childhood social class position to early adult social class position. The observed effect of qualifications supports those arguing that education is positively associated with subsequent cognitive functioning.

Article Summary

Strengths and limitations of this study:

- The richness of its longitudinal dimension, conveying the importance of early life circumstances upon early adult and mid-life measures of achievement, health and well-being;
- The integrated nature of the analysis simultaneously models pathways to four mid-life outcomes, three of which are strongly correlated after the inclusion of several key early-life antecedents;
- It makes optimal use of all the available longitudinal data by employing Full Information Maximum Likelihood estimation to take account of item non-response;
- It shows the enduring importance of parental social class for cognitive performance and later life outcomes, controlling for family material difficulties in childhood.

For peer review only

- A potential limitation is that our model does not include a broader set of demographic and psychosocial factors: a result of our decision to restrict the number of early-life influences to avoid over-complexity in the statistical modelling. There is also a small amount of attrition bias between birth and age 50.

INTRODUCTION

Authors since the 1990s have advanced the importance of a life course approach to explore the relationship between early life circumstances and later life health outcomes [1, 2]. Carr [3] emphasises how diverse early life experiences affect physical, emotional and cognitive health in later life by drawing upon a wealth of longitudinal data across the United States, Australia and Europe. Our empirical study is based on a single British Birth Cohort which covers several waves of data collection from birth (1958), middle childhood (age 7 & 11), adolescence (age 16) and mid-life at ages 42 and 50 years for over 8,000 sample members. Age 50 years represents a specific mid-point of mid-life (40-60) as defined by Midlife Development in the United States (MIDUS): Brim et al [4] suggest "Midlife has been described as the last uncharted territory of the life course". To this extent our conceptual framework is largely informed by Elder [5]: it may be described as a cumulative (dis-) advantage model [6] where we examine the legacy of early life circumstances, notably the British Registrar General's classification of occupations (RG social class) [7] as having lasting effects on health and well-being at age 50 years. Our approach implies a reciprocal relationship between socio-economic status (SES) and health, and allows for the possibility that favourable circumstances in later life can act counter to the effects of earlier disadvantage [8].

To this end we select outcomes at age 50 years covering four distinct areas: self-assessed quality of life, physical and emotional well-being, and a cognitive ability test.

Our research interest is in exploring the extent to which these outcomes inter-relate, adopting an empirical view that, taken together, these measures would tell us more about the consequences of the past than studied individually [9]. There is a growing body of evidence based on United Kingdom (UK) longitudinal studies [10,11,12] that provides a strong argument for looking at these outcomes together within the same model. Cooper et al [13] provided both substantive and empirical evidence for examining gender differences in this analysis.

Drawn from the same rich longitudinal resource, our predictors and mediators begin with birth circumstances: birthweight, breastfeeding, maternal smoking during pregnancy and parental SES. From the age 7 sweep of the study we include the effects of family material difficulties such as unemployment, finance and housing, in conjunction with childhood scores of socio-emotional adjustment and cognitive performance, each measured on three occasions spanning the ages 7, 11 and 16 years. The inclusion of highest qualification and SES at age 42 in the model enables us to trace which childhood effects persist, and which are built upon or attenuated by education and social mobility.

Our analysis builds upon the framework adopted by Wood et al [14] on the prediction of mental well-being as a single outcome [15] across four UK birth cohort studies, which showed that childhood SES is directly and indirectly (through adult socioeconomic pathways) linked to adult

For peer review only

emotional well-being. We expand upon their approach by including four outcome measures, albeit for a single birth cohort.

Our analysis adopts a structural equation modelling (SEM) approach [16], in order to explore the pathways from childhood and adolescence to our multivariate mid-life outcomes for both men and women.

DATA AND METHODS

Our data source, the British National Child Development Study (NCDS) is fully representative of the population of Great Britain (GB), having interviewed the parents of 98.5% of all babies born in the same week of 1958 [17], and followed them up longitudinally with ten subsequent interviews throughout life. It provides operational measures regarding birth circumstances, the early and teen years covering ages 7, 11 and 16, and adulthood at ages 42 and 50 respectively.

The analysis is based upon 3815 male and 4209 female cohort members present at each occasion who all have at least one genuine response for each of the four separate age 50 life domains, and who also participated in at least one of the three cognitive and socio-emotional assessments, in order to permit a reliable strategy for handling missing items.

We are aware there is a certain amount of attrition bias as a result of differential loss-to follow-up from the 17,415 cohort members present at the birth study in 1958. As a check on the impact of sample loss, we were reassured by the close match of the distribution of the birth social class variable within our sample of 8,024 against that of the same variable within the full 17,415 birth sample (chi-square=1.29, 5d.f., non-significant) (see Appendix 1).

Our outcomes at age 50 years cover four distinct measures, the first two being self-reported physical and emotional well-being (PWB and EWB respectively) measured using sub-scales [18] of the RAND 36-Item Short-Form Health Survey [19-23] (hereafter SF-36). Third, a self-reported measure of quality of life (QoL) covering the life domains of 'Control, Autonomy, Self-Realisation and Pleasure (CASP-12 v2) [24]: a theoretically-informed measure of subjective well-being, closely following the approach adopted by Ed Diener and his colleagues [25]. The fourth domain outcome, cognitive ability (hereafter Age50Cog) consists of four cognitive tests examining memory (word recall & delayed recall), executive function (animal naming), attention and mental speed (letter cancellation) [26].

Appendix 2 lists all of the manifest and latent variables employed in the analysis. Indicators for cognitive function at age 50 years are listed here, while those for physical, emotional and quality of life outcomes are expanded upon more fully in Appendix 3. Childhood behavioural indicators are listed in Appendix 4 following Rutter [27]. We recognise of course that Rutter scores may be sub-divided into constituent dimensions of problem behaviour (withdrawn/ anxious / oppositional/ inattentive [28], but for this paper we prefer to use Rutter total scores, following Parsons et al [29].

In fitting our structural equation models using the MPlus software [30] we obtain direct and indirect (mediated) effects of our background variables in terms of childhood social origins and social adjustment, on outcomes in early adulthood, and mid-life at age 50 years. We regard all four of our

For peer review only

birth indicators as primary predictors, valuing the opportunity to assess the relative impact of each for policy interventions [31]. Our birth social class variable is based on father's occupation, since in 1958 when the cohort were born, only 38% of mothers were economically active.

The sets of predictors determining our paths differ slightly for each of the four outcomes so as to avoid the problem of 'seemingly unrelated regression' [32]

We employ full information maximum likelihood estimation (FIML) [33]. This addresses the issue of item non-response arising where questions are avoided or not answered by certain participants.

The SEM procedure that follows begins with an analysis of the sample as a whole, followed by separate analyses for men and women, supported by a multigroup analysis [34] to test for the structural equivalence of the models for men and women. Model 'goodness of fit' is assessed via a combination of conventional criteria: 'root mean square error' (RMSEA), 'comparative fit index', (CFI) and 'Tucker Lewis fit index', (TLI) where RMSEA<0.05, CFI>0.90 and TFI> 0.90 would typically provide evidence of 'good fit,' though each of these criteria individually are regarded as indicative, and are not always strictly adhered to as arbiters of model validity [35].

Having a large number of SEM pathways involving 261 free parameters, we had regard to the n:q rule [36] and were satisfied that our sample-size-to-parameters ratio was comfortably within Jackson's guideline of 10:1. Our application of SEM involved the use of modification indices, which led to our running correlations on suggested variables. All our final models were identified, all manifest variables loading onto the respective latent variables with satisfactory fit. The details of the individual assessments of our measurement models appear in Appendix 5.

Patient and Public involvement statement

Participant consent was obtained from parents of cohort members at the initiation of the original birth cohort study in 1958. On attaining adulthood, permission was obtained from cohort members themselves at each subsequent longitudinal sweep.

RESULTS

The results begin with consideration of the inter-correlations between our mid-life outcome measures. Further univariate descriptives are contained in Appendix 2. Figure 1 contains a final SEM diagram for the sample as a whole. Subsequent reference will be made to Appendix 6 for the details of mediation, total and indirect effects. Appendix 7 contains separate analyses by sex, and Appendix 8 contains further information on our multigroup analysis based on gender.

Note that all coefficients reported in the Results section are standardised.

(a) Descriptives & correlations

As a preliminary inspection of the inter-relationships between our four outcome measures, Table 1 contains a correlation matrix of the four summative indices based on these measures for both men and women.

For peer review only

Table 1. Associations between the four sets of mid-life outcomes as summative indices (Men/Women)

Age 50 Domain	Age 50 Cog	PWB	EWB	QoL
Cognitive Ability (Age 50 Cog)	1.00/1.00			
Physical Well-Being (PWB)	0.12/0.15	1.00/1.00		
Emotional Well-Being (EWB)	0.12/0.09	0.57/0.58	1.00/1.00	
Quality of Life (QoL)	0.15/0.10	0.48/0.48	0.63/0.64	1.00/1.00

For peer review only

Interestingly, QoL, PWB and EWB demonstrate moderate associations of inter-correlation. While cognitive ability is positively related to the physical and emotional well-being measures, the strength of the association is modest, with PWB having a higher coefficient for women and EWB & QoL a higher coefficient for men.

Fig. 1 contains estimates of association amongst these outcomes as defined by measurement models under a full SEM analysis. As a check on the robustness of our statistical findings the SEM analyses were subject to bootstrap analysis under 1,000 repetitions. We also carried out a check on the influence of outliers (results available on request).

(b) Birth social class effects on early mid-life outcomes

The path diagram shown in Fig 1 provides the model results for the sample as a whole showing only statistically significant paths at a p-level of .001, employing a Bonferroni correction for the fifty path coefficients being estimated (0.05/50=0.001) [37].

Fig 1. Estimated pathways of the Structural Equation Model, N=8024 (showing paths significant at p < .001)

[insert Fig.1 here]

Whilst there are various pathways that connect early life circumstances with cognitive performance, family material difficulties and social adjustment, there remains a small direct effect of social class at birth on each of our two early adult outcomes: Age 42 Social class and educational/vocational qualifications (0.05 for both paths). These two 'interim' or 'pivotal' destinations, in turn, have notable effects on our four mid-life outcomes at age 50, which we describe under (d) below.

(c) Indirect effects via pathways through childhood mediators

Inspecting the paths in Fig.1 from birth circumstances through ages 7, 11 and 16, we see firstly, that all four of our birth characteristics have direct effects on cognitive performance at age 7 years, when children are in primary school. Social class at birth has the most notable association with age 7 cognition (.27). This cognitive variable in turn has a strong direct effect on age 11 cognition (.92) and similarly for age 11 on cognitive performance at age 16 (.92).

There is a clear path via cognitive ability throughout childhood and adolescence, to adult achievement as indicated by Age 42 social class and educational qualifications (Age 16 Cog to Social class 42yrs 0.50, Age 16 Cog to Quals 0.59).

This legacy of childhood cognitive performance is unsurprising, but interestingly Age 16 cognition also has a direct association with age 50 physical well-being (0.12) besides more naturally, Age 50 cognition (0.47).

Family material difficulties in childhood, themselves associated with birth social class (-0.26), also play their part in understanding a child's trajectory in cognitive performance. Characterised by issues with housing, finance and employment when a child is 7 years old, these family material difficulties are seen to have negative consequences for cognitive performance at age 7 years (-.15) and a lagged negative effect on later performance age 16 years (-0.04).

Additional analyses also show that family material difficulties impact on a child's emotional and behavioural scores (based on Rutter) at ages 7, 11 and 16 (.09, .06 and .10 respectively). In turn these scores had small significant negative correlations with cognitive performance at each corresponding age (-.15, -.11 and -.11 respectively). But most notably, Rutter scores at these three ages are significantly correlated (.49 for age 7 to 11 and .44 for age 11 to 16), and there are direct effects from age 16 Rutter scores to three of our four age 50 outcomes: EWB (-0.13), QoL (-0.10) and PWB (-0.07).

(d) Indirect effects via adulthood mediators

Adult social class and lifetime qualifications are, together, influential predictors of each of our four mid-life outcomes: adult social class has significant predictive effects (.08 and .11) on PWB and QoL.

In general, the socially-advantaged enjoy better health and report higher levels of quality of life than their less advantaged counterparts. Interestingly, qualifications predict age 50 cognitive ability (.05), EWB (.06) and QoL (.10) but not PWB.

(e) Gender differences

Our multigroup analysis reveals that certain paths have significantly different effects for men and women. In Appendix 7 we display two separate path diagrams for men and women. For men, there is a direct effect of social class at birth on Age 42 social class and qualifications (.09 and .07 respectively), but for women there is no significant effect.

Both sexes see the birth social class effect remain equally strong for cognitive performance at age 7 years and beyond, but women experience a slightly worse negative effect of social disadvantage at birth on the latent variable 'family material difficulties' (-0.27 compared to -0.24 for men). In turn, family material difficulties have slightly more influence on Age 7 cognition (-.18) for men than for women (-.16), and a small lagged effect on Age 16 cognition only evident for women (-.05). The Rutter social adjustment scores at age 11 do not significantly correlate with the cognitive performance scores for men, but they do for women at ages 7, 11 and 16 (-.15, -.11 and -.13 compared with men's -.14 at age 7, -.11 at age 16).

For peer review only

Early adult social class has a slightly stronger influence for men than women on our age 50 outcomes PWB and QoL, and Qualifications have a significant effect on EWB for men, but not for women. In contrast, women's age 16 cognition has a significant effect on their later PWB (.17), an effect not significant for men; and the path from age 16 cognition to age 50 Cog has a stronger coefficient for women (.49) than men (.43).

We pursue the evidence for gender differences via a sensitivity analysis under MPlus which formally tests for statistical difference between paths for each sex. Appendix 8 identifies six paths where the effects for men and women are significantly different, but only three have notable differences in terms of their effect sizes in the multigroup analysis. These are:

- stronger effect for women from age 16 cognition to age 50 cognition as noted above (.49 to .43);
- direct effect of social class at birth on Age 42 social class (.09 for men, no significant effect for women); and
- link for women from Age16 Cognition to Phys WBeing (.17)(no significant effect for men)

DISCUSSION

Structural equation modelling has elucidated the nuances of the influence of pathways from childhood and early adulthood to our four related outcomes of health, well-being and cognition in mid-life. The work demonstrates the benefits of an integrated approach to defining the aspects of the life course that affect well-being in mid-life. Physical and emotional health are important factors affecting a person's subjective account of their quality of life, and PWB correlates at .69 with EWB, which in turn correlates .83 with QoL. While cognitive performance at age 50 is relatively weakly correlated, the other three are closely intertwined. These associations are similar in magnitude to those reported in table 1; however they are now estimated by taking measurement error and the legacy of the life course into account.

We also show that some of the paths to health and well-being in mid-life are different for men and women [38]. However, important communalities remain.

Social origins, in particular social class at birth, have a major indirect influence on mid-life outcomes, the mediation process beginning with a child's cognitive performance throughout formal schooling and subsequently via cognitive performance at age 16 years. Collectively, these influences are weakened or reinforced by the existence of family material difficulties in early childhood (themselves influenced by childhood social class). Whilst cognitive performance is a strong predictor of early adult social class and educational status for both men and women, social class at birth also has a lasting association with these mid-life indicators for men but not for women.

This could well suggest that women have a looser connection with the legacy of social class at birth, perhaps partly because their path to advantaged SES at age 42 may be impeded by caring responsibilities and gender inequalities in the labour market [39].

Early adult social class influences all four of our mid-life outcomes, but we see the direct effect of social class at birth on social class and qualifications in early adulthood is pronounced for men (.09 and .07 respectively), but not significant for women.

For peer review only

A relatively small but consistent direct effect of qualifications on Age 50 Cognition supports those arguing that education is positively associated with subsequent cognitive functioning [40], although we are aware that there are counter-arguments to this view [41]). Equally, the relationship between QoL and early adult social class finds support in the work of Blane et al [42].

The strength in the evidence presented here lies in the richness of its longitudinal dimension which conveys the importance of early life circumstances upon early adult and mid-life measures of achievement, health and well-being.

One limitation of our study is that our model does not fully take into account all of the potential demographic and psychosocial factors other researchers have studied as predictors of satisfactory midlife development [28]. Nevertheless, it can be argued that our birth social class variable acts as a 'proxy' for certain parental beliefs and behaviours [43]. We are embarking on developing a conceptual framework and modelling strategy to extend our empirical analyses in the future as our cohort members age: in particular, our subjects are not yet old enough for us to assess whether any decline in cognitive performance at age 50 years will lead to subsequent decline and become more influential on emotional well-being and quality of life. However, we were able to show that cognitive performance in the teen years holds a legacy for cognitive performance in mid-life. A final limitation is a certain amount of attrition bias between birth and age 50 (differential non-response) which was addressed earlier in the paper and in Appendix 1.

Social class at birth has a strong influence on early cognitive performance and beyond, which in turn has consequences for adult achievement and social class. Clearly, the presence of family material difficulties and behavioural issues will impede (mediate) this trajectory. Interestingly, childhood behaviour, in particular at age 16, has a lasting effect on mid-life PWB, EWB and Quality of Life.

In sum, health, emotion, quality of life and to a lesser extent cognitive function do not stand alone as markers of well-being in mid-life. They represent the constituents of an inter-related whole which is shaped by early life circumstances, family material difficulties, social adjustment and cognitive performance in childhood.

Acknowledgements

We would like to acknowledge the thoughtful and constructive comments of our three reviewers, which strengthened our paper.

References

1 Williams, D.R. (1990). Socioeconomic differentials in health: A review and redirection. Social Psychology Quarterly 53, 81–99.

2 Wadsworth, M. E. J. (1997). Health inequalities in the life course perspective. Social Science & Medicine 44, 859–869.

3 Carr, D (2019). Early-life influences on later life well-being: innovations and explorations. The Journals of Gerontology, Series B: Psychological Sciences and Social Sciences, 74(5), 829-831.

4 Brim, O, Ryff, G, Kessler, C.D & Ronald, C (2004). How healthy are we? A National Study of Well-Being at Midlife. Chicago, IL: University of Chicago Press.

For peer review only - http://bmjopen.bmj.com/site/about/guidelines.xhtml

5 Elder, G (1994). Time, human agency, and social change: perspectives on the life course. Social Psychology Quarterly, 57, 4-15.

6 Dannefer, D (2003) Cumulative advantage/disadvantage and the life course: Cross-fertilizing age and social science theory. The Journals of Gerontology, Series B, 58(6), S327-S337.

7 Szreter, S.R.S (1984). The Genesis of the Registrar-General's Social Classification of Occupations. British Journal of Sociology 35(4), 522-54

8 Graham, H. (2002). Building an inter-disciplinary science of health inequalities: The example of lifecourse research. Social Science & Medicine 55(11), 2005–2016.

9 Marmot, M. G, Fuhrer, R, Ettner, S.L, Marks, N.F, Bumpass, L.L and Ryff, C.D (1998) Contribution of psychosocial factors to socioeconomic differences in health. Millbank Quarterly 76, 403-48.

10 Gale, C. R, Cooper, R, Craig, L, Elliott, J, Kuh, D, Richards, M & Deary, I. J (2012). Cognitive function in childhood and lifetime cognitive change in relation to mental wellbeing in four cohorts of older people. *PLoS ONE*, *7*(9), e44860. doi: 10.1371/journal.pone.0044860

11 Clouston, S. A, Brewster, P, Kuh, D, Richards, M, Cooper, R, Hardy, R, Rubin, M.S and Hofer, S M (2013). The Dynamic Relationship Between Physical Function and Cognition in Longitudinal Aging Cohorts. *Epidemiologic Reviews* 35(1), 33–50 doi: 10.1093/epirev

12 Cooper, R, Stafford, M, Hardy, R, Aihie Sayer, A, Ben-Shlomo, Y, Cooper, C & the HALCyon Study Team (2014). Physical capability and subsequent positive mental wellbeing in older people: findings from five HALCyon cohorts. *Age 36*(1), 445-456. doi: 10.1007/s11357-013-9553-8

13 Cooper, R, Hardy, R, Aihie Sayer, A, Ben-Shlomo, Y, Birnie, K. C, Craig, L, Deary, I.J, Demakakos, P, Gallacher, J, McNeill, G, Martin, R.M, Starr, J.M, Steptoe, A, Kuh, D and the HALCyon Study Team (2011). Age and Gender Differences in Physical Capability Levels from Mid-Life Onwards: The Harmonisation and Meta-Analysis of Data from Eight UK Cohort Studies. ***PLoS One***, 6(11), e27899.

14 Wood, N, Bann, D, Hardy et al (2017) Childhood socioeconomic position and adult mental wellbeing: evidence from British birth cohort studies. PLoS One, 10, e0185798.

15 Tennant, R, Hiller, L, Fishwick, R et al (2007) The Warwick-Edinburgh Mental Well-Bing Scale (WEMWBS): development and UK validation. Health and Quality of Life Outcomes 5(1), 63.

16 Kline, R.B. (1998). *Principles and Practice of structural equation modelling*. New York. Guildford Press.

17 Power, C and Elliott, J. (2006) Cohort Profile: 1958 British birth cohort (National Child Development Study). International Journal of Epidemiology 35(1), 34-41.

18 Doll, H.A, Petersen, S.E.K and Stewart-Brown, S.L (2012) Obesity and Physical and Emotional Well-Being: Associations between Body Mass Index, Chronic Illness, and the Physical and Mental Components of the SF-36 Questionnaire. Obesity 8(2), 160-170 doi: 10.1038/oby.2000.17

For peer review only

19 Ware, J.E and Donald Sherbourne, C (1992). The MOS 36-Item Short-Form Health Survey (SF-36): I. Conceptual Framework and Item Selection. Medical Care 30(6), 473-483.

20 Brazier, J.E, Harper, R, Jones et al (1992). Validating the SF-36 health survey questionnaire: new outcome measure for primary care. British Medical Journal 305(6846): 160-164.

21 Jenkinson, C., Wright, L. and Coulter, A. (1994). Criterion validity and reliability of the SF-36 in a population sample. Quality of Life Research 3(1), 7-12.

22 Burnholt, V & Nash, P. (2011). Short Form 36 (SF-36) Health Survey Questionnaire: normative data for Wales. Journal of Public Health, 33(4), 587-683.

23 Mishra, G.D, Gale, C.R, Aihie Sayer, A, Cooper, C, Dennison, E. M, Whalley, L.J, Craig, L, Kuh, D, Deary, I. J and the HALCyon Study Team (2011) How useful are the SF-36 sub-scales in older people? Mokken scaling of data from the HALCyon programme. *Quality of Life Research*, 20 (7), 1005-1010. (doi:10.1007/s11136-010-9838-7). (PMID:21225350)

24 Wiggins, R.D, Netuveli, G., Hyde et al (2008). The evaluation of a self-enumerated scale of quality of life (CASP-19) in the context of research on ageing: a combination of exploratory and confirmatory approaches. Social Indicators Research 89(1), 61-77.

25 Diener, E and Suh, E (1997) Measuring quality of life: Economic, social, and subjective indicators Social indicators Research, 40 (1-2), 189-216.

26 Brown, M and Dodgeon, B. (2010) NCDS Cognitive Assessments at Age 50: Initial Results. CLS Working Paper 2010/1. London: Centre for Longitudinal Studies. UCL.

27 Rutter, M, Tizard, J and Whitmore, K (1970) Education, Health & Behaviour, Longman.

28 Caspi, A, Henry, B, McGee, R.O, Moffitt, T.E & Silva, P (1995). Temperamental Origins of Child and Adolescent Behavior Problems: From Age Three to Age Fifteen Child Development 66 (1), 55-68 doi: 10.1111/j.1467-8624.1995.tb00855.x

29 Parsons, S, Green, F, Ploubidis, G.B, Sullivan, A and Wiggins, R.D (2017). The influence of private primary schooling on children's learning: Evidence from three generations of children living in the UK. British Educational Research Journal 43(5), 823-847 (doi: 10.1002/berj.3300)

30 Muthen, B.O & Muthen L.K (2018) Statistical Analysis with Latent Variables: MPlus v8.1 User Manual. Los Angeles CA: Muthen & Muthen.

31 Sacker, A, Kelly, Y, Iacovou, M, Cable, N and Bartley, M. (2013) Breast feeding and intergenerational social mobility: what are the mechanisms? ***Archives of Disease in Childhood***, 98(9), 666-671.

32 Zellner, A. 1962. An efficient method of estimating seemingly unrelated regression equations and tests for aggregation bias. Journal of the American Statistical Association 57: 348–368.

33 Arbuckle, J.L. (1996). Full Information estimation in the presence of incomplete data. In Marcoulides, G.A & & Schumaker, R.E (eds) *Advanced Structural Equation Modeling: Issues and Techniques.* Mahwah, N.J.: Erlbaum Associates.

For peer review only

34 Byrne, B.M. (2012). *Structural Equation Modeling with Mplus*. Routledge Taylor & Francis Group. New York & London

35 Lai, K. & Green, S.B (2016). The problem with having two watches: Assessment of fit when RMSEA and CFI disagree. Multivariate Behavioral Research 51 (2-3), 220-239.

36 Jackson, D,L (2003) Revisiting Sample Size and Number of Parameter Estimates: Some Support for the N:q Hypothesis. Structural Equation Modeling 10(1), 128-141.

37 Weisstein, Eric W (2004) "Bonferroni Correction." From MathWorld--A Wolfram Web Resource. http://mathworld.wolfram.com/BonferroniCorrection.html

38 Arber, S. & Cooper, H. (1999). Gender differences in health in later life: the new paradox? Social Science and Medicine, 48(1), 61-76

39 Kahn, J.R, Garcia-Manglano, J and Bianchi, S.M (2014) The Motherhood Penalty at Midlife: Long-Term Effects of Children on Women's Careers. Journal of Marriage and the Family 76(1), 56-72.

40 Richards, M and Sacker, A (2010) Is education causal? Yes. International Journal of Epidemiology 40(2), 516–518.

41 Deary, I.J and Johnson, W (2010) Intelligence and education: causal perceptions drive analytic processes and therefore conclusions. International Journal of Epidemiology 39(5), 1362-1369.

42 Blane, D, Netuveli, G and Bartley, M (2007). Does quality of life at older ages vary with socio-economic position? Sociology **41**(4), 717-726.

43 Davis-Kean, P.E (2005). The Influence of Parent Education and Family Income on Child Achievement: The Indirect Role of Parental Expectations and the Home Environment. Journal of Family Psychology, 19(2), 294-304.

Footnotes

Funding

Assistance is acknowledged from ESRC grants ES/N003683/1 and ES/M008584/1.

Consent of authors

I, Brian Dodgeon, the Submitting Author, have the right to grant and do grant on behalf of all authors of the Work (as defined in the below author licence), an exclusive licence and/or a non-exclusive licence for contributions from authors who are: i) UK Crown employees; ii) where BMJ has agreed a CC-BY licence shall apply, and/or iii) in accordance with the terms applicable for US Federal Government officers or employees acting as part of their official duties; on a worldwide, perpetual, irrevocable, royalty-free basis to BMJ Publishing Group Ltd ("BMJ") its licensees and where the relevant Journal is co-owned by BMJ to the co-owners of the Journal, to publish the Work in BMJ Open and any other BMJ products and to exploit all rights, as set out in our licence.

For peer review only

The Submitting Author accepts and understands that any supply made under these terms is made by BMJ to the Submitting Author unless you are acting as an employee on behalf of your employer or a postgraduate student of an affiliated institution which is paying any applicable article publishing charge ("APC") for Open Access articles. Where the Submitting Author wishes to make the Work available on an Open Access basis (and intends to pay the relevant APC), the terms of reuse of such Open Access shall be governed by a Creative Commons licence – details of these licences and which Creative Commons licence will apply to this Work are set out in our licence referred to above.

Competing interests

All four authors declare they have no competing interests and no financial relationships with any organisation that might have an interest in the submitted work in the previous three years, and no other relationships or activities that could appear to have influenced the submitted work.

Data Sharing statement

The analysis is based on secondary data available for download from the UK Data Archive at https://beta.ukdataservice.ac.uk/datacatalogue/series/series?id=2000032. Syntax used to derive variables for this analysis can be obtained by e-mailing the corresponding author, Brian Dodgeon.

Ethics

Ethical approval was not required, since the study used the secondary data of the National Child Development Study, which itself had received ethics consent from the London Research Ethics Committee, ref 08/H0718/29 for its age 50 survey, the source of our outcome data. Multicentre Research Ethics Committee (MREC) approval was received for earlier NCDS surveys.

For peer review only

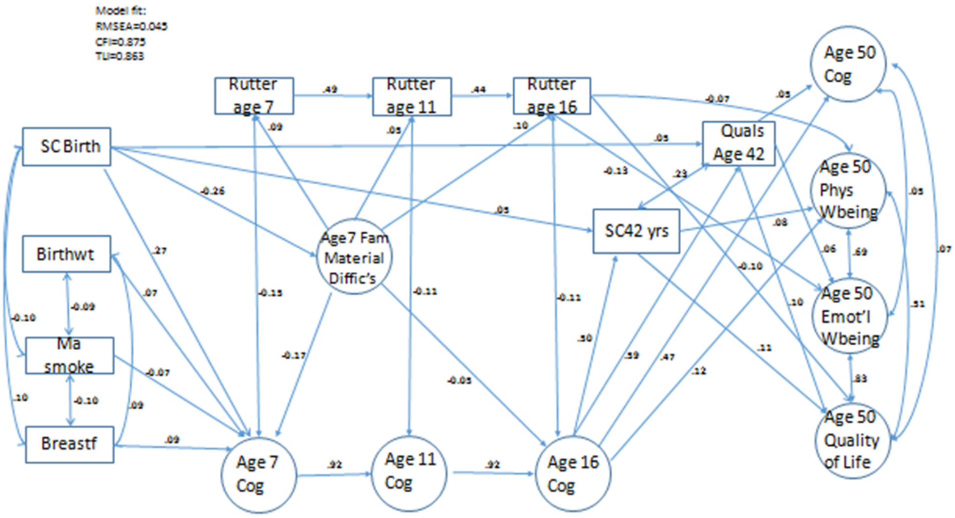

Fig 1. Estimated pathways of the Structural Equation Model, N=8024

90x50mm (300 x 300 DPI)

Appendix 1

Check for attrition bias between full NCDS sample at birth and Analysis sample

Comparison of distributions of the same birth social class variable:

Father's social class at birth	Full birth survey N=17,415	Valid %	Analysis sample N=8024	Valid %
Class I (Professional)	746	4.5	386	5.2
Class II (Intermediate)	2133	13.0	1127	15.1
Class III (Skilled Manual/Non-Manual)	9981	60.6	4498	60.1
Class IV (Semi-Skilled Manual)	1995	12.1	871	11.6
Class V (Unskilled Manual)	1616	9.8	603	8.1
Total (valid Sclass)	16471	100.0	7485	100.0
Insufficient data on parental Social Class	944		539	
Total	17415		8024	

(chi-square=1.29, 5d.f., non-significant)

For peer review only

Appendix 2: Description of manifest variables, age CM at data collection; latent variable labelling

Variable	Survey Sweep	Range of recorded codes	Mean	St'd Dev'	% missing	Latent Variable
Early years						
Social class of father (GRO 1951)	Birth	1-6 (1=low)	2.98	0.92	6.7	
Birthweight	Birth	1 <2.5 kg<br>2 >=2.5kg	1.94	0.23	2.8	
Did mother smoke after 4th month of pregnancy	Birth	1-4(No/Variable Med'm/Heavy)	1.7	1.10	3.6	
Sex	Birth	1,2	52.5% female		0	
Mother breastfed child	Age 7	1 No; 2 <1 mth; 3 >1 month	2.15	0.86	10.4	
Problem arithmetic score	Age 7	0-10	5.38	2.43	9.1	Age7cog
Copying designs test age 7	Age 7	0-12	7.19	1.93	9.1	
Draw-A-Man test score	Age 7	0-51	24.40	6.95	10.6	
Southgate reading test score	Age 7	0-30	24.43	6.36	8.9	
Fam difficulties by age 7: Housing (health visitor assess't)	Age 7	0 No; 1 D/K 2Yes	0.17	0.51	9.9	Family Material Difficult's Age 7
Fam difficulties by age 7: Finance	Age 7	0,1,2	0.21	0.53	10.0	
Fam difficulties by age 7: Unempl	Age 7	0,1,2	0.11	0.38	10.0	
NFER general ability test	Age 11	0-80	46.14	15.2	12.0	Age11cog
Reading Comprehension score	Age 11	0-40	16.97	5.95	12.0	
Maths score	Age 11	0-40	18.40	10.1	12.0	
Copying designs test age 11	Age 11	0-12	8.45	1.40	12.2	
Rutter behavioural score (age 7) (mother assess't at each age)	Age 7	0-28	6.13	3.50	9.7	
Rutter behavioural scor (age 11)	Age 11	0-28	6.13	3.41	12.8	
Rutter behavioural score (age 16	Age 16	0-36	3.91	3.42	24.4	
Adolescence						
Reading Comprehension age 16	Age 16	0-40	26.58	6.23	23.1	Age16cog
Maths test age 16	Age 16	0-40	13.83	6.96	23.4	
Teacher assess't English ability	Age 16	1-5	3.44	1.12	22.9	
Teacher assess't Maths ability	Age 16	1-5	3.11	1.19	23.5	
Early Mid-Life						
Social class at age 42	Age 42	1-6 (1=low)	3.99	1.24	13.9	
Highest qual'n (acad or vocat) achieved by age 42	Age 42	0-5 (0=no quals; 1-5=NVQ)	2.68	1.36	0	
Later Mid-Life						
Latter cancellation speed	Age 50	0-800	334.60	87.8	3.5	Age50cog
Word recall	Age 50	0-10	6.60	1.46	2.1	
Delayed word recall	Age 50	0-10	5.49	1.81	2.7	
Animal naming test	Age 50	0-65	22.53	6.22	2.1	
SF-36 phys. health (4 scores: see Appx.3. Summative stats shown)	Age 50	0-100 (each of 4 scores)	315.4	83.8	0.2	PhysWbeing Age 50
SF-36 mental health (4 scores)	Age 50	0-100 (4 scores)	276.6	63.3	0.3	Emot'l Wbeing Age 50
CASP-12 (3 control scores, 3 autonomy, 3 pleasure, 3 self-realisation – see Appx.3)	Age 50	0,1,2,3 (each of 12 scores)	23.94	5.42	0.7	Quality of Life Age 50

For peer review only

For peer review only

Appendix 3. SF-36 scale item number, response options and wording by Model Domain, and Quality of Life based on CASP-12 (v2) items

Physical Health Domain

Sub-domain Gen_hlth (2 items, self-assessed general health)

1. In general, would you say your health is: (1) Excellent; (2) Very good; (3) Good; (4) Fair; (5) Poor
2. Compared to one year ago, how would you rate your health in general now? (1) Much better now than one year ago; (2) Somewhat better now than 1 year ago; (3) About the same; (4) Somewhat worse now than 1 year ago; (5) Much worse now than one year ago

Sub-domain Lim_phys (4 items, health now limiting physical activities)

The following items are about activities you might do during a typical day. Does your health now limit you in these activities? If so, how much? (1) Yes, limited a lot; (2) Yes, limited a little; (3) No, not limited at all

3. Vigorous activities, such as running, lifting heavy objects, participating in strenuous sports
4. Moderate activities, such as moving a table, pushing a vacuum cleaner, bowling, or playing golf? 5. Lifting or carrying groceries?
5. Climbing several flights of stairs?
6. Climbing one flight of stairs?
7. Bending, kneeling, or stooping?
8. Walking more than a mile?
9. Walking several blocks?
10. Walking one block?
11. Bathing or dressing yourself?

21. How much bodily pain have you had during the past 4 weeks? (1) None; (2) Very mild; (3) Mild; (4) Moderate; (5) Severe; (6) Very severe

Emotional Well-Being Domain

These questions are about how you feel and how things have been with you during For each question, please give the one answer that comes closest to the way you have been feeling. How much of the time during the past 4 weeks... (1) All of the time; (2) Most of the time; (3) A good bit of the time; (4) Some of the time; (5) A little of the time; (6) None of the time

23. Did you feel full of pep?
24. Have you been a very nervous person?
25. Have you felt so down in the dumps that nothing could cheer you up?
26. Have you felt calm and peaceful?
27. Did you have a lot of energy?
28. Have you felt downhearted and blue?
29. Did you feel worn out?
30. Have you been a happy person?
31. Did you feel tired?

For peer review only

SF-36 Item	Wording
1 Self-assessed General Health<br>*(Domain in our model: Phys Health)*	In general, would you say your health is:<br>(1) Excellent; (2) Very good; (3) Good;<br>(4) Fair; (5) Poor
2 Self-assessed General Health compared to one year ago<br>*(Domain: Phys Health)*	2. Compared to one year ago, how would you rate your health in general now?<br>(1) Much better now than one year ago;<br>(2) Somewhat better now than 1 year ago;<br>(3) About the same;<br>(4) Somewhat worse now than 1 year ago;<br>(5) Much worse now than one year ago
Health now limiting specific physical activities:<br><br>3. Vigorous activities?<br>*(Domain: Phys Health)*<br><br>4. Moderate activities?<br>*(Domain: Phys Health)*<br><br>5. Lifting or carrying groceries?<br>*(Domain: Phys Health)*<br>6. Climbing several flights of stairs?<br>*(Domain: Phys Health)*<br>7. Climbing one flight of stairs?<br>*(Domain: Phys Health)*<br>8. Bending, kneeling, or stooping?<br>*(Domain: Phys Health)*<br><br>9. Walking more than a mile?<br>*(Domain: Phys Health)*<br><br>10. Walking several blocks?<br>*(Domain: Phys Health)*<br><br>11. Walking one block?<br>*(Domain: Phys Health)*<br><br>12. Bathing or dressing yourself?<br>*(Domain: Phys Health)*	The following items are about activities you might do during a typical day. Does your health now limit you in these activities? If so, how much?<br>3 Vigorous activities, such as running, lifting heavy objects, participating in strenuous sports<br>(1) Yes, limited a lot;<br>(2) Yes, limited a little;<br>(3) No, not limited at all<br>4. Moderate activities, such as moving a table, pushing a vacuum cleaner, bowling, or playing golf?<br>Answer (1)(2)(3)<br>5. Lifting or carrying groceries?<br>Answer (1)(2)(3)<br>6. Climbing several flights of stairs?<br>Answer (1)(2)(3)<br>7. Climbing one flight of stairs?<br>Answer (1)(2)(3)<br>8. Bending, kneeling, or stooping?<br>Answer (1)(2)(3)<br>9. Walking more than a mile?<br>Answer (1)(2)(3)<br>10. Walking several blocks?<br>Answer (1)(2)(3)<br>11. Walking one block?<br>Answer (1)(2)(3)<br>12. Bathing or dressing yourself?<br>Answer (1)(2)(3)
During the past 4 weeks, have you had any of the following problems with your work or other regular daily activities as a result of your physical health?<br>13. Cut down the amount of time you spent on work or other activities?<br>*(Domain: Phys Health)*<br>14. Accomplished less than you would like	13. Cut down the amount of time you spent on work or other activities?<br>(1) Yes (2) No<br>14. Accomplished less than you would like?

For peer review only

(Domain: Phys Health)<br>15. Were limited in the kind of work or other activities<br>*(Domain: Phys Health)*<br>16. Had difficulty performing the work or other activities<br>*(Domain: Phys Health)*	(1) Yes (2) No<br>15. Were limited in the kind of work or other activities<br>(1) Yes (2) No<br><br>16. Had difficulty performing the work or other activities (for example, it took extra effort)<br>(1) Yes (2) No
During the past 4 weeks, have you had any of the following problems with your work or other regular daily activities as a result of any emotional problems (such as feeling depressed or anxious)?<br><br>17. Cut down the amount of time you spent on work or other activities<br>*(Domain: Emotional WBeing)*<br>18. Accomplished less than you would like<br>*(Domain: Emotional WBeing)*<br>19. Didn't do work or other activities as carefully as usual<br>*(Domain: Emotional WBeing)*	 <br><br><br><br><br>17. Cut down the amount of time you spent on work or other activities?<br>(1) Yes (2) No<br>18. Accomplished less than you would like?<br>(1) Yes (2) No<br>19. Didn't do work or other activities as carefully as usual?<br>(1) Yes (2) No
20. During the past 4 weeks, to what extent has your physical health or emotional problems interfered with your normal social activities with family, friends, neighbours, or groups?<br>*(Domain: Emotional WBeing)*	20. During the past 4 weeks, to what extent has your physical health or emotional problems interfered with your normal social activities with family, friends, neighbours, or groups?<br>(1) Not at all;<br>(2) Slightly;<br>(3) Moderately;<br>(4) Quite a bit;<br>(5) Extremely
21. How much bodily pain have you had during the past 4 weeks?<br>*(Domain: Phys Health)*	21. How much bodily pain have you had during the past 4 weeks?<br>(1) None;<br>(2) Very mild;<br>(3) Mild;<br>(4) Moderate;<br>(5) Severe;<br>(6) Very severe
22. During the past 4 weeks, how much did pain interfere with your normal work (including both work outside the home and housework)?<br>*(Domain: Phys Health)*	22. During the past 4 weeks, how much did pain interfere with your normal work (including both work outside the home and housework)?<br>(1) Not at all;<br>(2) A little bit;<br>(3) Moderately;<br>(4) Quite a bit;<br>(5) Extremely
These questions are about how you feel and how things have been with you during the past 4 weeks. For each question, please give the one answer that comes closest to the way you have been feeling.	

For peer review only

How much of the time during the past 4 weeks...<br>23. Did you feel full of pep?<br>*(Domain: Emotional WBeing)*	How much of the time during the past 4 weeks...<br>23. Did you feel full of pep?<br>(1) All of the time;<br>(2) Most of the time;<br>(3) A good bit of the time;<br>(4) Some of the time;<br>(5) A little of the time;<br>(6) None of the time
24. Have you been a very nervous person?<br>*(Domain: Emotional WBeing)*	24. Have you been a very nervous person?<br>Answer (1)-(6)
25. Have you felt so down in the dumps that nothing could cheer you up?<br>*(Domain: Emotional WBeing)*	25. Have you felt so down in the dumps that nothing could cheer you up?<br>Answer (1)-(6)
26. Have you felt calm and peaceful?<br>*(Domain: Emotional WBeing)*	26. Have you felt calm and peaceful?<br>Answer (1)-(6)
27. Did you have a lot of energy?<br>*(Domain: Emotional WBeing)*	27. Did you have a lot of energy?<br>Answer (1)-(6)
28. Have you felt downhearted and blue?<br>*(Domain: Emotional WBeing)*	28. Have you felt downhearted and blue?<br>Answer (1)-(6)
29. Did you feel worn out?<br>*(Domain: Emotional WBeing)*	29. Did you feel worn out?<br>Answer (1)-(6)
30. Have you been a happy person?<br>*(Domain: Emotional WBeing)*	30. Have you been a happy person?<br>Answer (1)-(6)
31. Did you feel tired?<br>*(Domain: Emotional WBeing)*	31. Did you feel tired?<br>Answer (1)-(6)
32. During the past 4 weeks, how much of the time has your physical health or emotional problems interfered with your social activities (like visiting with friends, relatives, etc.)?<br>*(Domain: Emotional WBeing)*	32. During the past 4 weeks, how much of the time has your physical health or emotional problems interfered with your social activities (like visiting with friends, relatives, etc.)?<br>(1) All of the time;<br>(2) Most of the time;<br>(3) Some of the time;<br>(4) A little of the time;<br>(5) None of the time
How TRUE or FALSE is each of the following statements for you.<br>33. I seem to get sick a little easier than other people<br>*(Domain: Phys Health)*	How TRUE or FALSE is each of the following statements for you.<br>33. I seem to get sick a little easier than other people<br>(1) Definitely true;<br>(2) Mostly true;<br>(3) Don't know;<br>(4) Mostly false;<br>(5) Definitely false
34. I am as healthy as anybody I know<br>*(Domain: Phys Health)*	34. I am as healthy as anybody I know<br>Answer (1)-(5)
35. I expect my health to get worse<br>*(Domain: Phys Health)*	35. I expect my health to get worse<br>Answer (1)-(5)
36. My health is excellent<br>*(Domain: Phys Health)*	36. My health is excellent<br>Answer (1)-(5)

For peer review only

Note that the answers to the 36 questions were recoded on a scale from 0 to 100 with 100 indicating the highest levels of health (ie negatively-phrased questions being coded in reverse polarity).

These were then divided into eight sub-domains (four physical health, four emotional well-being, with the recoded variables being summed then divided by the number of questions asked:

Domain	Physical Well-Being		Emotional Well-Being	
Sub-domain	Physical functioning	Pain	Role limitations due to emotional problems	Fatigue
	Role limitations due to physical health	General Health	Emotional Well-Being	Social Functioning

Cronbach's Alpha for the four Physical Well-Being measures is 0.838.

For the four Emotional Well-Being measures, Alpha=0.684.

For peer review only

CASP-12 (v2) Item	Wording
1-3 Control	1 My age prevents me from doing the things I would like to.<br>(1) Often; (2) Sometimes; (3) Not often;<br>(4) Never<br>2 I feel what happens to me is out of my control.<br>Answer (1)-(4)<br>3 I feel left out of things<br>Answer (1)-(4)
4-6 Autonomy	4 I can do the things I want to do.<br>Answer (1)-(4)<br>5 Family responsibil prevent me from doing what want to do.<br>Answer (1)-(4)<br>6 I feel that I can please myself what I do.<br>Answer (1)-(4)<br>6? Shortage of money stops CM doing things wants to do.<br>Answer (1)-(4)
7-9 Self-Realisation	7 I look forward to each day.<br>Answer (1)-(4)<br>8 I feel that my life has meaning.<br>Answer (1)-(4)<br>9 I enjoy the things that I do.<br>Answer (1)-(4)<br>9? On balance I look back on life with sense of happiness.<br>Answer (1)-(4)
10-12 Pleasure	10 I feel full of energy these days.<br>Answer (1)-(4)<br>11 I feel that life is full of opportunities.<br>Answer (1)-(4)<br>12 I feel that the future looks good for me.<br>Answer (1)-(4)

Cronbach’s Alpha for the twelve CASP items is 0.861.

For peer review only

Appendix 4 Rutter Behaviour scale, NCDS aged 16 (18-point version asked to mother)

Below are a series of descriptions of behaviour often shown by young people. Please ask the mother about each one and ring the appropriate number to show the degree to which this description is true of the study child:

0 Does not apply
1 Applies somewhat
2 Certainly applies

1	Restless, difficulty staying seated
2	Squirmy,fidgety child
3	Often destroys others property
4	Frequently fights, quarrelsome
5	Not much liked by other children
6	Often worries about things
7	Does things on own, rather solitary
8	Irritable, flies off the handle
9	Appears miserable,unhappy & tearful
10	Twitches,mannerisms,tics-face or body
11	Frequently sucks thumb or fingers
12	Frequently bites nails or fingers
13	Is often disobedient
14	Cannot settle more than a few moments
15	Fearful of new situations or things
16	Fussy, over particular
17	Often tells lies
18	Bullies other children

Max score=36

Cronbach's Alpha for age 16 Rutter Scale = 0.687.
Age 11 Rutter scale has 14 items; Alpha=0.682.
Age 7 Rutter scale has 14 items; Alpha=0.669.

For peer review only

Appendix 5:

Measurement models for the eight latent variables in the SEM analysis

(a) FAMDIFFS: Family material difficulties at Age 7 years (CFI=1.00, TLI=1.00, RMSEA=0.00)

	Indicator loading	Standard Error
Housing	0.46	0.02
Finance	0.79	0.02
Unemployment	0.69	0.02

(b) Age7cog: Cognition at Age 7 years (0.96, 0.88, 0.105)

Arithmetic	0.67	0.01
Copying designs	0.44	0.01
Draw-A-Man	0.48	0.01
Southgate reading	0.69	0.01

(c) Age11Cog: Cognition at age 11 years (1.0, 0.99, 0.018)

NFER General ability	0.78	0.01
Reading comprehens'n	0.65	0.01
Maths	0.79	0.01
Copying designs	0.12	0.01

(d) Age16cog: Cognition at Age 16 years (0.97, 0.91, 0.172)

Reading comprehens'n	0.82	0.01
Mathematics	0.83	0.00
Teacher ass't Eng Abil	0.85	0.00
Teacher ass't Math Abil	0.86	0.01

(e) Age50Cog: Cognition at age 50 years (0.98, 0.94, 0.068)

Letter Cancel'n Speed	0.12	0.01
Word Recall	0.80	0.01
Delayed word recall	0.80	0.01
Animal naming	0.85	0.01

(f) PWB: Physical Well-Being at age 50 years (0.99, 0.97, 0.07)

Physfunc	0.73	0.01
Lim_Phys	0.77	0.01
Pain	0.82	0.01
Gen_hlth	0.76	0.01

(g) EWB: Emotional Well-Being at age 50 years (0.99, 0.95, 0.077)

Lim_Emot	0.62	0.01
Fatig	0.86	0.01
Em_WB	0.84	0.01
Soc_func	0.12	0.02

For peer review only

(h) CASP12: Quality of Life at age 50 years (0.88, 0.85, 0.088)

Control1	0.39	0.01
Control2	0.55	0.01
Control3	0.55	0.01
Autonmy1	0.49	0.01
Autonmy2	0.38	0.01
Autonmy3	0.37	0.01
Pleasur1	0.75	0.01
Pleasur2	0.69	0.01
Pleasur3	0.72	0.01
SlfReal1	0.74	0.01
SlfReal2	0.72	0.01
SlfReal3	0.80	0.01

For peer review only

Appendix 6: Calculation of Indirect Effect and Total Effects in the Structural Equation Model

A Indirect effects from early-life circumstances to Age 50 outcomes (where total effect size >.01 and statistically significant)

Effects from	To	Total effect	Total indirect	Sig. direct effect? (p<.001)	Total effect significant?
SC Birth	Age 50 Cog	0.117	0.117	No	Yes
Birthwt	Age 50 Cog	0.047	0.030	No	Yes
Rutter7	Age 50 Cog	-0.065	-0.071	No	Yes
Rutter11	Age 50 Cog	-0.038	-0.025	No	Yes
Rutter16	Age 50 Cog	-0.018	-0.023	No	Yes
Masmoke	Age 50 Cog	-0.027	-0.027	No	Yes
Breastf	Age 50 Cog	0.039	0.039	No	Yes
SC Birth	PWB	0.081	0.048	No	Yes
Birthwt	PWB	0.011	0.011	No	Yes
Breastf	PWB	0.014	0.014	No	Yes
Rutter7	PWB	-0.043	-0.059	No	Yes
Rutter11	PWB	-0.081	-0.039	No	Yes
Rutter16	PWB	-0.074	-0.002	Yes	Yes
Masmoke	PWB	-0.010	-0.010	No	Yes
Rutter7	EWB	-0.046	-0.046	No	Yes
Rutter11	EWB	-0.080	-0.061	No	Yes
Rutter16	EWB	-0.133	-0.003	Yes	Yes
SC Birth	QoL	0.055	0.033	No	Yes
Rutter7	QoL	-0.048	-0.034	No	Yes
Rutter11	QoL	-0.049	-0.049	No	Yes
Rutter16	QoL	-0.103	-0.004	Yes	Yes

B Indirect effects from early-life circumstances to early mid-life outcomes (total effect size >.01 and statistically significant)

Effects from	To	Total effect	Total indirect	Sig. direct effect? (p<.001)	Total effect significant?
SC Birth	SC42yrs	0.167	0.113	Yes	Yes
SC Birth	Quals	0.184	0.135	Yes	Yes
Birthwt	SC42yrs	0.030	0.030	No	Yes
Birthwt	Quals	0.035	0.035	No	Yes
Masmoke	SC42yrs	-0.027	-0.027	No	Yes
Masmoke	Quals	-0.032	-0.032	No	Yes
Breastf	SC42yrs	0.039	0.039	No	Yes
Breastf	Quals	0.047	0.047	No	Yes

For peer review only

C Indirect effects from early life circumstances to Age 50 outcomes (total effect size <.01 or not statistically significant)

Effects from	to	Total effect	Total indirect	Sig. direct effect? (p<.001)	Total effect significant?
SC Birth	EWB	0.031	0.012	No	No
Birthwt	EWB	0.007	0.004	No	No
Masmoke	EWB	-0.003	-0.003	No	Yes
Breastf	EWB	0.005	0.005	No	Yes
Birthwt	QoL	0.003	0.006	No	No
Masmoke	QoL	-0.005	-0.005	No	Yes
Breastf	QoL	0.008	0.008	No	Yes

Commentary: Three direct influences stand out in Table A, each involving the Rutter scores at age 16 years (see Appx 4).

These are modest direct influences on PWB and QoL, but a relatively stronger negative influence on EWB at age 50 years.

Two direct influences stand out in Table B: from social class at birth to social class at 42 years and Qualifications obtained. The direct influences are shown in our estimated pathways diagram (Fig.2), but we also see in Table C there is an additional indirect effect (via childhood cognition at ages 7/11/16), over twice as large as the direct effect in both cases.

For peer review only

Appendix 7: Separate analyses by sex

Running the analyses separately by sex, we see that certain pathways are significant for one sex but not the other (p<.001) and certain path coefficients are notably different:

Men only (N=3815)

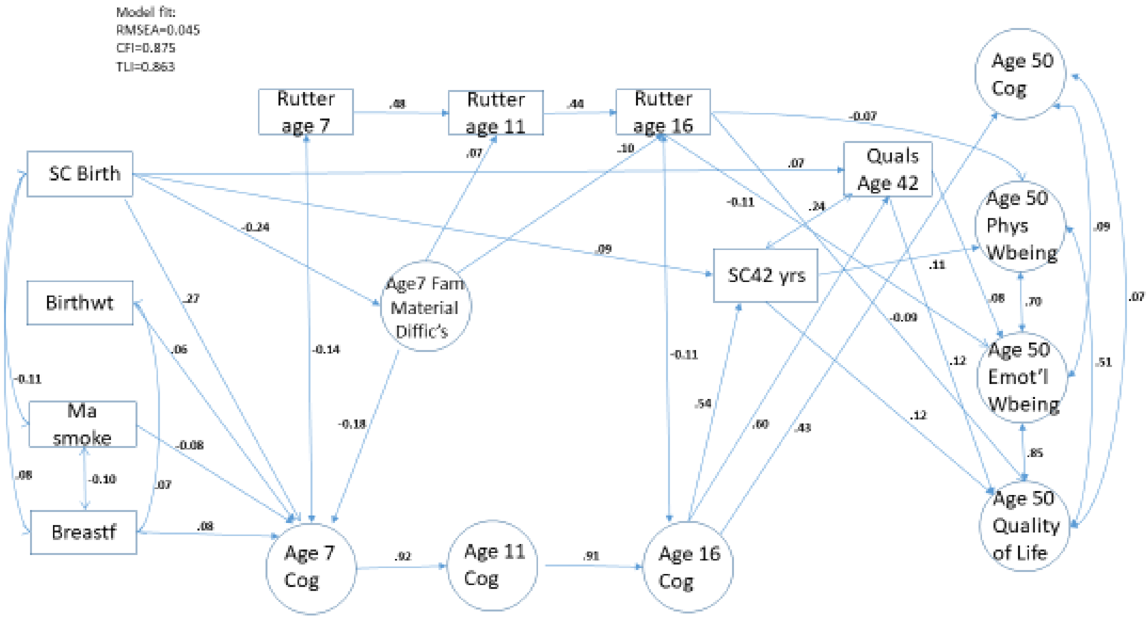

Women only (N=4209)

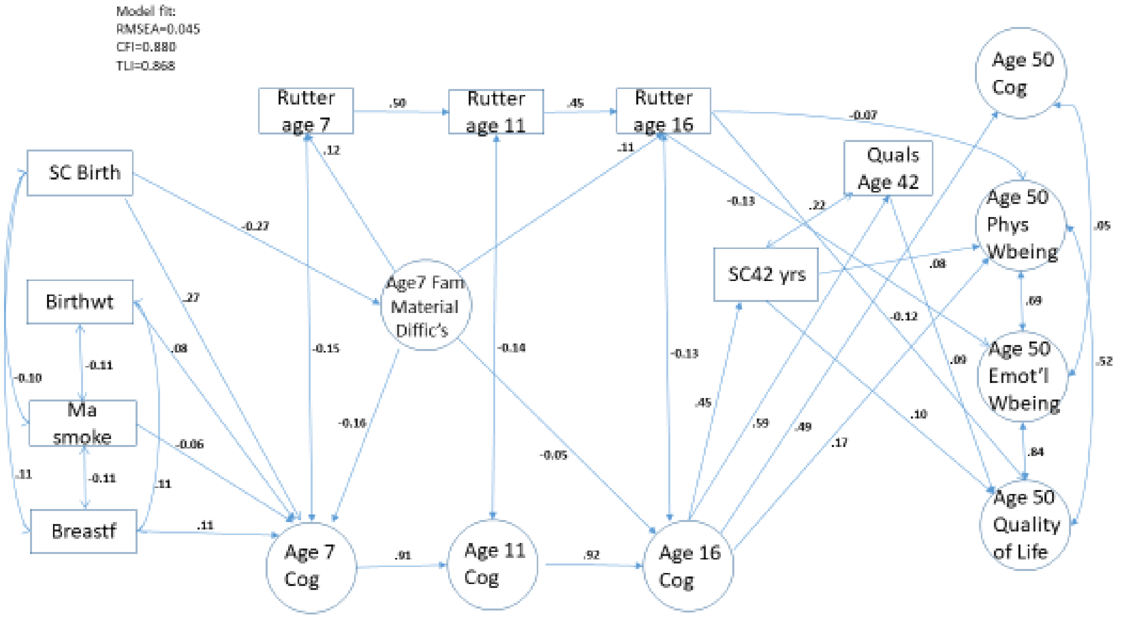

Appendix 8: Multigroup analysis by sex

An MPlus multigroup run by sex was performed as a sensitivity analysis, in which each of the 50 possible paths in Fig.1 was labelled m1-m50, and (in the female model) f1-f50, then a set of 50 'difference' variable was defined diff(i)=m(i)-f(i).

Testing the significance of these 50 'diff' variables, 6 were found to be significant, and the other 43 non-significant.

The MPlus multigroup model was then re-run, constraining all the 44 non-significant paths to be equal for males and females, but allowing all the other six paths to vary, thus increasing the parsimony of the model.

The six paths found to be significantly different between men and women are shown in this table:

	Men: Estimate	P-val	Women: Estimate	P-val
Age 50 Cog on Age 16 Cog	0.43	0.000	0.49	0.000
SC 42yrs on SC Birth	0.09	0.000	0.01	0.461
Phys WBeing on Age 16 Cog	0.07	0.013	0.17	0.000
Age 50 Cog on SC 42yrs	0.06	0.002	-0.02	-0.146
Quals Age 42 on Age 16 Cog	0.60	0.000	0.59	0.000
Age 16 Cog on Age 11 Cog	0.91	0.000	0.92	0.000

Model fit:

RMSEA 0.047
CFI 0.861
TLI 0.855

The first three paths are notable. The fourth is only significant at the .002 level, and in the case of the last two, the difference in the point estimates, though significant, is so small as to be unremarkable.

We see that women's Age 50 cognitive ability has a stronger link with Age 16 cognition (.49) than men's (.43), and that for men there is a direct link from SC Birth to SC 42 yrs (.09), a result not significant for women.

Finally, the link from Age 16 Cog to Phys WBeing is significant for women (.17), a result not significant for men.
